# Supplementary material for: Effects of multiple sclerosis on the audio-vestibular system: a systematic review
Source: BMJ Open. 2022 Aug 17;12(8):e060540. doi: 10.1136/bmjopen-2021-060540 (PMC9389089; doi:10.1136/bmjopen-2021-060540)
Supplement: Supplementary data [file bmjopen-2021-060540supp001.pdf]

Supplement

Section I: Literature Search Strategy

Database searched: Scopus

Date of coverage: 2000 to November 2021

Search undertaken: August 2020, September 2020, February 2021, November 2021

((TITLE-ABS-KEY("multiple sclerosis")AND (hearing OR auditory OR "hearing loss" OR "auditory function" OR “hearing dysfunction”)) OR (TITLE-ABS-KEY("multiple sclerosis") AND (dizziness OR vestibular OR vertigo OR equilibrium OR balance OR balanced OR “vestibular function”)

Database searched: Pubmed

Date of coverage: 2000 to November 2021

Search undertaken: August 2020, September 2020, February 2021, November 2021

(("multiple sclerosis"[MeSH Terms] AND ("auditorially"[All Fields] OR "auditory"[All Fields] OR ("hearing"[MeSH Terms] OR "hearing"[All Fields] OR "hearings"[All Fields]) OR "hearing loss"[All Fields] OR "hearing” [All Fields] OR "auditory function"[All Fields]) OR ("multiple sclerosis"[MeSH Terms] AND ("vestibular"[All Fields] OR ("dizziness"[MeSH Terms] OR "dizziness"[All Fields] OR "dizzy"[All Fields] OR "vertigo"[MeSH Terms] OR "vertigo"[All Fields]) OR ("vertigo"[MeSH Terms] OR "vertigo"[All Fields] OR "vertigos"[All Fields] OR "vertigoes"[All Fields]) OR ("equilibrium"[All Fields] OR "equilibriums"[All Fields]) OR ("balance"[All Fields] OR "balanced"[All Fields] OR "balances"[All Fields] OR "balancing"[All Fields]) OR "vestibular function"[All Fields]) AND

Translations

Multiple Sclerosis[MeSH Terms]: "multiple sclerosis"[MeSH Terms]

auditory: "auditorially"[All Fields] OR "auditory"[All Fields]

hearing: "hearing"[MeSH Terms] OR "hearing"[All Fields] OR "hearings"[All Fields]

multiple sclerosis[MeSH Terms]: "multiple sclerosis"[MeSH Terms]

dizziness: "dizziness"[MeSH Terms] OR "dizziness"[All Fields] OR "dizzy"[All Fields] OR "vertigo"[MeSH Terms] OR "vertigo"[All Fields]

vertigo: "vertigo"[MeSH Terms] OR "vertigo"[All Fields] OR "vertigos"[All Fields] OR "vertigoes"[All Fields]

equilibrium: "equilibrium"[All Fields] OR "equilibriums"[All Fields]

balance: "balance"[All Fields] OR "balanced"[All Fields] OR "balances"[All Fields] OR "balancing"[All Fields]

Database searched: Web of Science

Date of coverage: 2000 to November 2021

Search undertaken: August 2020, September 2020, February 2021, November 2021

Databases= WOS, BCI, BIOSIS, CCC, DRCI, DIIDW, KJD, MEDLINE, RSCI, SCIELO, ZOOREC, Timespan=2000-2021

#1 TS=("multiple sclerosis")

#2 TS=(auditory OR audio OR "auditory function" OR “auditory system”)

#3 TS=(hear* OR hearing OR "hearing loss")

#4 #2 OR #3

#5 #4 AND #1

#6 TS=(vestibular OR vestibul* OR "vestibular dysfunction" OR "vestibular system")

#7 TS=(vertigo)

#8 TS=(dizzy OR dizziness)

#9 TS=(balance OR balanced OR balances OR balanc*)

#10 #6 OR #7 OR #8 OR #9

#11 #1 AND #10

#12 #5 OR #11 AND

Database searched: NICE

Date of coverage: 2000 to November 2021

Search undertaken: August 2020, September 2020, February 2021, November 2021

(("multiple sclerosis") AND (hearing OR auditory OR "hearing loss" OR "auditory function" OR hear* OR “hearing dysfunction)) OR (("multiple sclerosis") AND (dizziness OR vestibular OR vertigo OR equilibrium OR balance OR balanced OR “vestibular function”))

Section II: Assessment of Bias

Table S1: *Assessment of Bias for case-control studies according to RoBANs*

Year	Author	Selection of participants	Confounding Variables	Measurement of Exposure	Blinding of the Outcome of the Assessment	Incomplete Outcome of Data	Selective Outcome Reporting
2002	Japaridze et al.[1]	?	+	+	-	+	+
2002	Sartucci and Logi[2]	+	+	+	-	+	+
2004	Alpini et al.[3]	-	?	+	-	+	+
2005	Aidar and Suzuki[4]	+	+	+	-	+	-
2007	Patkó et al.[5]	?	-	+	-	+	+
2008	Zeigelboim et al.[6]	?	+	+	-	+	+
2009	Lima et al.[7]	?	?	+	-	+	+
2010	Lewis et al.[8]	+	+	+	-	+	+
2010	Matas et al.[9]	+	+	+	-	+	+
2012	Doty et al.[10]	-	+	+	-	+	+
2012	Saberi et al.[11]	+	+	+	-	+	+
2013	Gabelić et al.[12]	+	+	+	-	+	+
2013	Harirchian et al.[13]	+	+	+	-	+	+
2014	Valadbeigi et al.[14]	+	+	+	-	+	+
2015	Pokryszko-Dragan et al.[15]	+	+	+	-	+	+
2018	Doty et al.[16]	-	+	+	-	+	+
2018	Kavasoğlu et al.[17]	+	?	+	-	+	+
2018	Koura et al.[18]	+	+	+	-	+	+
2019	Elbeltagy et al.[19]	-	+	+	-	+	+
2021	Rishiq et al.[20]	?	+	+	-	+	+
2021	Srinivasan et al.[21]	+	+	+	-	+	+
2021	Elmoazen et al.[22]	+	+	+	-	+	+

Green "+" = low risk of bias, red "-" = high risk of bias, yellow "?" = unclear risk of bias.

Table S2: *Risk of bias assessment of cohort studies by the Newcastle-Ottawa Quality Assessment form for cohort studies.*

Year	Author	Representativeness of exposed cohort	Selection of non-exposed cohort	Ascertainment of exposure	Outcome of interest not present at the beginning	Comparability of cohorts/confounders	Assessment of the outcome	Follow-up Length	Adequacy of follow-up of cohorts	Study quality
2002	Versino et al.[23]	*	*n/a*	*	*	*n/a*	-	*n/a*	*	?
2008	Burina et al.[24]	-	*n/a*	*	*n/a*	-	-	-	-	?
2009	Eleftheriadou et al.[25]	*	-	*	*	*	-	*	*	Poor
2010	Degirmenci et al.[26]	*	-	*	*	*	*	*	*	Good
2016	Kaytancı et al.[27]	-	-	*	-	*	-	*	*	Poor
2019	Di Mauro et al.[28]	*	-	*	*	*	-	*	*	Good

'' = low risk of bias, '-' = high risk of bias, "n/a" = the criteria does not apply to the paper, "?" = unknown quality of the paper due to the presence of "n/a" criteria.*

Table S3: *Risk of bias assessment of cross-sectional studies by the Newcastle-Ottawa Quality Assessment form which is adapted for cross-sectional studies.*

Year	Author	Representativeness of the sample	Sample Size	Non-respondents	Ascertainment of exposure (risk factor)	Comparability of groups/confounders	Assessment of the outcome	Statistical test	Study quality
2015	Parsa et al.[29]	-	-	-	*	*	-	*	Poor
2020	Yang and Liu[30]	*	-	*	*	*	-	*	Good
2020	Inojosa et al.[31]	*	-	*	*	*	-	*	Good
2020	Inojosa et al.[32]	*	-	*	*	*	-	*	Good
2021	Delphi et al.[33]	-	-	*	*	*	-	*	Good
2021	Cochrane et al.[34]	*	-	*	*	*	-	*	Good
2021	Cochrane et al.[35]	*	-	*	*	*	-	*	Good

'' = low risk of bias, '-' = high risk of bias.*

Section III: Multiple Sclerosis and Auditory Function

Table S4. Characteristics of studies examining the effects of MS on auditory function

Test Battery	*Author (Year)*	*Study Type*	*Sample Size*		*PwMS Mean Age (years)*	*PwMS Age range (Years)*	*% Female*	*MS Type (sample size)*	*MS Duration Mean (range) years*	*Results of PwMS compared to controls / normative data*
			PwMS	*Controls*						
ABR	Japaridze et al. (2002)[1]	Case-Control	40	33	30.5	18-57	77.5	?	?	65% Abnormal
ABR	Versino et al. (2002)[23]	Cohort	65	18	35.5	19-61	?	?	?	37.5% Abnormal
ABR	Burina et al. (2008)[24]	Cohort	60	NA	37.2	?	68.3	RRMS	?	95% Abnormal
ABR	Eleftheriadou et al. (2009)[25]	Cohort	46	40	40	20-66	45.6	RRMS	4.6	26% Abnormal
ABR	Lima et al. (2009)[7]	Case-control	25	NA	42.6 (female) 38 (male)	33-53 (female) 24-56 (male)	64.0	RRMS (14), PPMS (8), Unspecified Progressive (3)	?	30% Abnormal
ABR	Matas et al. (2010)[9]	Case-control	25	25	34.88	25-55	76.0	RRMS	4.25 (?)	Significantly different
ABR	Saberi et al. (2012)[11]	Case-control	60	38	29.9	?	73.3	?	3.2 (?)	20% Abnormal
ABR	Pokryszko-Dragan et al. (2015)[15]	Case-control	86	40	39.55	19-60	72.0	?	8.57 (1-30)	Significantly different
ABR	Kaytancı et al. (2016)[27]	Cohort	20	20	31.3	?	55.0	?	?	Significantly different
ABR	Di Mauro et al. (2019)[28]	Cohort	40	40	37	18-50	60.0	RRMS	0.8 (0.8-2)	No difference
ABR	Delphi et al. (2021)[33]	Cross-sectional	25	25	31.43	18-45	72.0	?	?	Significantly different
ABR	Rishiq et al. (2021)[20]	Case-control	11	9	49.5	34-68	72.7	RRMS (9), PPMS (1), SPMS (1)	11.9 (2-30)	Significantly different at high click rate
ABR	Srinivasan et al. (2021)[21]	Case-control	45	45	31.77	18-50	73.3	RRMS	6.1 (5.37)	Significantly different and 55.56 % abnormal
PTA	Lewis et al. (2010)[8]	Case-control	47	49	51.4	21-65	57.7 RRMS, 28.6 SPMS	RRMS (26), SPMS (21)	RRMS: 12.6 (2-43) SPMS: 23.3 (8-50)	Worse thresholds
PTA	Doty et al. (2012)[10]	Case-control	73	73	Males: 45.24 Females: 45.6	?	71.2	RRMS (57), PPMS (3), SPMS (6), Unspecified (7)	Males: 7.36 (?) Females: 7.84 (?)	No significant difference
PTA	Saberi et al. (2012)[11]	Case-control	60	38	29.9	?	73.3	?	3.2 (?)	Worse thresholds in female PwMS
PTA	Di Mauro et al. (2019)[28]	Cohort	40	40	37	18-50	60.0	RRMS	0.8 (0.8-2)	No significant difference
PTA	Srinivasan et al. (2021)[21]	Case-control	45	45	31.77	18-50	73.3	RRMS	6.1 (5.37)	Significantly different
TEOAEs & DPOAEs	Saberi et al. (2012)[11]	Case-control	60	38	29.9	?	73.3	?	3.2 (?)	No significant difference
TEOAEs & DPOAEs	Kaytancı et al. (2016)[27]	Cohort	20	20	31.3	?	55.0	?	?	No significant difference
TEOAEs & DPOAEs	Di Mauro et al. (2019)[28]	Cohort	40	40	37	18-50	60.0	RRMS	0.8 (0.8-2)	Significantly lower

Table S4 continued: Characteristics of studies examining the effects of MS on auditory function

Test Battery	Author (Year)	Study Type	Sample Size PwMS	Sample Size Controls	PwMS Mean Age (years)	PwMS Age range (Years)	% Female	MS Type (sample size)	MS Duration Mean (range) years	Results of PwMS compared to controls / normative data
WDS	Lewis et al. (2010)[8]	Case-control	47	49	51.4	21-65	57.7 RRMS, 28.6 SPMS	RRMS (26), SPMS (21)	RRMS: 12.6 (2-43) SPMS: 23.3 (8-50)	No significant difference
WDS	Valadbeigi et al. (2014)[[14]	Case-control	26	26	28.9	18-40	?	RRMS	?	Lower scores PwMS
WDS	Elbeltagy et al. (2019)[19]	Case-control	20	20	37.6	30-50	?	RRMS	?	Significantly lower
DPST	Valadbeigi et al. (2014)[14]	Case-control	26	26	28.9	18-40	?	RRMS	?	Significantly different
GIN	Valadbeigi et al. (2014)[14]	Case-control	26	26	28.9	18-40	?	RRMS	?	Significantly different
GIN	Elbeltagy et al. (2019)[19]	Case-control	20	20	37.6	30-50	?	RRMS	?	Significantly different
CAEP	Japaridze et al. (2002)[1]	Case-Control	40	33	30.5	18-57	77.5	?	?	30% Abnormal
CAEP	Matas et al. (2010)[9]	Case-control	25	25	34.88	25-55	76.0	RRMS	4.25 (?)	16% Abnormal

Abbreviations: PTA= Pure Tone Audiometry, ABR= Auditory Brainstem Response, OAE= Oto-acoustic Emissions, TEOAE= Transient Evoked Oto acoustic emission, DPOAE= Distortion Product Oto acoustic Emissions, CAEP= Cortical Auditory Evoked Potentials, SCP= Slow Cortical Potentials, GIN= Gaps in Noise test, WDS= Word Discrimination Scores, DPST= Duration pattern sequence test, MS= Multiple Sclerosis, PwMS = Persons with Multiple Sclerosis, ? = not reported.

Cells shaded in grey represent test results that were abnormal or showed a difference between PwMS and Controls

Extracted Pure Tone Audiometry Thresholds and Auditory Brainstem Response Results

Table S5: Pure Tone Audiometry Thresholds

Author (Year)	Group	Test Ear	PTA Threshold Per Frequency (Hz)													
			125		250		500		1000		2000		4000		8000	
			$\bar{x}$	SD	$\bar{x}$	SD	$\bar{x}$	SD	$\bar{x}$	SD	$\bar{x}$	SD	$\bar{x}$	SD	$\bar{x}$	SD
Doty et al. (2012)[10]	Male PwMS	Average Across Ears	Not Tested		15.87	5.39	14.05	5.94	12.52	7.43	12.79	8.97	20.03	11.71	17.02	17.26
	Male Controls				14.35	8.24	15.90	8.68	15.53	8.58	17.74	8.93	22.77	16.08	20.23	22.49
	Female PwMS				12.85	6.88	12.49	6.50	12.05	6.59	14.62	7.19	15.65	8.97	13.28	12.38
	Female Controls				11.28	7.77	12.52	7.60	10.45	6.82	11.62	8.17	13.92	8.79	12.65	12.60
Di Mauro et al. (2019)[28]	PwMS	Right	10.30	1.60	10.10	0.08	10.10	0.08	10.40	1.10	11.10	1.70	10.80	1.80	13.00	2.60
	Controls	Right	10.90	1.40	10.80	1.60	11.00	1.90	10.80	2.30	11.00	1.20	11.90	2.40	13.90	2.40
	PwMS	Left	10.30	1.70	10.90	1.20	10.40	1.90	10.40	1.30	10.80	1.10	11.40	1.70	12.50	2.20
	Controls	Left	10.90	1.40	10.80	1.60	11.00	1.30	10.90	2.10	11.10	1.90	11.90	2.80	13.90	2.40
Srinivasan et al. (2021)[21]	PwMS	Average Across Ears	Not Tested		12.22	6.40	13.11	5.92	13.61	5.90	12.55	7.19	13.66	8.06	16.16	9.08
	Controls				8.94	5.05	9.50	4.17	9.88	3.82	9.50	5.45	10.88	5.83	12.05	5.89

Abbreviations:
PwMS = Persons with Multiple Sclerosis, PTA = Pure Tone Audiometry, $\bar{x}$ = Mean, SD = Standard Deviation

Table S6: Auditory Brainstem Response Parameters and Results

Author (Year)	ABR Parameters							Peak Latency (ms)						Interpeak Latencies (ms)						Peak Amplitude (μV)			
	Click Polarity - Transducer	PR	PL	Ave.	BP Filter	Group	Test Ear	I		III		V		I-III		III-V		I-V		I		V	
								$\bar{x}$	SD	$\bar{x}$	SD	$\bar{x}$	SD	$\bar{x}$	SD	$\bar{x}$	SD	$\bar{x}$	SD	$\bar{x}$	SD	$\bar{x}$	SD
Lima et al. (2009)[7]	Rarefaction - TDH 39 Headphone	13.10	60 dB SL*	1024	100-3000	PwMS	Right	1.50	0.16	3.63	0.30	5.56	0.40	2.13	0.22	1.93	0.31	4.06	0.33	?	?	?	?
							Left	1.53	0.20	3.63	0.30	5.64	0.43	2.10	0.31	2.01	0.29	4.11	0.39	?	?	?	?
Matas et al. (2010)[9]	Rarefaction - TDH 39 Headphone	19.90	80 dB nHL	2000	100-3000	PwMS	Average Across Ears	1.53	0.13	3.72	0.31	5.78	0.37	2.21	0.26	2.03	0.27	4.26	0.35	?	?	?	?
						Controls		1.53	0.07	3.57	0.09	5.50	0.45	2.04	0.06	1.98	0.08	4.03	0.06	?	?	?	?
Pokryszko-Dragan et al. (2015)[15]	? - Earphone unspecified	20.30	65 dB SL*	200	150-3000	PwMS	Right	1.72	0.19	3.88	0.33	5.81	0.81	2.18	0.19	2.00	0.30	4.19	0.39	0.29	0.18	0.37	0.20
						Controls		1.70	0.14	3.85	0.16	5.73	0.23	2.14	0.12	1.87	0.18	4.02	0.20	0.30	0.10	0.46	0.19
						PwMS	Left	1.68	0.12	3.87	0.17	5.77	0.73	2.19	0.16	1.96	0.30	4.15	0.34	0.32	0.14	0.40	0.24
						Controls		1.69	0.13	3.83	0.12	5.70	0.21	2.13	0.14	1.87	0.17	4.00	0.19	0.30	0.14	0.44	0.16
Kaytancı et al. (2016)[27]	? - TDH 39 Headphone	13.00	70 dB nHL	1500	2000-4000	PwMS	Right	2.03	0.27	4.36	0.49	6.26	0.35	2.33	0.49	1.90	0.28	4.23	0.42	?	?	?	?
						Controls		1.45	0.30	3.52	0.31	5.38	0.53	2.07	0.44	1.85	0.48	3.92	0.53	?	?	?	?
						PwMS	Left	1.86	0.24	4.41	0.39	6.25	0.43	2.54	0.34	1.84	0.20	4.38	0.43	?	?	?	?
						Controls		1.47	0.24	3.40	0.43	5.39	0.46	1.92	0.48	1.99	0.53	3.91	0.47	?	?	?	?
						PwMS	IA Diff.	?	?	?	?	0.40	0.46	0.35	0.24	0.49	0.46	0.40	0.48	?	?	?	?
						Controls		?	?	?	?	0.15	0.12	0.34	0.35	0.27	0.30	0.19	0.16	?	?	?	?
Delphi et al. (2021)[33]	Rarefaction - ?	9.00	70 dB nHL	?	30-3000	PwMS	Right	1.89	0.11	4.25	0.14	6.32	0.13	2.30	0.11	2.21	0.09	4.48	0.13	?	?	?	?
						Controls		1.87	0.14	4.01	0.13	5.79	0.14	2.17	0.09	1.99	0.13	4.12	0.11	?	?	?	?
						PwMS	Left	1.80	0.15	4.30	0.15	6.19	0.14	2.29	0.08	2.19	0.14	4.51	0.11	?	?	?	?
						Controls		1.79	0.11	3.96	0.13	5.87	0.15	2.15	0.14	1.96	0.08	4.09	0.14	?	?	?	?
Srinivasan et al. (2021)[21]	Rarefaction – Insert earphones	21.10	80 dB nHL	1500	100-3000	PwMS	Average Across Ears	1.44	0.18	3.61	0.40	5.63	0.73	2.17	0.38	2.03	0.50	4.19	0.70	?	?	?	?
						Controls		1.43	0.16	3.48	0.19	5.30	0.24	2.04	0.17	1.82	0.18	3.86	0.21	?	?	?	?
		80.10				PwMS	Average Across Ears	?	?	?	?	6.12	0.72	?	?	?	?	?	?	?	?	?	?
						Controls		?	?	?	?	5.76	0.27	?	?	?	?	?	?	?	?	?	?

Abbreviations:
PR = Presentation Rate, PL = Presentation Level, Ave. = Number of Averages, BP = Band Pass, , $\bar{x}$ = Mean, SD = Standard Deviation, dB SL = Decibels Sensation Level, dB nHL = Decibels normal Hearing Level, PwMS = Persons with Multiple Sclerosis, IA Diff. = Inter-aural Difference, ? = not reported.
* dB SL reference to hearing thresholds (did not specify hearing thresholds to click or to pure tones)

Section IV: Multiple Sclerosis and Vestibular Function

Table S7. Characteristics of studies examining the effects of MS on vestibular function

Test Battery	Author (Year)	Study Type	Sample Size		Mean Age PwMS (years)	PwMS Age range (Years)	% Female	MS Type (sample size)	MS Duration Mean (range) years	Results of PwMS compared to controls / normative data
			PwMS	Controls						
cVEMP	Sartucci & Logi (2002)[2]	Case-Control	15	15	44.5	26-59	66.6	?	?	Significantly different
cVEMP	Versino et al. (2002)[23]	Cohort	70	18	35.5	19-61	?	?	?	31.4% Abnormal
cVEMP	Alpini et al. (2004)[3]	Case-control	40	25	38	17-71	57.5	?	?	70% Abnormal
cVEMP	Aidar and Suzuki (2005)[4]	Case-control	15	15	39.3	?	?	?	?	Significantly different
cVEMP	Patkó et al. (2007)[5]	Case-control	30	30	43.4	27-60	66.6	?	?	Significantly different
cVEMP	Eleftheriadou et al. (2009)[25]	Cohort	46	40	40	20-66	45.6	RRMS	4.6 (?)	Significantly different
cVEMP	Gabelić et al. (2013)[12]	Case control	30	15	?	?	46.6	RRMS	3.93 (0.2-21)	Significantly different
cVEMP	Harirchian et al. (2013)[13]	Case-control	20	20	30	20-40	50	RRMS<br>SPMS	?	70% Abnormal
cVEMP	Parsa et al. (2015)[29]	Cross-sectional	34	15	29.8	?	100	?	?	Significantly different
cVEMP	Kavasoğlu et al. (2018)[17]	Case-control	30	31	30	18-45	60.0	?	< 1 (?)	23.3% Abnormal<br>No significant difference
cVEMP	Koura and Hussein (2018)[18]	Case-control	20	10	36.80	?	65.0	?	4.4 (?)	100% Abnormal
cVEMP	Delphi et al. (2021)[33]	Cross-sectional	25	25	31.43	18-45	72.0	?	?	Significantly different
cVEMP	Elmoazen et al. (2021)[22]	Case-control	20	10	Brainstem lesions: 40<br>No Brainstem lesion: 34.1	?	?	?	?	Significantly different
cVEMP	Cochrane et al. (2021)[34,35]	Cross-sectional	40	20	42.4	21-55	88.0	RRMS	9.9	No significant difference
oVEMP	Gabelić et al. (2013)[12]	Case control	30	15	?	?	46.6	RRMS	3.93 (0.2-21)	Significantly different
oVEMP	Parsa et al. (2015)[29]	Cross-sectional	34	15	29.8	?	100	?	?	Significantly different
oVEMP	Elmoazen et al. (2021)[22]	Case-control	20	10	Brainstem lesions: 40<br>No Brainstem lesion: 34.1	?	?	?	?	Significantly different
oVEMP	Cochrane et al. (2021)[34,35]	Cross-sectional	40	20	42.4	21-55	88.0	RRMS	9.9	No significant difference
ENG	Zeigelboim et al. (2008)[6]	Case-control	30	0	42.23	27-64	80	RRMS	?	86.7% Abnormal
ENG	Degirmenci et al. (2010)[26]	Cohort	30	30	37.9	23-56	56.7	RRMS	?	90% Abnormal

Table S7 continued: Characteristics of studies examining the effects of MS on vestibular function

Test Battery	Author (Year)	Study Type	Sample Size		Mean Age PwMS (years)	PwMS Age range (Years)	% Female	MS Type (sample size)	MS Duration Mean (range) years	Results of PwMS compared to controls / normative data
			PwMS	Controls						
**Dynamic Posturography**	Doty et al. (2018)[16]	Case-control	58	72	Males:44.61<br>Females: 44.60	?	68.9	?	Male: 7.03 (?)<br>Female: 6.54 (?)	Significantly different
**Static Posturography**	Inojosa et al. (2020)[31,32]	Cross-sectional	99	30	35.01	18-50	68.7	?	5.5 (?)	Significantly different
	Yang and Liu (2020)[30]	Cross-sectional	30	25	50.8	?	76.7	?	14 (?)	Significantly different
**Rotary Chair**	Cochrane et al. (2021)[34,35]	Cross-sectional	40	20	42.4	21-55	88.0	RRMS	9.9	Significantly different

Abbreviations: cVEMP = Cervical Vestibular Evoked Myogenic Potentials, oVEMP = Ocular Vestibular Evoked Myogenic Potentials, MS= Multiple Sclerosis, PwMS = Persons with Multiple Sclerosis, ? = not reported.
Cells shaded in grey represent test results that were abnormal or showed a difference between PwMS and Controls.

Extracted Vestibular Evoked Myogenic Potentials Results

Table S8: Ocular Vestibular Evoked Myogenic Potentials Parameters and Results

Author (Year)	Stimulus (polarity) - Trasducer	PR (Hz)	PL	Ave.	BP Filter (Hz)	Group	Side	Peak Latency (ms) n1 x̄	n1 SD	p1 x̄	p1 SD	Amplitude (µV) n1-p1 x̄	n1-p1 SD
Gabelić et al. (2013)[12]	Click (?) - Headphone	1	130 dB SPL	100	5-1000	PwMS	Average Across sides	10.30	?	14.90	?	13.10	?
						Controls		9.5	?	14	?	13.3	?
Parsa et al. (2015)[29]	500 Hz Tone Burst (?) - Insert earphones	?	95 db nHL	?	?	PwMS with infratentorial plaque	Right	15.67	1.32	20.59	1.65	6.61	0.88
						PwMS without infratentorial plaque		12.21	1.00	16.90	1.32	6.61	0.60
						Controls		10.2	0.48	15.1	0.87	12.77	4.12
						PwMS with infratentorial plaque	Left	15.74	1.66	21.40	2.88	6.48	0.68
						PwMS without infratentorial plaque		11.78	0.89	16.92	0.82	6.82	0.75
						Controls		10.3	0.51	14.9	0.84	12.6	4.04
Elmoazen et al. (2021)[22]	500 Hz Tone Burst (condensation) -Supra aural headphones	5	95 dB nHL	150	1-1000	PwMS with Brainstem lesions	Average Across sides	11.43	1.42	16.86	1.79	3.59	3.82
						PwMS without Brainstem lesions		10.58	2.50	15.81	1.42	3.22	2.00
						Controls		10.09	0.92	14.71	1.20	2.76	1.28
Cochrane et al. (2021)[34]	Unspecified Tone Burst (?) – earphone unspecified	?	?	100	?	PwMS	?	8.50	1.70	12.90	2.20	5.20	3.50
						Controls		8.40	1.50	12.30	2.10	4.70	2.50

Abbreviations:
PR = Presentation Rate, PL = Presentation Level, Ave. = Number of Averages, BP = Band Pass, , x̄ = Mean, SD = Standard Deviation, dB SPL = Decibels Sound Pressure Level, dB nHL = Decibels normal Hearing Level, PwMS = Persons with Multiple Sclerosis, ? = not reported.

Table S9: Cervical Vestibular Evoked Myogenic Potentials Parameters and Results

Author (Year)	Stimulus (polarity) - Trasducer	PR (Hz)	PL	Ave.	BP Filter (Hz)	Group	Side	Peak Latency (ms) p13 x̄	p13 SD	n23 x̄	n23 SD	Amplitude (µV) p13-n23 x̄	p13-n23 SD
Sartucci and Logi (2002)[2]	Click (Rarefaction) - Headphone	3	140 dB SPL	256	10-1500	PwMS	Right	15.30	3.10	22.50	2.30	63.00	65.50
						Controls		12.5	0.8	21.3	1.5	186.4	82.8
						PwMS	Left	14.00	2.30	20.80	2.30	73.10	58.80
						Controls		12.3	0.9	20.8	1.3	141.1	85.8
Alpini et al. (2004)[3]	Click (Rarefaction) - Headphone	5	95 dB nHL + 70 dB SPL contralateral Masking	400	?	PwMS	Right	14.40	?	22.40	?	?	?
							Left	14.90	?	22.50	?	?	?
Aidar and Suzuki (2005)[4]	Click (Rarefaction) - Headphone	2	95 dB HL	200	20-1000	PwMS	Right	13.40	2.87	18.64	2 .67	12 .17	6 .50
						Controls		11 .12	1.64	15.15	2.51	7.54	5.95
						PwMS	Left	13.49	1 .77	17.84	1.27	13.30	3 .63
						Controls		10.76	1.59	14.86	2.85	7.23	4.76
Patkó et al. (2007)[5]	Click (Rarefaction) - Headphone	5	133 dB SPL	250	10-1600	PwMS	Right	14.40	1.50	21.30	3.20	11.61	3.63
						Controls		12.7	1.5	21.3	3.7	16.02	5.34
						PwMS	Left	14.8	1.5	21.2	3.2	11.07	2.87
						Controls		12.9	1.9	20.7	2.8	13.85	4.3
Eleftheriadou et al. (2009)[25]	Click (Rarefaction) - Headphone	5	105 dB HL	250	5-1500	PwMS with brainstem leasions	Average acrross sides	15.48	1.40	23.94	1.51	?	?
						PwMS without brainstem leasions		14.56	1.20	23.25	1.42	?	?
						Controls		12.67	1.1	21.66	1.11	?	?
Gabelić et al. (2013)[12]	Click (?) - Headphone	1	130 dB SPL	100	5-1000	PwMS	Average across sides	15.80	?	24.90	?	1.90	?
						Controls		14.6	?	22.1	?	1.6	?
Harirchian et al. (2013)[13]	Click (Rarefaction) - Headphone	2	95 db nHL + 40 dB nHL contralateral Masking	200	10-1000	PwMS	Right	22.20	9.30	30.02	11.20	?	?
						Controls		12.9	1.4	21.3	1.6	?	?
						PwMS	Left	20.20	7.05	30.71	16.20	?	?
						Controls		13.1	1.9	20.6	1.9	?	?
						PwMS	IA Diff.	2.05	8.80	0.70	10.90	?	?
						Controls		0.17	2.2	0.4	2.5	?	?

Table S9 Continued: Cervical Vestibular Evoked Myogenic Potentials Parameters and Results

Author (Year)	Stimulus (polarity) - Trasducer	PR (Hz)	PL	Ave.	BP Filter (Hz)	Group	Side	Peak Latency (ms)				Amplitude (µV)	
								p13		n23		p13-n23	
								$\bar{x}$	SD	$\bar{x}$	SD	$\bar{x}$	SD
Parsa et al. (2015)[29]	500 Hz Tone Burst (?) -Insert earphones	?	95 db nHL	?	?	PwMS with infratentorial plaque	Right	18.75	1.81	28.15	1.43	118.82	29.97
						PwMS without infratentorial plaque		17.44	1.03	26.89	1.87	135.00	20.31
						Controls		15.25	0.9	24.55	1.07	180	44.97
						PwMS with infratentorial plaque	Left	19.11	1.72	28.88	2.27	119.65	26.02
						PwMS without infratentorial plaque		17.21	1.05	26.87	1.36	133.78	22.89
						Controls		15.31	0.93	24.48	1.18	175	57.59
Kavasoğlu et al. (2017)[17]	Click (Rarefaction) - Headphone	5	100 dB nHL	100	10-1000	PwMS	Right	12.90	2.10	21.20	1.78	7.10	2.80
						Controls		12	0.9	20.6	1.95	7.7	3.1
						PwMS	Left	12.90	2.20	21.40	2.10	7.00	2.50
						Controls		12.03	0.96	20.76	1.63	7.6	3
Koura and Hussein (2018)[18]	500 Hz Tone Burst (Rarefaction) -TDH 39 Headphones	5.1	95 db HL	200	?	PwMS	Right	17.14	0.71	26.82	0.84	41.88	1. 71
						Controls		13.83	1.145	20.76	3.43	61.18	34.64
						PwMS	Left	17.20	0.56	27.17	0.75	54.95	28.27
						Controls		14.43	0.702	22.3	1.725	65.74	18.51
Delphi et al. (2021)[33]	500 Hz Tone Burst (?) -?	5.1	95 dB nHL	?	10-1200	PwMS	Right	18.20	1.93	27.37	2.45	121.34	29.05
						Controls		14.96	1.02	24.21	2.10	169.21	48.76
						PwMS	Left	18.95	2.01	27.21	2.80	-	-
						Controls		15.02	1.10	24.12	2.30	-	-
Elmoazen et al. (2021)[22]	500 Hz Tone Burst (condensation) -Supra aural headphones	5	95 dB nHL	150	1-1000	PwMS with Brainstem lesions	Average across sides	16.49	1.49	25.96	1.93	66.25	52.42
						PwMS without Brainstem lesions		15.21	1.57	23.97	2.06	93.12	78.01
						Controls		14.05	0.94	23.36	2.14	144.42	122.73
Cochrane et al. (2021)[34]	Unspecified Tone Burst (?) – earphone unspecified	?	?	?	?	PwMS	?	13.70	1.30	21.10	2.60	12.70	7.60
						Controls		13.30	0.50	20.60	1.80	16.50	7.10

Abbreviations:
PR = Presentation Rate, PL = Presentation Level, Ave. = Number of Averages, BP = Band Pass, , $\bar{x}$ = Mean, SD = Standard Deviation, dB SPL = Decibels Sound Pressure Level, dB HL = Decibels Heraing Level, dB nHL = Decibels normal Hearing Level, PwMS = Persons with Multiple Sclerosis, IA Diff. = Inter-aural Difference, ? = not reported. Cells Highlighted in Yellow indicate that "normalized amplitudes" were reported as opposed to absolute amplitudes reported by other studies. Cells Highlighted in Green indicate results were reported without specifying side or if they are averaged across sides.

1 Japaridze G, Shakarishvili R, Kevanishvili Z. Auditory brainstem, middle-latency, and slow cortical responses in multiple sclerosis. *Acta Neurol Scand* 2002;**106**:47–53. doi:10.1034/j.1600-0404.2002.01226.x

2 Sartucci F, Logi F. Vestibular-evoked myogenic potentials: A method to assess vestibulo-spinal conduction in multiple sclerosis patients. *Brain Res Bull* 2002;**59**:59–63. doi:10.1016/s0361-9230(02)00842-0

3 Alpini D, Pugnetti L, Caputo D, *et al.* Vestibular evoked myogenic potentials in multiple sclerosis: clinical and imaging correlations. *Mult Scler* 2004;**10**:316–21. doi:10.1191/1352458504ms1041oa

4 Aidar RC, Suzuki FA. Vestibular evoked myogenic potential: new perspectives in multiple sclerosis. *Braz J Otorhinolar* 2005;**71**:48–54. doi:10.1016/s1808-8694(15)31284-2

5 Patkó T, Simó M, Arányi Z. Vestibular click-evoked myogenic potentials: sensitivity and factors determining abnormality in patients with multiple sclerosis. *Mult Scler J* 2007;**13**:193–8. doi:10.1177/1352458506070940

6 Zeigelboim BS, Arruda WO, Mangabeira-Albernaz PL, *et al.* Vestibular findings in relapsing, remitting multiple sclerosis: a study of thirty patients. *Int Tinnitus J* 2008;**14**:139–45.

7 Lima TMA, Crato AN, Mancini PC, *et al.* Alterations in early auditory evoked potentials in multiple sclerosis patients. *Braz J Otorhinolar* 2009;**75**:177–81. doi:10.1016/s1808-8694(15)30775-8

8 Lewis MS, Lilly DJ, Hutter MM, *et al.* Audiometric hearing status of individuals with and without multiple sclerosis. *J Rehabilitation Res Dev* 2010;**47**:669–78. doi:10.1682/jrrd.2010.01.0010

9 Matas CG, Matas SL de A, Oliveira CRS de, *et al.* Auditory evoked potentials and multiple sclerosis. *Arq Neuro-psiquiat* 2010;**68**:528–34. doi:10.1590/s0004-282x2010000400010

10 Doty RL, Tourbier I, Davis S, *et al.* Pure-Tone Auditory Thresholds Are Not Chronically Elevated in Multiple Sclerosis. *Behav Neurosci* 2012;**126**:314–24. doi:10.1037/a0027046

11 Saberi A, Hatamian HR, Nemati S, *et al.* Hearing statement in multiple sclerosis: a case control study using auditory brainstem responses and otoacoustic emissions. *Acta Medica Iranica* 2012;**50**:679–83.

12 Gabeli T, Krbot M, Šefer AB, *et al.* Ocular and Cervical Vestibular Evoked Myogenic Potentials in Patients With Multiple Sclerosis. *J Clin Neurophysiol* 2013;**30**:86–91. doi:10.1097/wnp.0b013e31827eda0c

13 Harirchian MH, Karimi N, Nafisi S, *et al.* Vestibular evoked myogenic potential for diagnoses of multiple sclerosis: is it beneficial? *Medicinski Glasnik Official Publ Medical Assoc Zenica-doboj Canton Bosnia Herzegovina* 2013;**10**:321–6.

14 Valadbeigi A, Weisi F, Rohbakhsh N, *et al.* Central auditory processing and word discrimination in patients with multiple sclerosis. *Eur Arch Oto-rhino-l* 2014;**271**:2891–6. doi:10.1007/s00405-013-2776-6

15 Pokryszko-Dragan A, Bilinska M, Gruszka E, *et al.* Assessment of visual and auditory evoked potentials in multiple sclerosis patients with and without fatigue. *Neurol Sci* 2015;**36**:235–42. doi:10.1007/s10072-014-1953-8

16 Doty RL, MacGillivray MR, Talab H, *et al.* Balance in multiple sclerosis: relationship to central brain regions. *Exp Brain Res* 2018;**236**:2739–50. doi:10.1007/s00221-018-5332-1

17 Kavasoğlu G, Gökçay F, Yüceyar N, *et al.* Cervical vestibular-evoked myogenic potentials in patients with multiple sclerosis: sensitive in detecting brainstem involvement? *Neurol Sci* 2018;**39**:365–71. doi:10.1007/s10072-017-3215-z

18 Koura R, Hussein M. Vestibular-evoked myogenic potential: an easy neurophysiological tool for evaluating brain stem involvement in multiple sclerosis. *Egypt J Otolaryngology* 2018;**34**:144–8. doi:10.4103/ejo.ejo_73_17

19 Elbeltagy R, Gad N, Ismail M. Central auditory function in multiple sclerosis patients. *Indian J Otology* 2019;**25**:90–6. doi:10.4103/indianjotol.indianjotol_80_18

20 Rishiq D, Harkrider A, Springer C, *et al.* Click-evoked and speech-evoked auditory brainstem responses from individuals with multiple sclerosis. *Neurosci Lett* 2021;**740**:135460. doi:10.1016/j.neulet.2020.135460

21 Srinivasan VS, Krishna R, Munirathinam BR. Effectiveness of Brainstem Auditory Evoked Potentials Scoring in Evaluating Brainstem Dysfunction and Disability Among Individuals With Multiple Sclerosis. *Am J Audiol* 2021;**30**:255–65. doi:10.1044/2020_aja-20-00155

22 Elmoazen D, Kozou H, Gawies M, *et al.* Assessment of Cervical and Ocular Vestibular Evoked Myogenic Potentials in Multiple Sclerosis PatientsS. *Egyptian Journal of Ear, Nose, Throat and Allied Sciences* 2021;**22**:1–9. doi:10.21608/ejentas.2020.44108.1262

23 Versino M, Colnaghi S, Callieco R, *et al.* Vestibular evoked myogenic potentials in multiple sclerosis patients. *Clin Neurophysiol* 2002;**113**:1464–9. doi:10.1016/s1388-2457(02)00155-4

24 Burina A, Sinanović O, Smajlović D, *et al.* Some Aspects of Balance Disorder in Patients with Multiple Sclerosis. *Bosnian J Basic Med* 2008;**8**:80–5. doi:10.17305/bjbms.2008.3003

25 Eleftheriadou A, Deftereos SN, Zarikas V, *et al.* The diagnostic value of earlier and later components of Vestibular Evoked Myogenic Potentials (VEMP) in Multiple Sclerosis. *J Vestib Res* 2009;**19**:59–66. doi:10.3233/ves-2009-0342

26 Degirmenci E, Bir LS, Ardic FN. Clinical and electronystagmographical evaluation of vestibular symptoms in relapsing remitting multiple sclerosis. *Neurol Res* 2010;**32**:986–91. doi:10.1179/016164110x12681290831405

27 Kaytancı E, Ozdamar OI, Acar GO, *et al.* Evaluation of transiently evoked otoacoustic emissions and auditory brainstem responses in patients with multiple sclerosis. *Ear Nose Throat J* 2016;**95**:E12–7.

28 Di Mauro RD, Di Girolamo SD, Ralli M, *et al.* Subclinical cochlear dysfunction in newly diagnosed relapsing-remitting multiple sclerosis. *Mult Scler Relat Dis* 2019;**33**:55–60. doi:10.1016/j.msard.2019.05.020

29 Parsa MS, Mohammadkhani G, Hajabolhassani F, *et al.* Cervical and ocular vestibular evoked myogenic potentials in multiple sclerosis participants. *Medical J Islamic Repub Iran* 2015;**29**:164.

30 Yang F, Liu X. Relative importance of vision and proprioception in maintaining standing balance in people with multiple sclerosis. *Mult Scler Relat Dis* 2020;**39**:101901. doi:10.1016/j.msard.2019.101901

31 Inojosa H, Schriefer D, Klöditz A, *et al.* Balance Testing in Multiple Sclerosis—Improving Neurological Assessment With Static Posturography? *Front Neurol* 2020;**11**:135. doi:10.3389/fneur.2020.00135

32 Inojosa H, Schriefer D, Trentzsch K, *et al.* Visual Feedback and Postural Control in Multiple Sclerosis. *J Clin Medicine* 2020;**9**:1291. doi:10.3390/jcm9051291

33 Delphi M, Sayaf M, Taheri F, *et al.* Audio and vestibular analyses in patient with multiple sclerosis: a case–control study. *Hear Balance Commun* 2021;**19**:1–6. doi:10.1080/21695717.2021.1933318

34 Cochrane GD, Christy JB, Motl RW. Comprehensive Clinical Assessment of Vestibular Function in Multiple Sclerosis. *J Neurol Phys Ther* 2021;**45**:228–34. doi:10.1097/npt.0000000000000358

35 Cochrane GD, Christy JB, Sandroff BM, *et al.* Cognitive and Central Vestibular Functions Correlate in People With Multiple Sclerosis. *Neurorehab Neural Re* 2021;**35**:1030–8. doi:10.1177/15459683211046268
