# Supplementary material for: Effects of multiple sclerosis on the audio-vestibular system: a systematic review
Source: BMJ Open. 2022 Aug 17;12(8):e060540. doi: 10.1136/bmjopen-2021-060540 (PMC9389089; doi:10.1136/bmjopen-2021-060540)
Supplement: Author's manuscript [file bmjopen-2021-060540.draft_revisions.pdf]

BMJ Open

BMJ Open is committed to open peer review. As part of this commitment we make the peer review history of every article we publish publicly available.

When an article is published we post the peer reviewers' comments and the authors' responses online. We also post the versions of the paper that were used during peer review. These are the versions that the peer review comments apply to.

The versions of the paper that follow are the versions that were submitted during the peer review process. They are not the versions of record or the final published versions. They should not be cited or distributed as the published version of this manuscript.

BMJ Open is an open access journal and the full, final, typeset and author-corrected version of record of the manuscript is available on our site with no access controls, subscription charges or pay-per-view fees (http://bmjopen.bmj.com).

If you have any questions on BMJ Open's open peer review process please email info.bmjopen@bmj.com

The effects of Multiple Sclerosis on the audio-vestibular system: A systematic review

Journal:	*BMJ Open*
Manuscript ID	bmjopen-2021-060540
Article Type:	Original research
Date Submitted by the Author:	23-Dec-2021

Complete List of Authors:	Gür, Evrim; The University of Manchester, Manchester Centre for Audiology and Deafness (ManCAD), School of Health Sciences, Ellen Wilkinson Building Manchester, UK M13 9PL<br>Binkhamis, Ghada; King Fahad Medical City, Communication and Swallowing Disorders; The University of Manchester, Manchester Centre for Audiology and Deafness (ManCAD)<br>Kluk, Karolina; The University of Manchester, Manchester Centre for Audiology and Deafness (ManCAD), School of Health Sciences, Ellen Wilkinson Building Manchester, UK M13 9PL

Keywords:	Audiology < OTOLARYNGOLOGY, Adult otolaryngology < OTOLARYNGOLOGY, Multiple sclerosis < NEUROLOGY

I, the Submitting Author has the right to grant and does grant on behalf of all authors of the Work (as defined in the below author licence), an exclusive licence and/or a non-exclusive licence for contributions from authors who are: i) UK Crown employees; ii) where BMJ has agreed a CC-BY licence shall apply, and/or iii) in accordance with the terms applicable for US Federal Government officers or employees acting as part of their official duties; on a worldwide, perpetual, irrevocable, royalty-free basis to BMJ Publishing Group Ltd ("BMJ") its licensees and where the relevant Journal is co-owned by BMJ to the co-owners of the Journal, to publish the Work in this journal and any other BMJ products and to exploit all rights, as set out in our licence.

The Submitting Author accepts and understands that any supply made under these terms is made by BMJ to the Submitting Author unless you are acting as an employee on behalf of your employer or a postgraduate student of an affiliated institution which is paying any applicable article publishing charge ("APC") for Open Access articles. Where the Submitting Author wishes to make the Work available on an Open Access basis (and intends to pay the relevant APC), the terms of reuse of such Open Access shall be governed by a Creative Commons licence – details of these licences and which Creative Commons licence will apply to this Work are set out in our licence referred to above.

Other than as permitted in any relevant BMJ Author's Self Archiving Policies, I confirm this Work has not been accepted for publication elsewhere, is not being considered for publication elsewhere and does not duplicate material already published. I confirm all authors consent to publication of this Work and authorise the granting of this licence.

For peer review only

The effects of Multiple Sclerosis on the audio-vestibular system: A systematic review

Evrim Gür[1,*], Ghada BinKhamis[1,2,*] and Karolina Kluk[1]

[1]*Manchester Centre for Audiology and Deafness, Manchester Academic Health Science Centre, University of Manchester, Manchester, UK;* [2]*Department of Communication and Swallowing Disorders, King Fahad Medical City, Riyadh, Saudi Arabia*

*E.G. and G.B. equally contributed to this work

Address for correspondence:

Ghada BinKhamis: ghada.binkhamis@manchester.ac.uk / gbinkhamis@gmail.com

Karolina Kluk: Karolina.Kluk@manchester.ac.uk

Evrim Gür: evrimguur@gmail.com,

Manchester Centre for Audiology and Deafness (ManCAD), School of Health Sciences, Faculty of Biology Medicine and Health, Room B2.15, Ellen Wilkinson Building, University of Manchester, Oxford Road, Manchester, M13 9PL, United Kingdom.

ORCiD:

1. Ghada BinKhamis: 0000-0002-7696-1073
2. Karolina Kluk: 0000-0003-3638-2787

Word count: 5787

For peer review only

ABSTRACT

Objective: Systematically investigate the effects of Multiple Sclerosis (MS) on the audio-vestibular system.

Design: Systematic review.

Setting: PUBMED, SCOPUS, NICE and Web of Science were searched for studies between 2000 and 21st November 2021.

Eligibility criteria: randomised control trials, cohort, case-control, observational, retrospective studies in English investigating audio-vestibular conditions in persons with MS (PwMS), any MS type, aged ≥ 18 years, evaluated with at least one of the selected outcomes. Study selection and assessments of bias were independently conducted by two reviewers.

Participants: 35 studies were included. Auditory function was evaluated in 714 PwMS and 501 controls, vestibular function was evaluated in 682 PwMS and 446 controls.

Primary and secondary outcomes: Pure Tone Audiometry, Auditory Brainstem Response, Otoacoustic Emissions, Cortical Auditory Evoked Potentials, Functional MRI assessing auditory function, Vestibular Evoked Myogenic Potentials, Videonystagmography, Electonystagmography, Posturography, Rotary Chair. Secondary: Gaps in Noise test, Word Discrimination Scores, Duration Pattern Sequence Test.

Results: peripheral auditory function results were contradictory between studies; some found abnormalities in PwMS, and others found no differences. Tests of brainstem and central auditory functions were more consistently found to be abnormal in PwMS. Most vestibular tests were reported as abnormal in PwMS, abnormalities were either peripheral or central or both. However, quantitative analyses could not be performed due to discrepancies between studies in results reporting and in test stimulus and recording parameters.

Conclusions: Although abnormal test results on auditory and vestibular tests were noted in PwMS, specific effects of MS on the audio-vestibular system could not be determined due to

For peer review only

the differences between studies that restricted the ability to conduct any quantitative analyses. Further research with consistent reporting and consistent stimulus and recording parameters are needed to be able to quantify the effects of MS on the auditory and vestibular systems.

Prospero Registration: CRD42020180094

Strengths and limitations of this study:

- This study provides a comprehensive systematic review of the literature on MS and the auditory and vestibular systems from 2000 until 21st November 2021.
- This is the only systematic review that investigated the effects of MS on the audio-vestibular system.
- Differences between studies in reporting of results and in test procedures restricted the ability to conduct any form of quantitative analyses.

Keywords: Multiple sclerosis, auditory function, vestibular function, audio-vestibular system

For peer review only - http://bmjopen.bmj.com/site/about/guidelines.xhtml

The effects of Multiple Sclerosis on the audio-vestibular system: A systematic review

Multiple Sclerosis (MS) is a chronic, immune-mediated, inflammatory disease that causes a neurodegenerative process in the Central Nervous System (CNS).[1] Pathogenesis of MS is explained by the autoimmune attack against the CNS that results in axonal damage, olygodentrycide loss and demyelination in the CNS.[2] The hallmark of MS is known as plaques; focal areas of demyelination; however, axonal injury or loss also occurs.[3] MS plaques can occur in the white matter of the brain (predominantly), cerebral cortex including subpial regions, spinal cord and optic nerve.[4] There are four main subtypes of MS: Relapsing-remitting (RRMS), Primary-Progressive (PPMS), Secondary Progressive (SPMS) and Progressive Relapsing (PRMS). The clinical course of MS varies based on the region and volume of the demyelination plaques. Mainly, optic neuritis, double vision, tremor, ataxic gait, weakness and numbness in one or more limbs occur during the course of the disease.[5]

Hearing loss and balance disorders may result from demyelination plaques in the central auditory and vestibular pathways;[6] for example, plaques may occur in the brainstem, where both efferent and afferent auditory and vestibular pathways are present.[6] It has been suggested that peripheral auditory organs may also be affected in persons with MS (PwMS), specifically, that the migration of microglia from the central brain regions to more peripheral neural connections and the internal auditory canal can cause demyelination in inner ear structures.[7–9] Reports on the prevalence of audio-vestibular symptoms in PwMS vary in the literature. For example, one study reported a prevalence of audio-vestibular symptoms between 1% and 28%.[10] Whereas others reported that vertigo is seen in 20 – 35 % of PwMS, and hearing loss is seen in 1 – 17 % of PwMS.[11–14] While a recent systematic review on Magnetic Resonance Imaging (MRI) and auditory tests found that 25 % of PwMS had either sudden or progressive hearing loss.[10]

For peer review only

For peer review only - http://bmjopen.bmj.com/site/about/guidelines.xhtml

MS-related hearing loss is always sensorineural hearing loss (SNHL) that can be progressive, fluctuating or sudden; progression can occur in cases of progressive auditory pathway involvement in the degenerative process, and fluctuation may be related to the relapsing-remitting phases of MS.[14,15] Several studies showed worse Pure Tone Audiometry (PTA) thresholds in PwMS compared to healthy controls;[16–18] however, the differences between PwMS and controls were small (e.g. 4 - 10 dB on average). Alternatively, Doty et al. (2012) did not find a difference in PTA thresholds between PwMS and healthy controls.[19] According to Di Stadio et al. (2018), PTA thresholds in PwMS may be misleading as many will experience functional recovery from hearing loss.[10] They also found that all PwMS with sudden SNHL showed abnormal Auditory Brainstem Responses (ABR) such as abnormal morphology and prolonged wave latencies, with no improvement in ABRs after the recovery of PTA thresholds.[10] Lima et al. (2009) also found abnormal ABRs in 30 % of PwMS.[20] While Di Mauro et al. (2019) found no differences in ABRs between PwMS and healthy controls.[21] Nevertheless, hearing loss may be an underestimated condition in PwMS; this might be related to its temporary nature; however, it has been suggested that sudden hearing loss could be considered an "early detector" of MS.[10] Additionally, the involvement of auditory pathways in the demyelination process may affect speech understanding and temporal processing; however, literature in this area is limited. For example, Valadbeigi et al. (2014) found that PwMS had poorer word recognition scores in noise and an increased approximate threshold plus lower percent correct answers on the Gaps-In-Noise (GIN) test compared to healthy controls.[22] Similarly, Elbeltagy et al. (2019) found that PwMS showed poorer performance on the GIN test compared to healthy controls.[23] Although the literature suggests a potential effect of MS on auditory function including hearing, speech understanding and temporal processing, the results varied and therefore available literature needs to be combined and summarised to better understand the effects of MS on the auditory system.

For peer review only - http://bmjopen.bmj.com/site/about/guidelines.xhtml

MS-related vestibular symptoms may be peripheral, central or both. Studies have shown that the vestibular nuclei and the root entry zone of the eighth cranial nerve are one of the most common neuroanatomic locations for inflammatory demyelination;[24] in addition, peripheral vestibular deficits may also coexist.[25] Most studies consistently showed abnormal Electronystagmography (ENG) results in PwMS;[26–29] however, selection criteria and assessment techniques differed between studies. Other studies that investigated the vestibular pathway using Vestibular Evoked Myogenic Potentials (VEMPs) in PwMS found latency delays in both ocular (oVEMPs) and cervical (cVEMPs) VEMPs, likely resulting from a decrease in the conduction velocity of nerve fibres that may be caused by demyelination.[30,31] Abnormality in VEMP results including latency delays, reduced amplitudes and inter-aural side differences, were described by a considerable number of studies;[32–41] however, VEMP stimulus and recording parameters differed between studies and different studies investigated different VEMP components. Further studies investigated the functional integrity of the vestibular system in PwMS via static and dynamic posturography. Inojosa et al. (2020) found greater instability on static posturography in PwMS than in the control group.[42,43] This poorer performance on posturography in PwMS was also shown by others.[44] Although literature has shown abnormalities on different tests of the vestibular system in PwMS, studies had different participant inclusion criteria and used different test procedures. Therefore, results from the literature with their similarities and differences need to be combined and summarised to obtain a clearer picture of the effects of MS on the vestibular system.

Numerous studies performed different audio-vestibular test batteries in PwMS. These studies aimed to show possible MS related changes in auditory or vestibular function. However, results from literature that investigated auditory function varied, as some indicated no differences, while others reported considerable differences between PwMS and healthy controls. While results from literature on the effects of MS on auditory function were inconsistent, literature on

For peer review only - http://bmjopen.bmj.com/site/about/guidelines.xhtml

the effects of MS on vestibular function generally showed abnormalities in PwMS. However, the effects of MS on central versus peripheral vestibular function and what to expect to find on results of different vestibular tests in PwMS are still unclear. Therefore, literature needs to be combined and summarised to better understand the potential effects of MS on the auditory and/or vestibular systems. If auditory and/or vestibular disorders in PwMS are better understood, early identification and early intervention could occur, resulting in improved quality of life. This highlights the need for this systematic review on the effects of MS on the audio-vestibular system. We hypothesise that PwMS experience auditory and vestibular disorders at a higher rate than the general population. This systematic review aims to investigate this hypothesis by evaluating the effects of MS on the audio-vestibular system through examining and synthesising current literature.

METHODS

This systematic review followed the Preferred Reporting Items for Systematic reviews and Meta-Analyses (PRISMA) statement,[45] and was pre-registered in PROSPERO (CRD42020180094)

Patient and public involvement

No patient involved

Research ethics approval

As new participant data were not collected, Ethics Approval was not needed.

Data Sources and Search Strategy

A systematic search was conducted in the following electronic bibliographic databases: PubMed, NICE, Scopus and Web of Science using the following search terms: "Multiple Sclerosis and Auditory Function" or "Multiple Sclerosis and Vestibular Function" or "Multiple Sclerosis and hearing loss" or "Multiple Sclerosis and dizziness" or "Multiple Sclerosis and

For peer review only - http://bmjopen.bmj.com/site/about/guidelines.xhtml

vertigo". A total of 21 searches were performed by the first author up until November 21st 2021. No restrictions regarding language and date were applied at the initial search with 3876 studies identified. After manually removing duplicates, the search resulted in 3207 results.

Animal research, case reports, studies without full text in English, and studies published before the year 2000 were excluded. Any entries that did not include the following keywords "Multiple Sclerosis", "dizziness", "auditory function", "vestibular", "vertigo" or "hearing loss" in the title or abstract were excluded. A total of 90 studies remained and were independently reviewed by two reviewers (E.G. and G.B.). Any disagreements were resolved by discussion or by involving a third reviewer (K.K.) until a consensus was reached.

Inclusion and exclusion criteria

No geographical restrictions were applied. All studies that investigated audio-vestibular conditions in adults with MS were included. Participant age was restricted to 18 years and above because of maturational effects on outcomes of some audio-vestibular tests. Participants aged 18 years and older diagnosed with any MS type (RRMS, SPMS, PPMS, PRMS) were defined as the population being studied. Evaluation of auditory function was restricted to the following measures: Pure Tone Audiometry (PTA), Auditory Brainstem Response (ABR), Otoacoustic Emissions (OAEs: Transient Evoked (TEOAE) and Distortion Product (DPOAE)), Cortical Auditory Evoked Potentials (CAEP) and Functional MRI assessing auditory function. Evaluation of vestibular function was restricted to the following measures: Vestibular Evoked Myogenic Potentials (VEMPs) including Cervical (cVEMPs) and Ocular (oVEMPs), Videonystagmography (VNG), Electonystagmography (ENG), Posturography and Rotary Chair. Above test batteries were defined as primary outcomes, the secondary outcomes were defined as measures of speech perception and temporal processing, specifically; Gaps in Noise test (GIN), Word Discrimination Scores (WDS) and Duration Pattern Sequence Test (DPST).

For peer review only - http://bmjopen.bmj.com/site/about/guidelines.xhtml

Studies were eligible for inclusion if they met the following criteria: 1) Participants were diagnosed with one type of MS; 2) one or more of the above-described tests were used to evaluate auditory or vestibular function; 3) any of the following study types: randomised controlled trials, cohort, case-controlled, observational, or retrospective studies; 4) results from PwMS were either compared to a control group or to normative data. Studies were excluded based on the following: 1) Participants under the age of 18 years with MS; 2) study was not a research study (reviews, editorials, case studies); 3) participants were diagnosed with comorbid diseases.

Assessment of bias

Risk of bias assessment was done independently by two reviewers (E.G. and G.B.). Case-control studies were assessed using the Risk of Bias Assessment Tool for Nonrandomized Studies (RoBANS [46]) to examine the selection of participants, confounding variables, intervention measurement, blinding of outcome assessment, intervention measurement, incomplete outcome data and selective outcome reporting. Cohort studies were assessed with the Newcastle Ottawa Scale (NOS [47]). A judgement of 'low risk' of bias, 'high risk' or bias, or 'unclear risk' of bias was provided for each domain. Cross-sectional studies were evaluated with the NOS adapted for cross-sectional studies.[48] Any disagreements were resolved by discussion or by involving the third reviewer (K.K.) until a consensus was reached.

Data extraction and synthesis

Data extraction was undertaken by the first author. The following data were extracted when available: 1) Study details: aims and design. 2) Population: recruitment methods; diagnosis and inclusion/exclusion criteria; syndrome differentiation; course of disease; number of participants: screened, eligible, enrolled, included in the analysis; age; gender. 3) Details of the pathology: specific lesion site, primary or secondary, stable or progressive, additional handicaps related to MS. 4) Methodology: test procedure, stimulus and recording parameters,

For peer review only - http://bmjopen.bmj.com/site/about/guidelines.xhtml

electrode impendence and inter-electrode impedance levels. 5) Test results, e.g. PTA thresholds, VEMP amplitudes, ABR peak latencies, etc.

E.G. extracted the data and G.B. confirmed the accuracy of all extracted data. Any disagreements were resolved by discussion between the two reviewers. Per pre-registered protocol, investigators were not contacted for unreported data or additional information due to time constraints and given current COVID-19 pandemic related global circumstances.

Criteria for data synthesis: studies that used the same test procedure (either PTA, ABR or VEMP), minimum five participants in each study, minimum two studies for each test method, and the same stimulus and recording parameters were used. Mean, Standard Deviation (SD), and/or individual values were extracted when available, however, the effect size could not be calculated due to inconsistencies between studies in stimulus and recording parameters or in how results were reported.

For peer review only

RESULTS

Description of included studies

3876 studies were identified initially, and 1484 studies were identified after duplicate removal and primary limitations (Figure 1); 1055 were excluded because they did not relate to audio-vestibular conditions, 42 due to no full text availability, 33 because they were not in English, 177 due to the use of other test batteries than those in the inclusion criteria, and 87 were excluded because they did not have an abstract. The remaining 90 studies were examined for inclusion, eight were excluded because they were case reports, 22 did not evaluate audio-vestibular conditions, six used other test batteries than those in the inclusion criteria, 13 included participants under 18 years of age, and six additional duplicates were removed.

A total of 35 studies underwent full-length review. Fourteen evaluated the auditory system, 18 evaluated the vestibular system and three evaluated both auditory and vestibular systems.

For peer review only - http://bmjopen.bmj.com/site/about/guidelines.xhtml

Assessment of bias

Case-control studies assessed with RoBANS (Supplement Table S1) were judged as low risk of bias in most categories, except for blinding of outcome assessment that was judged as high risk of bias in all 22 studies. Cohort studies assessed with NOS (Supplement Table S2) were judged as either good (n = 2), poor (n = 2), or unknown (n = 2, rating could not be assigned due to one or more category that was judged as "not applicable" e.g. two studies were evaluated as not applicable for "selection of the non-exposed group" due to lack of a control group). Cross-sectional studies were assessed using NOS adapted for cross-sectional (Supplement Table S3) and most were judged as good (n = 6) with one judged as poor.

MS and auditory function

Seventeen studies investigated the auditory systems involvement in PwMS (Table 1): three assessed OAEs, five assessed PTA, 13 evaluated ABRs, two investigated CAEPs, and three of the 17 studies performed speech perception and/or temporal processing tests. No studies that conducted functional MRI to assess auditory function were located.

Three studies compared OAEs in PwMS and healthy controls with a total number of 218 participants, 120 PwMS and 98 healthy controls. Two studies did not find differences between PwMS and controls in TEOAEs and/or DPOAEs.[13,49] However, the third study found weaker responses in both TEOAEs and DPOAEs in PwMS compared to healthy controls.[21]

Five studies performed PTA on PwMS to assess hearing levels. A total of 510 participants (265 PwMS and 245 controls) were evaluated with PTA, sample size varied from 80 to 146 and age range was between 18 and 65 years. Two out of five studies reported no significant difference in PTA thresholds between PwMS and controls,[19,21]. One reported worse PTA thresholds only in female PwMS compared to female controls[13] and two reported worse thresholds in PwMS compared to controls,[18,50] with one finding worse thresholds in participants with SPMS when compared to RRMS.[18] Three of five studies provided mean and SD values of

For peer review only

For peer review only - http://bmjopen.bmj.com/site/about/guidelines.xhtml

PTA thresholds (Supplement Table S4). However, reported values could not be pooled because the way that authors presented the data differed between each other. For instance, Doty et al. (2012) reported average PTA thresholds across right and left ears for each frequency in male and female participants separately,[19] while Di Mauro et al. (2019) reported values for each ear separately for all participants.[21]

Thirteen studies assessed ABRs (Table 1) and included 881 participants (548 PwMS and 333 controls), sample size varied from 25 to 126 and age range was between 18 and 68 years. Twelve of the 13 studies reported abnormalities in different ABR components in PwMS as follows:

- Prolonged absolute latencies of wave I[49] III and V.[49–52]
- Prolonged interpeak latencies of I–III,[13,32,34,50–55] III–V,[13,32,34,50,52–55] and I–V.[20,32,49–53,55]
- Interaural interpeak latency differences for I–III and I–V[32] and III–V.[32,53]
- Absent waves III and V.[32,34,50,53,54]
- Absent waves at high stimulus presentation rates.[56]
- Reduced wave V amplitude.[55]
- Increased I/III and I/V amplitude ratios.[53]
- Reduced V/I amplitude ratio.[50,52]
- Poor waveform morphology and/or only wave I within normal limits.[20]

Only Di Mauro et al. (2019) did not find any differences in ABRs between PwMS and controls.[21] Six out of 13 studies shared mean, and SD values for latencies, amplitudes and/or inter-aural latency differences (Supplement Table S5); however, data could not be combined, and the effect size calculation was not performed due to the variability in stimulus and recording parameters. For example, Matas et al. (2010) presented rarefaction clicks at 80 dB nHL with a

For peer review only

presentation rate of 19.9 clicks per second,[51] while Kaytancı et al. (2016) presented clicks (polarity unspecified) at 70 dB nHL with a presentation rate of 13.0 clicks per second.[49]

Cortical evoked potentials were evaluated by two studies using two different tests (Table 1). Japaridze et al. (2002) reported 30% abnormality in slow cortical potentials (SCPs); abnormalities were either absent peaks or prolonged peak latencies.[53] Matas et al. (2010) assessed P300 peak latency (defined as a cognitive potential) and did not find any significant differences between PwMS and controls; however, percentage of abnormalities in P300 results was higher in the PwMS (16%) compared to controls (0%).[51]

Three studies assessed speech perception and temporal processing (Table 1) with total of 188 participants, 93 PwMS and 95 healthy controls and an age range of 18-65 years. Two of the three found that PwMS had poorer WDS in noise and an increased approximate threshold plus lower percent correct answers on the GIN test compared to healthy controls;[22,23] and one of the two also found that PwMS had lower percent correct answers on the DPST.[22] However, the third study found no significant difference between PwMS and controls on WDS in quiet.[18]

For peer review only

For peer review only - http://bmjopen.bmj.com/site/about/guidelines.xhtml

Table 1. *Characteristics of studies examining the effects of MS on auditory function*

Author (Year)	*Study Type*	*Sample Size*		*PwMS Mean Age (years)*	*PwMS Age range (Years)*	*% Female*	*MS Type (sample size)*	*MS Duration Mean (range) years*	*Test Battery*	*Results of PwMS compared to controls / normative data*
		PwMS	*Controls*							
Japaridze et al. (2002)[53]	Case-Control	40	33	30.5	18-57	77.5	?	?	ABR	65% Abnormal
									CAEP (SCP)	30% Abnormal
Versino et al. (2002)[32]	Cohort	65	18	35.5	19-61	?	?	?	ABR	37.5% Abnormal
Burina et al. (2008)[54]	Cohort	60	NA	37.2	?	68.3	RRMS	?	ABR	95% Abnormal
Eleftheriadou et al. (2009)[34]	Cohort	46	40	40	20-66	45.6	RRMS	4.6	ABR	26% Abnormal
Lima et al. (2009)[20]	Case-control	25	NA	42.6 (female) 38 (male)	33-53 (female) 24-56 (male)	64.0	RRMS (14), PPMS (8), Unspecified Progressive (3)	?	ABR	30% Abnormal
Lewis et al. (2010)[18]	Case-control	47	49	51.4	21-65	57.7 RRMS, 28.6 SPMS	RRMS (26), SPMS (21)	RRMS: 12.6 (2-43) SPMS: 23.3 (8-50)	PTA	Worse thresholds
									WDS	No significant difference
Matas et al. (2010)[51]	Case-control	25	25	34.88	25-55	76.0	RRMS	4.25 (?)	ABR	Significantly different
									CAEP (P300)	16% Abnormal
Doty et al. (2012)[19]	Case-control	73	73	Males: 45.24 Females: 45.6	?	71.2	RRMS (57), PPMS (3), SPMS (6), Unspecified (7)	Males: 7.36 (?) Females: 7.84 (?)	PTA	No significant difference
Saberi et al. (2012)[13]	Case-control	60	38	29.9	?	73.3	?	3.2 (?)	PTA	Worse thresholds in female PwMS
									TEOAE & DPOAE	No significant difference
									ABR	20% Abnormal
Valadbeigi et al. (2014)[22]	Case-control	26	26	28.9	18-40	?	RRMS	?	GIN	Significantly different
									WDS	Lower scores PwMS
									DPST	Significantly different
Pokryszko-Dragan et al. (2015)[55]	Case-control	86	40	39.55	19-60	72.0	?	8.57 (1-30)	ABR	Significantly different
Kaytancı et al. (2016)[49]	Cohort	20	20	31.3	?	55.0	?	?	TEOAE	No significant difference
									ABR	Significantly different
Di Mauro et al. (2019)[21]	Cohort	40	40	37	18-50	60.0	RRMS	0.8 (0.8-2)	PTA	No significant difference
									TEOAE & DPOAE	Significantly lower
									ABR	No difference
Elbeltagy et al. (2019)[23]	Case-control	20	20	37.6	30-50	?	RRMS	?	GIN	Significantly different
									WDS	Significantly lower
Delphi et al. (2021)[52]	Cross-sectional	25	25	31.43	18-45	72.0	?	?	ABR	Significantly different
Rishiq et al. (2021)[56]	Case-control	11	9	49.5	34-68	72.7	RRMS (9), PPMS (1), SPMS (1)	11.9 (2-30)	ABR	Significantly different at high click rate
Srinivasan et al. (2021)[50]	Case-control	45	45	31.77	18-50	73.3	RRMS	6.1 (5.37)	PTA	Significantly different
									ABR	Significantly different and 55.56 % abnormal

Abbreviations: PTA= Pure Tone Audiometry, ABR= Auditory Brainstem Response, OAE= Oto-acoustic Emissions, TEOAE= Transient Evoked Oto acoustic emission, DPOAE= Distortion Product Oto acoustic Emissions, CAEP= Cortical Auditory Evoked Potentials, SCP= Slow Cortical Potentials, GIN= Gaps in Noise test, WDS= Word Discrimination Scores, DPST= Duration pattern sequence test, MS= Multiple Sclerosis, PwMS = Persons with Multiple Sclerosis, ? = not reported.
Cells shaded in grey represent test results that were abnormal or showed a difference between PwMS and Controls

For peer review only

MS and vestibular function

A total of 21 studies that investigated the vestibular system in PwMS were identified (Table 2). Two studies investigated vestibular function using ENG; three used static posturography, one used dynamic posturography, 14 used cVEMPs and four also used oVEMPs, and two used Rotary Chair testing.

A total of 90 participants (60 PwMS and 30 healthy controls) were evaluated using ENG, age ranged between 24 and 64. Both studies found ENG abnormalities in the peripheral and central vestibular systems in PwMS.[29,57] Degirmenci et al. (2010) noted that 90% of PwMS showed ENG abnormalities compared to 6.7% of the controls, and there was a significant difference between groups.[29] Abnormalities suggestive of central vestibular pathology were found in 83.3% of PwMS and in 6.7% of controls, and abnormalities suggestive of peripheral vestibular pathology were found in 36.7% of PwMS and in 0% of controls.[29] Zeigelboim et al. (2008) also reported ENG abnormalities in 86.7% (83.4% Peripheral) of their PwMS.[57]

Four studies assessed posturography with a total of 314 participants (187 PwMS and 127 controls). All four studies found significant differences between PwMS and controls.[42,43,58,59] Two studies reported static posturography results for the same sample,[42,43] they noted increased delineated area, average sway and speed of sway for both open and closed eyes in PwMS compared to controls. A third study also conducted static posturography and found that sway area and path length were significantly greater in PwMS than in controls.[58] The fourth study evaluated dynamic posturography and found longer motor control test latencies, lower equilibrium scores on the sensory organisation tests, and a compromised ability to use three sensory inputs (somatosensory, vestibular, and visual preference) in PwMS compared to controls.[59]

A total of 14 studies assessed VEMPs and included 724 participants (435 PwMS and 289 healthy controls), sample size varied from 30 to 88, and age range was between 18 and 71 years.

For peer review only - http://bmjopen.bmj.com/site/about/guidelines.xhtml

Four studies evaluated both oVEMPs and cVEMPs[39,60–62] and 10 studies evaluated cVEMPs.[32–34,52,63–68] Thirteen of the 14 studies reported abnormalities in different VEMP components in PwMS as follows:

- Prolonged latencies of p13,[32–34,39,52,60,62–68] n23,[32,34,39,52,60,62–68] and p1 and n1.[39,60,62]
- Reduced p13-n23 amplitude.[32,52,63,65]
- Inter-side asymmetry for p13,[32,33,65] and n23 and n23 latencies.[32]
- Absent waves.[34,64,65,68]

Ten of 14 studies compared VEMPs in PwMS to controls.[34,39,52,60–65,67] Except for two,[61,67] all found a significant difference between PwMS and controls. The remaining VEMP studies used data from controls as normative data.[32,33,66,68]

Thirteen studies shared mean and SD values of p13, n23, p1 and n1 latencies (Supplement Tables S6 and S7). However, data could not be analysed because intensity level, stimulus type, stimulus polarity and other VEMP recording parameters differed between studies. For instance, Sartucci and Logi (2002) presented clicks at 140 dBSPL at a rate of three per second,[63] Aidar and Suzuki (2005) presented clicks at 95 dBHL at a rate of two per second,[64] Kavasoğlu et al. (2017) presented clicks at 100 dBnHL at a rate of five per second,[67] and Koura and Hussein (2018) presented a 500-Hz tone burst at 95 dBHL.[68]

Two studies conducted Rotary Chair testing. However, they tested and reported results from the same sample.[61,69] Forty PwMS and 20 controls were tested, results showed that PwMS had lower vestibular-ocular reflex (VOR) cancellation gain with a visual target and larger variances in their responses on both the subjective visual vertical and horizontal (SVV/H) tests compared to controls.[61] Also, that PwMS performed worse than controls on central vestibular measures but not on peripheral.[69]

For peer review only

Table 2. *Characteristics of studies examining the effects of MS on vestibular function*

Author (Year)	*Study Type*	*Sample Size*		*Mean Age PwMS (years)*	*PwMS Age range (Years)*	*% Female*	*MS Type (sample size)*	*MS Duration Mean (range) years*	*Test Battery*	*Results of PwMS compared to controls / normative data*
		PwMS	*Controls*							
Sartucci & Logi (2002)[63]	Case-Control	15	15	44.5	26-59	66.6	?	?	cVEMP	Significantly different
Versino et al. (2002)[32]	Cohort	70	18	35.5	19-61	?	?	?	cVEMP	31.4% Abnormal
Alpini et al. (2004)[33]	Case-control	40	25	38	17-71	57.5	?	?	cVEMP	70% Abnormal
Aidar and Suzuki (2005)[64]	Case-control	15	15	39.3	?	?	?	?	cVEMP	Significantly different
Patkó et al. (2007)[65]	Case-control	30	30	43.4	27-60	66.6	?	?	cVEMP	Significantly different
Zeigelboim et al. (2008)[57]	Case-control	30	0	42.23	27-64	80	RRMS	?	ENG	86.7% Abnormal
Eleftheriadou et al. (2009)[34]	Cohort	46	40	40	20-66	45.6	RRMS	4.6 (?)	cVEMP	Significantly different
Degirmenci et al. (2010)[29]	Cohort	30	30	37.9	23-56	56.7	RRMS	?	ENG	90% Abnormal
Gabelić et al. (2013)[60]	Case control	30	15	?	?	46.6	RRMS	3.93 (0.2-21)	cVEMP<br>oVEMP	Significantly different<br>Significantly different
Harirchian et al. (2013)[66]	Case-control	20	20	30	20-40	50	RRMS<br>SPMS	?	cVEMP	70% Abnormal
Parsa et al. (2015)[39]	Cross-sectional	34	15	29.8	?	100	?	?	cVEMP<br>oVEMP	Significantly different<br>Significantly different
Doty et al. (2018)[59]	Case-control	58	72	Males:44.61<br>Females: 44.60	?	68.9	?	Male: 7.03 (?)<br>Female: 6.54 (?)	Dynamic Posturography	Significantly different
Kavasoğlu et al. (2018)[67]	Case-control	30	31	30	18-45	60.0	?	< 1 (?)	cVEMP	23.3% Abnormal<br>No significant difference
Koura and Hussein (2018)[68]	Case-control	20	10	36.80	?	65.0	?	4.4 (?)	cVEMP	100% Abnormal
Inojosa et al. (2020)[42,43]	Cross-sectional	99	30	35.01	18-50	68.7	?	5.5 (?)	Static Posturography	Significantly different
Yang and Liu (2020)[58]	Cross-sectional	30	25	50.8	?	76.7	?	14 (?)	Static Posturography	Significantly different
Delphi et al. (2021)[52]	Cross-sectional	25	25	31.43	18-45	72.0	?	?	cVEMP	Significantly different
Elmoazen et al. (2021)[62]	Case-control	20	10	Brainstem lesions: 40<br>No Brainstem lesion: 34.1	?	?	?	?	cVEMP<br>oVEMP	Significantly different<br>Significantly different
Cochrane et al. (2021)[61,69]	Cross-sectional	40	20	42.4	21-55	88.0	RRMS	9.9	Rotary Chair<br>cVEMP<br>oVEMP	Significantly different<br>No significant difference<br>No significant difference

Abbreviations: cVEMP = Cervical Vestibular Evoked Myogenic Potentials, oVEMP = Ocular Vestibular Evoked Myogenic Potentials, MS= Multiple Sclerosis, PwMS = Persons with Multiple Sclerosis, ? = not reported. Cells shaded in grey represent test results that were abnormal or showed a difference between PwMS and Controls.

For peer review only - http://bmjopen.bmj.com/site/about/guidelines.xhtml

DISCUSSION

In this systematic review, 35 studies were reviewed: 22 case-control studies, six cohort studies, and seven cross-sectional studies. The aim was to investigate the effects of MS on the audio-vestibular system by examining and synthesising current literature that used different auditory and vestibular tests. Additionally, this systematic review presents an overview of the last 21 years of literature specific to MS in the context of the auditory and vestibular systems. The selected outcome measures for the auditory system were: OAE, PTA, ABR, CAEP, WDS, GIN, DPST. Vestibular system outcome measures were: VNG, ENG, Rotary Chair, VEMPs, Static and dynamic Posturography. The included studies on auditory function evaluated 1215 individuals in total, 714 PwMS and 501 healthy controls; the included studies on vestibular function covered 1128 individuals, 682 PwMS and 446 healthy controls. Included studies assessed participants who were over the age of 18, and the geographical location of individuals was not limited to a specific continent, country or hemisphere.

Neither meta-analyses nor effect size calculations could be performed due to the inconsistencies across studies in reporting results or in test stimulus and recording parameters that lead to the inability to combine their data (Supplement Tables S4 – S7).

Auditory system and MS

The specific effects of MS on the auditory system, based on results of auditory tests included in this systematic review, could not be established. Effect of MS on the peripheral auditory system, as demonstrated by OAEs, was only found by one of the three included studies.[21] Di Mauro et al. (2019) reported abnormal OAEs with normal ABRs in participants newly diagnosed with RRMS, showing cochlear rather than retrocochlear involvement.[21] They suggested that this cochlear dysfunction may be caused by a demyelinating process in the brainstem’s medial olivocochlear bunch that innervates outer hair cells[70] resulting in decreased OAE amplitudes.[21] However, reduced OAE amplitudes were not reported by

For peer review only

others, and it is unclear why Di Mauro et al. (2019) found differences between PwMS and controls, and both Kaytancı et al. (2016) and Saberi et al. (2012) did not.[13,49] Therefore, there is very limited evidence to date on the effects of MS on OAEs, and there is need for further investigations. Also, effects of MS on PTA thresholds could not be defined.

Three of five included studies reported worse PTA thresholds in PwMS compared to controls.[13,18,50] However, Srinivasan et al. (2021) included only participants with normal hearing, i.e. test sample was biased towards better PTA,[50] therefore the difference between PwMS and controls, although statistically significant, is not clinically significant. Apart from Di Mauro et al. (2019), who found no difference between PwMS and controls but only included participants with normal hearing that were recently diagnosed with RRMS,[21] reasons behind differences in PTA results between studies are unclear. Type and duration of MS do not appear to be factors, as those who found a difference included participants with a mixture of MS types and MS durations. For example, Lewis et al. (2010) included participants (n = 47) with RRMS (mean MS duration: 12.6 years) and SPMS (mean MS duration: 23.3 years),[18] and Saberi et al. (2012) included PwMS (n = 60, MS type not reported) with a mean MS duration of 3.2 years,[13] and they both found differences in PTA thresholds between PwMS and controls. While Doty et al. (2012) did not find any difference in PTA thresholds with the inclusion of participants (n = 73) with different MS types (RRMS, PPMS, SPMS, Unspecified MS) and a mean MS duration of 7.36 years in males and 7.84 years in females.[19] Therefore, conclusions on the effects of MS on PTA thresholds cannot be reached given the limited literature and the inability to combine results for a meta-analysis. Thus, there is a clear need for further studies investigating changes in PTA thresholds in PwMS.

The effects of MS on the brainstem auditory pathway as demonstrated by ABRs were more evident; 12 of 13 included studies found abnormalities in different ABR components in PwMS.[13,20,32,34,49–56] Demyelination plaques tend to occur in the brainstem, and the

For peer review only

For peer review only - http://bmjopen.bmj.com/site/about/guidelines.xhtml

impairment of the auditory olivocochlear pathways by demyelination is possible.[6] Therefore, ABR abnormalities such as prolonged latencies are thought to be related to the effects of MS plaques in the brainstem on the integrity of the auditory pathways.[10] Abnormal ABR findings in PwMS reported by studies included in this review were consistent with previous literature.[27,71] Only Di Mauro et al. (2019) found no differences in ABRs between PwMS and controls; however, PwMS with hearing loss or brainstem lesions in their MRI were excluded from the study, plus the authors included only participants newly diagnosed with RRMS.[21] Matas et al. (2010) included normally hearing participants with RRMS (mean MS duration: 4.25 years) and found differences in ABRs between PwMS and controls.[51] Burina et al. (2008) also included participants with RRMS and brainstem or cerebellum lesions and found abnormal ABRs.[54] Therefore, the reasons Di Mauro et al. (2019) did not find differences in ABRs between PwMS and controls are likely related to the short duration of MS and the exclusion of participants with brainstem lesions. Although most studies that investigated ABRs in PwMS found abnormalities, the nature of these abnormalities could not be established. This is due to the differences between studies in reported ABR measures (e.g. absolute peak latencies versus peak amplitudes) and in stimulus and recording parameters (e.g. presentation levels, stimulus polarity, transducer). Therefore, there is a clear need for consistency across studies to better understand the effects of MS on ABRs.

Furthermore, the effects of MS plaques on the integrity of the central auditory pathway as measured by CAEPs was much less studied, only two studies were included in this systematic review.[51,53] Both found abnormalities suggesting possible cortical involvement in PwMS; however, these studies could not be compared, and data could not be synthesised due to the use of different testing techniques. Therefore, further studies are needed to better understand the effects of MS on CAEPs.

For peer review only

For peer review only - http://bmjopen.bmj.com/site/about/guidelines.xhtml

The effects of MS on speech perception and temporal processing were also infrequently studied, three studies were included in this systematic review.[18,22,23] Two of the three found abnormalities on WDS tests in PwMS, both studies that found abnormalities performed WDS testing in noise,[22,23] and the study that did not find abnormal WDS tested in quiet.[18] It is therefore likely that Lewis et al. (2010) did not find abnormal WDS in PwMS because they did not test in background noise.[18] This is supported by Valadbeigi et al.'s (2014) findings of worse WDS in noise than in quiet in PwMS.[22] These abnormalities in WDS are possibly related to MS lesions in the central auditory pathways. Any degeneration caused by MS plaques could lead to a misperception of the acoustic signal leading to poorer performance on WDS.[72] Additionally, the two studies that performed the GIN test found abnormalities in PwMS indicating lower temporal resolution performance than in controls.[22,23] A previous study suggested that persons with lesions in the central auditory system had worse temporal resolution performance and therefore poorer performance on the GIN test compared to healthy controls.[73] Therefore, it is possible that temporal resolution is affected by MS plaques in the CNS. Since the evidence regarding abnormalities in speech perception and temporal processing in PwMS was limited, this systematic review concludes that there is need for further research to evaluate MS's effect on functional auditory performance.

Vestibular System and MS

All studies included in this systematic review found abnormalities on vestibular tests suggesting that the vestibular system may be more affected by MS than the auditory system. First, the two included studies that performed ENGs in PwMS found abnormalities suggestive of peripheral and/or central vestibular pathology.[29,57] Peripheral pathology may be related to the presence of microglia in the inner ear,[8] plaque in the eighth cranial nerve's entry zone[74] or a coexisting peripheral pathology such as BPPV.[25] Central pathology may be related to the inflammatory demyelination in the vestibular nuclei,[24] lower pons, upper medulla[75] or

For peer review only

there could be multiple lesions dispersed throughout the brainstem and cerebellar peduncles.[76] Zeigelboim et al. (2008) primarily found peripheral vestibular pathology (83.4%) in their participants with RRMS,[57] while Degirmenci et al. (2010) mainly found central vestibular pathology (83.3%) in their participants with RRMS.[29] These contradictory results might be due to the inclusion of participants with RRMS in both studies, as persons with RRMS may differ in terms of lesion site in the CNS, severity of impairment, developmental stage of disease and frequency of attacks.[57] Given this limited and contradictory evidence, the effects of MS on ENGs are still unknown. Therefore, more literature is needed on different MS subtypes to better understand this. Second, the four included studies that investigated postural control using either static or dynamic posturography found abnormal posturography results in PwMS.[42,43,58,59] To maintain balance, the CNS evaluates the input from the vestibular, visual and somatosensory systems, then weighs the input according to where reliable information is coming from, and MS may affect each of these three systems.[58] Maintaining body balance in PwMS requires increasing the weight on the other two systems if one sensory system input is impaired. Yang and Liu (2020) concluded that PwMS rely more on cues from visual and proprioceptive systems to maintain their balance.[58] Lord et al. (1991) and Doty et al. (2018) also stated that PwMS had difficulty maintaining their balance when visual and proprioceptive feedback were inconsistent or conflicting.[59,77] In addition, a previous study reported that PwMS have slower somatosensory conduction than healthy individuals.[78] Thus, PwMS might receive impaired or delayed proprioceptive feedback resulting in postural instability.[58,78,79] Another possible cause of instability in PwMS is that motor neurons might be affected by demyelination plaques, leading to an impairment in the transmission from the CNS to the motor end units.[58,80] Although, all four included studies found greater instability and impaired balance in PwMS, the evidence regarding the effects of MS on functional balance outcomes is very limited. Further research is required to make a clear

For peer review only

conclusion on this subject. Third, 13 of the 14 included studies that assessed VEMPs reported abnormalities in PwMS. Significant differences between PwMS and controls were reported by eight of the ten studies that compared the two groups.[34,39,52,60,62–65] Both Kavasoğlu et al. (2018) and Cochrane et al. (2021) did not find differences between PwMS and controls.[61,67] However, Kavasoğlu et al. (2018) found VEMP latency delays exceeding 2.5 SD from normative data in 23% of their PwMS;[67] Cochrane et al. (2021) did not report whether any VEMP abnormalities were found in their PwMS.[61] Kavasoğlu et al. (2018) included PwMS that were recently diagnosed (within less than one year), this may be the reason why they did not find differences between PwMS and controls.[67] Cochrane et al. (2021) attributed the lack of difference between PwMS and controls to the fact that their participants had low scores on the disability scale indicating that they were less likely to have lesions affecting VEMP results.[61] However, Gabelić et al. (2013) found differences between PwMS and controls and their participants had similar disability scale scores to Cochrane et al. (2021).[60] The remaining studies that reported percent PwMS that had abnormal VEMPs found various results. For example, Alpini et al. (2004) found abnormalities in 70 %,[33] Versino et al. (2002) found abnormalities in 31.4 %[32] and Koura and Hussein (2018) found abnormalities in 100 % of PwMS who participated in their study.[68] The majority did not report type or duration of MS, or severity of impairment, therefore, it is difficult to speculate why some studies reported higher percentages of VEMP abnormalities in PwMS than others. The most common reported cVEMP abnormalities were prolonged p13 and n23 latencies[32–34,39,60,63–68] and the most common reported oVEMP abnormalities were prolonged p1 and n1 latencies.[39,60] Prolonged latencies may be a result of inflammatory demyelination in the vestibular nuclei and the root entry zone of the eighth cranial nerve[24] leading to reduced neural transmission speed and consequently prolonged latencies. Additionally, Aidar and Suzuki (2005), and Koura and Hussein (2018) noted the absence of waves in 30 % and 40 % of

For peer review only - http://bmjopen.bmj.com/site/about/guidelines.xhtml

PwMS,[64,68] respectively, which may be caused by a lesion in the vestibulospinal tract.[81] Although all studies showed VEMP abnormalities in PwMS, the nature of these abnormalities and their relationships to MS could not be established. This is due to the differences between studies in VEMP stimulus and recording parameters (e.g. presentation levels, stimulus polarity, transducer), resulting in the inability to combine data for a meta-analysis. In addition, type, duration, and severity of MS were not consistently reported. Therefore, there is a clear need for consistency across studies to better understand the effects of MS on the vestibular system by using VEMPs.

Finally, the two studies that reported results on Rotary Chair testing from the same participants found differences that indicate central rather than peripheral vestibular impairments in PwMS compared to controls.[61,69] They suggested that central integration of peripheral input is what is impaired in PwMS.[61,69] However, more studies are needed to confirm these findings.

For peer review only

CONCLUSIONS

This systematic review aimed at understanding the effects of MS on the auditory and vestibular systems. No previous review that investigated this relationship was found. In this systematic review, an effect of MS on the auditory system was consistently found in the results of ABRs, CAEPs, WDS and GIN tests, but not in OAEs or PTAs. Indicating that the brainstem and central auditory systems were more likely to be affected by MS than the peripheral auditory system. Furthermore, an effect of MS on the vestibular system was consistently found in the results of ENG, Dynamic and Static Posturography and VEMPs. This suggests that both the peripheral and central vestibular systems may be involved in the degenerative process of MS. However, quantitative analyses could not be performed in this systematic review due to inconsistencies across studies in reporting of results, in test procedures and in test stimulus and recording parameters. Therefore, although abnormal results in PwMS on both auditory and vestibular

For peer review only - http://bmjopen.bmj.com/site/about/guidelines.xhtml

tests were identified, further studies are necessary to quantify this effect and obtain more robust evidence on the effects of MS on the auditory and vestibular systems. Future studies should use consistent test parameters and account for confounding factors such as type, duration and severity of MS.

Figure Legend:

Figure 1. Flow diagram of systematic literature review

For peer review only

Funding: This research received no specific grant from any funding agency in the public, commercial or not-for-profit sectors. KK was supported by the NIHR Manchester Biomedical Research Centre, UK IS-BRC-1215-20007 and MRC Programme Grant MR/L003589/1.

Competing interest: No competing interests.

Data Sharing: No additional data available.

For peer review only

For peer review only - http://bmjopen.bmj.com/site/about/guidelines.xhtml

REFERENCES

1 Sospedra M, Martin R. Immunology of Multiple Sclerosis. *Annu Rev Immunol* 2005;23:683–747. doi:10.1146/annurev.immunol.23.021704.115707

2 Gold R, Linington C, Lassmann H. Understanding pathogenesis and therapy of multiple sclerosis via animal models: 70 years of merits and culprits in experimental autoimmune encephalomyelitis research. *Brain* 2006;**129**:1953–71. doi:10.1093/brain/awl075

3 Trapp BD, Peterson J, Ransohoff RM, *et al.* Axonal Transection in the Lesions of Multiple Sclerosis. *New Engl J Medicine* 1998;**338**:278–85. doi:10.1056/nejm199801293380502

4 Popescu BFGh, Lucchinetti CF. Pathology of Demyelinating Diseases. *Annu Rev Pathology Mech Dis* 2012;**7**:185–217. doi:10.1146/annurev-pathol-011811-132443

5 Rolak LA. Multiple Sclerosis: It's Not The Disease You Thought It Was. *Clin Medicine Res* 2003;**1**:57–60. doi:10.3121/cmr.1.1.57

6 Coelho A, erani B, Prasher D, *et al.* Auditory Efferent Function Is Affected in Multiple Sclerosis. *Ear Hearing* 2007;**28**:593–604. doi:10.1097/aud.0b013e31812f716e

7 Bogie JFJ, Stinissen P, Hendriks JJA. Macrophage subsets and microglia in multiple sclerosis. *Acta Neuropathol* 2014;**128**:191–213. doi:10.1007/s00401-014-1310-2

8 O'Malley JT, Nadol JB, McKenna MJ. Anti CD163+, Iba1+, and CD68+ Cells in the Adult Human Inner Ear. *Otol Neurotol* 2016;**37**:99–108. doi:10.1097/mao.0000000000000879

9 Di Stadio AD, Ralli M. Inner ear involvement in multiple sclerosis: An underestimated condition? *Mult Scler J* 2018;**24**:1264–5. doi:10.1177/1352458517750010

10 Di Stadio AD, Dipietro L, Ralli M, *et al.* Sudden hearing loss as an early detector of multiple sclerosis: a systematic review. *Eur Rev Med Pharmaco* 2018;**22**:4611–24. doi:10.26355/eurrev_201807_15520

11 Franklin DJ, Coker NJ, Jenkins HA. Sudden Sensorineural Hearing Loss as a Presentation of Multiple Sclerosis. *Archives Otolaryngology Head Neck Surg* 1989;**115**:41–5. doi:10.1001/archotol.1989.01860250043023

12 Drulović B, Ribarić-jankes K, Kostić V, *et al.* Multiple Sclerosis as the Cause of Sudden 'Pontine' Deafness. *Int J Audiol* 1994;**33**:195–201. doi:10.3109/00206099409071880

13 Saberi A, Hatamian HR, Nemati S, *et al.* Hearing statement in multiple sclerosis: a case control study using auditory brainstem responses and otoacoustic emissions. *Acta Medica Iranica* 2012;**50**:679–83.

14 Furst M, Levine RA. Hearing disorders in multiple sclerosis. *Handb Clin Neurology* 2015;**129**:649–65. doi:10.1016/b978-0-444-62630-1.00036-6

For peer review only

15 Atula S, Sinkkonen S, Saat R, *et al.* Association of multiple sclerosis and sudden sensorineural hearing loss. *Multiple Scler J - Exp Transl Clin* 2016;**2**:2055217316652155. doi:10.1177/2055217316652155

16 Dayal VS, Swisher LP. Pure tone thresholds in multiple sclerosis: A further study. *Laryngoscope* 1967;**77**:2169–77. doi:10.1288/00005537-196712000-00007

17 Cohen M, Rudge P. The Effect of Multiple Sclerosis on Pure Tone Thresholds. *Acta Oto-laryngol* 1984;**97**:291–5. doi:10.3109/00016488409130991

18 Lewis MS, Lilly DJ, Hutter MM, *et al.* Audiometric hearing status of individuals with and without multiple sclerosis. *J Rehabilitation Res Dev* 2010;**47**:669–78. doi:10.1682/jrrd.2010.01.0010

19 Doty RL, Tourbier I, Davis S, *et al.* Pure-Tone Auditory Thresholds Are Not Chronically Elevated in Multiple Sclerosis. *Behav Neurosci* 2012;**126**:314–24. doi:10.1037/a0027046

20 Lima TMA, Crato AN, Mancini PC, *et al.* Alterations in early auditory evoked potentials in multiple sclerosis patients. *Braz J Otorhinolar* 2009;**75**:177–81. doi:10.1016/s1808-8694(15)30775-8

21 Di Mauro RD, Di Girolamo SD, Ralli M, *et al.* Subclinical cochlear dysfunction in newly diagnosed relapsing-remitting multiple sclerosis. *Mult Scler Relat Dis* 2019;**33**:55–60. doi:10.1016/j.msard.2019.05.020

22 Valadbeigi A, Weisi F, Rohbakhsh N, *et al.* Central auditory processing and word discrimination in patients with multiple sclerosis. *Eur Arch Oto-rhino-l* 2014;**271**:2891–6. doi:10.1007/s00405-013-2776-6

23 Elbeltagy R, Gad N, Ismail M. Central auditory function in multiple sclerosis patients. *Indian J Otology* 2019;**25**:90–6. doi:10.4103/indianjotol.indianjotol_80_18

24 Brandt T, Dieterich M. Preliminary Classification of Vestibular Brain-Stem Disorders. In: L.R. C, H.C. H, eds. *Brain-Stem Localization and Function*. Berlin, Heidelberg: : Springer 1993. 79–91. doi:10.1007/978-3-642-78172-8_9

25 Alpini D, Caputo D, Pugnetti L, *et al.* Vertigo and multiple sclerosis: aspects of differential diagnosis. *Neurol Sci* 2001;**22**:S84–7. doi:10.1007/s100720100041

26 Noffsinger D, Olsen WO, Carhart R, *et al.* Auditory and vestibular aberrations in multiple sclerosis. *Acta Oto-laryngologica Suppl* 1972;**303**:1–63.

27 Grénman R. Involvement of the audiovestibular system in multiple sclerosis. An otoneurologic and audiologic study. *Acta Oto-laryngologica Suppl* 1985;**420**:1–95.

28 Cipparrone L, Fratiglioni L, Siracusa G, *et al.* Electronystagmography in the diagnosis of multiple sclerosis. *Acta Neurol Scand* 1989;**80**:193–200. doi:10.1111/j.1600-0404.1989.tb03862.x

For peer review only

29 Degirmenci E, Bir LS, Ardic FN. Clinical and electronystagmographical evaluation of vestibular symptoms in relapsing remitting multiple sclerosis. *Neurol Res* 2010;**32**:986–91. doi:10.1179/016164110x12681290831405

30 Shimizu K, Murofushi T, Sakurai M, *et al.* Vestibular evoked myogenic potentials in multiple sclerosis. *J Neurology Neurosurg Psychiatry* 2000;**69**:276–7. doi:10.1136/jnnp.69.2.276

31 Murofushi T, Shimizu K, Takegoshi H, *et al.* Diagnostic Value of Prolonged Latencies in the Vestibular Evoked Myogenic Potential. *Archives Otolaryngology Head Neck Surg* 2001;**127**:1069–72. doi:10.1001/archotol.127.9.1069

32 Versino M, Colnaghi S, Callieco R, *et al.* Vestibular evoked myogenic potentials in multiple sclerosis patients. *Clin Neurophysiol* 2002;**113**:1464–9. doi:10.1016/s1388-2457(02)00155-4

33 Alpini D, Pugnetti L, Caputo D, *et al.* Vestibular evoked myogenic potentials in multiple sclerosis: clinical and imaging correlations. *Mult Scler* 2004;**10**:316–21. doi:10.1191/1352458504ms1041oa

34 Eleftheriadou A, Deftereos SN, Zarikas V, *et al.* The diagnostic value of earlier and later components of Vestibular Evoked Myogenic Potentials (VEMP) in Multiple Sclerosis. *J Vestib Res* 2009;**19**:59–66. doi:10.3233/ves-2009-0342

35 Gazioglu S, Boz C. Ocular and cervical vestibular evoked myogenic potentials in multiple sclerosis patients. *Clin Neurophysiol* 2012;**123**:1872–9. doi:10.1016/j.clinph.2012.01.022

36 Güven H, Bayır Ö, Aytaç E, *et al.* Vestibular-evoked myogenic potentials, clinical evaluation, and imaging findings in multiple sclerosis. *Neurol Sci* 2014;**35**:221–6. doi:10.1007/s10072-013-1483-9

37 Crnošija L, Skorić MK, Gabelić T, *et al.* Correlation of the VEMP score, ambulation and upper extremity function in clinically isolated syndrome. *J Neurol Sci* 2015;**359**:197–201. doi:10.1016/j.jns.2015.10.049

38 Gabelić T, Skorić MK, Adamec I, *et al.* The vestibular evoked myogenic potentials (VEMP) score: a promising tool for evaluation of brainstem involvement in multiple sclerosis. *Eur J Neurol* 2015;**22**:261-e21. doi:10.1111/ene.12557

39 Parsa MS, Mohammadkhani G, Hajabolhassani F, *et al.* Cervical and ocular vestibular evoked myogenic potentials in multiple sclerosis participants. *Medical J Islamic Repub Iran* 2015;**29**:164.

40 Crnošija L, Skorić MK, Gabelić T, *et al.* Vestibular evoked myogenic potentials and MRI in early multiple sclerosis: Validation of the VEMP score. *J Neurol Sci* 2017;**372**:28–32. doi:10.1016/j.jns.2016.11.028

41 Di Stadio AD, Dipietro L, Ralli M, *et al.* The role of vestibular evoked myogenic potentials in multiple sclerosis-related vertigo. A systematic review of the literature. *Mult Scler Relat Dis* 2019;**28**:159–64. doi:10.1016/j.msard.2018.12.031

For peer review only - http://bmjopen.bmj.com/site/about/guidelines.xhtml

42 Inojosa H, Schriefer D, Klöditz A, *et al.* Balance Testing in Multiple Sclerosis—Improving Neurological Assessment With Static Posturography? *Front Neurol* 2020;**11**:135. doi:10.3389/fneur.2020.00135

43 Inojosa H, Schriefer D, Trentzsch K, *et al.* Visual Feedback and Postural Control in Multiple Sclerosis. *J Clin Medicine* 2020;**9**:1291. doi:10.3390/jcm9051291

44 Prosperini L, Petsas N, Raz E, *et al.* Balance deficit with opened or closed eyes reveals involvement of different structures of the central nervous system in multiple sclerosis. *Mult Scler J* 2014;**20**:81–90. doi:10.1177/1352458513490546

45 Page MJ, McKenzie JE, Bossuyt PM, *et al.* The PRISMA 2020 statement: an updated guideline for reporting systematic reviews. *Bmj* 2021;**372**:n71. doi:10.1136/bmj.n71

46 Kim SY, Park JE, Lee YJ, *et al.* Testing a tool for assessing the risk of bias for nonrandomized studies showed moderate reliability and promising validity. *J Clin Epidemiol* 2013;**66**:408–14. doi:10.1016/j.jclinepi.2012.09.016

47 Wells G, Shea B, O'Connell D, *et al.* The Newcastle-Ottawa Scale (NOS) for assessing the quality of nonrandomised studies in meta-analyses. Ottawa: Ottawa Hospital Research Institute. http://www.ohri.ca/programs/clinical_epidemiology/oxford.asp (accessed 2 Jul 2020).

48 Moskalewicz A, Oremus M. No clear choice between Newcastle–Ottawa Scale and Appraisal Tool for Cross-Sectional Studies to assess methodological quality in cross-sectional studies of health-related quality of life and breast cancer. *J Clin Epidemiol* 2020;**120**:94–103. doi:10.1016/j.jclinepi.2019.12.013

49 Kaytancı E, Ozdamar OI, Acar GO, *et al.* Evaluation of transiently evoked otoacoustic emissions and auditory brainstem responses in patients with multiple sclerosis. *Ear Nose Throat J* 2016;**95**:E12–7.

50 Srinivasan VS, Krishna R, Munirathinam BR. Effectiveness of Brainstem Auditory Evoked Potentials Scoring in Evaluating Brainstem Dysfunction and Disability Among Individuals With Multiple Sclerosis. *Am J Audiol* 2021;**30**:255–65. doi:10.1044/2020_aja-20-00155

51 Matas CG, Matas SL de A, Oliveira CRS de, *et al.* Auditory evoked potentials and multiple sclerosis. *Arq Neuro-psiquiat* 2010;**68**:528–34. doi:10.1590/s0004-282x2010000400010

52 Delphi M, Sayaf M, Taheri F, *et al.* Audio and vestibular analyses in patient with multiple sclerosis: a case–control study. *Hear Balance Commun* 2021;**19**:1–6. doi:10.1080/21695717.2021.1933318

53 Japaridze G, Shakarishvili R, Kevanishvili Z. Auditory brainstem, middle-latency, and slow cortical responses in multiple sclerosis. *Acta Neurol Scand* 2002;**106**:47–53. doi:10.1034/j.1600-0404.2002.01226.x

For peer review only

54 Burina A, Sinanović O, Smajlović D, *et al.* Some Aspects of Balance Disorder in Patients with Multiple Sclerosis. *Bosnian J Basic Med* 2008;**8**:80–5. doi:10.17305/bjbms.2008.3003

55 Pokryszko-Dragan A, Bilinska M, Gruszka E, *et al.* Assessment of visual and auditory evoked potentials in multiple sclerosis patients with and without fatigue. *Neurol Sci* 2015;**36**:235–42. doi:10.1007/s10072-014-1953-8

56 Rishiq D, Harkrider A, Springer C, *et al.* Click-evoked and speech-evoked auditory brainstem responses from individuals with multiple sclerosis. *Neurosci Lett* 2021;**740**:135460. doi:10.1016/j.neulet.2020.135460

57 Zeigelboim BS, Arruda WO, Mangabeira-Albernaz PL, *et al.* Vestibular findings in relapsing, remitting multiple sclerosis: a study of thirty patients. *Int Tinnitus J* 2008;**14**:139–45.

58 Yang F, Liu X. Relative importance of vision and proprioception in maintaining standing balance in people with multiple sclerosis. *Mult Scler Relat Dis* 2020;**39**:101901. doi:10.1016/j.msard.2019.101901

59 Doty RL, MacGillivray MR, Talab H, *et al.* Balance in multiple sclerosis: relationship to central brain regions. *Exp Brain Res* 2018;**236**:2739–50. doi:10.1007/s00221-018-5332-1

60 Gabelić T, Krbot M, Šefer AB, *et al.* Ocular and Cervical Vestibular Evoked Myogenic Potentials in Patients With Multiple Sclerosis. *J Clin Neurophysiol* 2013;**30**:86–91. doi:10.1097/wnp.0b013e31827eda0c

61 Cochrane GD, Christy JB, Motl RW. Comprehensive Clinical Assessment of Vestibular Function in Multiple Sclerosis. *J Neurol Phys Ther* 2021;**45**:228–34. doi:10.1097/npt.0000000000000358

62 Elmoazen D, Kozou H, Gawies M, *et al.* Assessment of Cervical and Ocular Vestibular Evoked Myogenic Potentials in Multiple Sclerosis PatientsS. *Egyptian Journal of Ear, Nose, Throat and Allied Sciences* 2021;**22**:1–9. doi:10.21608/ejentas.2020.44108.1262

63 Sartucci F, Logi F. Vestibular-evoked myogenic potentials: A method to assess vestibulo-spinal conduction in multiple sclerosis patients. *Brain Res Bull* 2002;**59**:59–63. doi:10.1016/s0361-9230(02)00842-0

64 Aidar RC, Suzuki FA. Vestibular evoked myogenic potential: new perspectives in multiple sclerosis. *Braz J Otorhinolar* 2005;**71**:48–54. doi:10.1016/s1808-8694(15)31284-2

65 Patkó T, Simó M, Arányi Z. Vestibular click-evoked myogenic potentials: sensitivity and factors determining abnormality in patients with multiple sclerosis. *Mult Scler J* 2007;**13**:193–8. doi:10.1177/1352458506070940

66 Harirchian MH, Karimi N, Nafisi S, *et al.* Vestibular evoked myogenic potential for diagnoses of multiple sclerosis: is it beneficial? *Medicinski Glasnik Official Publ Medical Assoc Zenica-doboj Canton Bosnia Herzegovina* 2013;**10**:321–6.

For peer review only

For peer review only - http://bmjopen.bmj.com/site/about/guidelines.xhtml

67 Kavasoğlu G, Gökçay F, Yüceyar N, *et al.* Cervical vestibular-evoked myogenic potentials in patients with multiple sclerosis: sensitive in detecting brainstem involvement? *Neurol Sci* 2018;**39**:365–71. doi:10.1007/s10072-017-3215-z

68 Koura R, Hussein M. Vestibular-evoked myogenic potential: an easy neurophysiological tool for evaluating brain stem involvement in multiple sclerosis. *Egypt J Otolaryngology* 2018;**34**:144–8. doi:10.4103/ejo.ejo_73_17

69 Cochrane GD, Christy JB, Sandroff BM, *et al.* Cognitive and Central Vestibular Functions Correlate in People With Multiple Sclerosis. *Neurorehab Neural Re* 2021;**35**:1030–8. doi:10.1177/15459683211046268

70 Veuillet E, Khalfa S, Collet L. Clinical relevance of medial efferent auditory pathways. *Scand Audiology Suppl* 1999;**51**:53–62.

71 Protti-Patterson E, Young ML. The use of subjective and objective audiologic test procedures in the diagnosis of multiple sclerosis. *Otolaryng Clin N Am* 1985;**18**:241–55.

72 Magnano I, Aiello I, Piras MR. Cognitive impairment and neurophysiological correlates in MS. *J Neurol Sci* 2006;**245**:117–22. doi:10.1016/j.jns.2005.08.027

73 Musiek FE, Shinn JB, Jirsa R, *et al.* GIN (Gaps-In-Noise) Test Performance in Subjects with Confirmed Central Auditory Nervous System Involvement. *Ear Hearing* 2005;**26**:608–18. doi:10.1097/01.aud.0000188069.80699.41

74 Minagar A, Sheremata WA, Tusa RJ. Perverted head-shaking nystagmus: A possible mechanism. *Neurology* 2001;**57**:887–9. doi:10.1212/wnl.57.5.887

75 Schumacher GA. Demyelinating Diseases as a Cause for Vertigo. *Archiv Otolaryngol* 1967;**85**:537–8. doi:10.1001/archotol.1967.00760040539012

76 Pula JH, Newman-Toker DE, Kattah JC. Multiple sclerosis as a cause of the acute vestibular syndrome. *J Neurol* 2013;**260**:1649–54. doi:10.1007/s00415-013-6850-1

77 Lord SR, Clark RD, Webster IW. Postural Stability and Associated Physiological Factors in a Population of Aged Persons. *J Gerontology* 1991;**46**:M69–76. doi:10.1093/geronj/46.3.m69

78 Cameron MH, Lord S. Postural Control in Multiple Sclerosis: Implications for Fall Prevention. *Curr Neurol Neurosci* 2010;**10**:407–12. doi:10.1007/s11910-010-0128-0

79 Cameron MH, Horak FB, Herndon RR, *et al.* Imbalance in multiple sclerosis: A result of slowed spinal somatosensory conduction. *Somatosens Mot Res* 2008;**25**:113–22. doi:10.1080/08990220802131127

80 Cattaneo D, Jonsdottir J. Sensory impairments in quiet standing in subjects with multiple sclerosis. *Mult Scler* 2009;**15**:59–67. doi:10.1177/1352458508096874

For peer review only

729 81 Colebatch JG, Halmagyi GM, Skuse NF. Myogenic potentials generated by a click-evoked
730 vestibulocollic reflex. *J Neurology Neurosurg Psychiatry* 1994;**57**:190–7.
731 doi:10.1136/jnnp.57.2.190

For peer review only

For peer review only - http://bmjopen.bmj.com/site/about/guidelines.xhtml

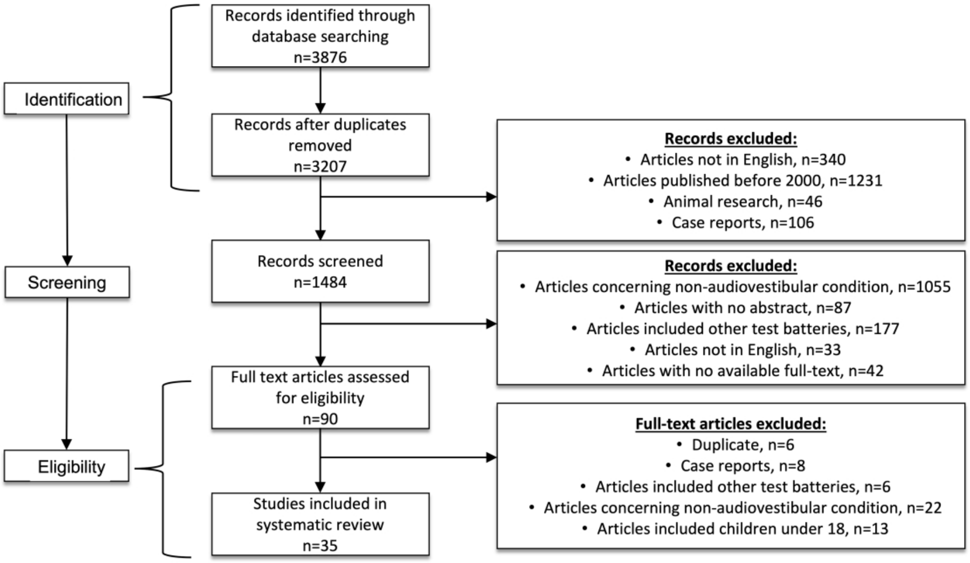

Figure 1. Flow diagram of systematic literature review

Supplement

Section I: Assessment of Bias

Table S1: *Assessment of Bias for case-control studies according to RoBANs*

Year	Author	Selection of participants	Confounding Variables	Measurement of Exposure	Blinding of the Outcome of the Assessment	Incomplete Outcome of Data	Selective Outcome Reporting
2002	Japaridze et al.[1]	?	+	+	-	+	+
2002	Sartucci and Logi[2]	+	+	+	-	+	+
2004	Alpini et al.[3]	-	?	+	-	+	+
2005	Aidar and Suzuki[4]	+	+	+	-	+	-
2007	Patkó et al.[5]	?	-	+	-	+	+
2008	Zeigelboim et al.[6]	?	+	+	-	+	+
2009	Lima et al.[7]	?	?	+	-	+	+
2010	Lewis et al.[8]	+	+	+	-	+	+
2010	Matas et al.[9]	+	+	+	-	+	+
2012	Doty et al.[10]	-	+	+	-	+	+
2012	Saberi et al.[11]	+	+	+	-	+	+
2013	Gabelić et al.[12]	+	+	+	-	+	+
2013	Harirchian et al.[13]	+	+	+	-	+	+
2014	Valadbeigi et al.[14]	+	+	+	-	+	+
2015	Pokryszko-Dragan et al.[15]	+	+	+	-	+	+
2018	Doty et al.[16]	-	+	+	-	+	+
2018	Kavasoğlu et al.[17]	+	?	+	-	+	+
2018	Koura et al.[18]	+	+	+	-	+	+
2019	Elbeltagy et al.[19]	-	+	+	-	+	+
2021	Rishiq et al.[20]	?	+	+	-	+	+
2021	Srinivasan et al.[21]	+	+	+	-	+	+
2021	Elmoazen et al.[22]	+	+	+	-	+	+

Green "+" = low risk of bias, red "-" = high risk of bias, yellow "?" = unclear risk of bias.

Table S2: *Risk of bias assessment of cohort studies by the Newcastle-Ottawa Quality Assessment form for cohort studies.*

Year	Author	Representativeness of exposed cohort	Selection of non-exposed cohort	Ascertainment of exposure	Outcome of interest not present at the beginning	Comparability of cohorts/confounders	Assessment of the outcome	Follow-up Length	Adequacy of follow-up of cohorts	Study quality
2002	Versino et al.[23]	*	*n/a*	*	*	*n/a*	-	*n/a*	*	?
2008	Burina et al.[24]	-	*n/a*	*	*n/a*	-	-	-	-	?
2009	Eleftheriadou et al.[25]	*	-	*	*	*	-	*	*	Poor
2010	Degirmenci et al.[26]	*	-	*	*	*	*	*	*	Good
2016	Kaytancı et al.[27]	-	-	*	-	*	-	*	*	Poor
2019	Di Mauro et al.[28]	*	-	*	*	*	-	*	*	Good

'' = low risk of bias, '-' = high risk of bias, "n/a" = the criteria does not apply to the paper, "?" = unknown quality of the paper due to the presence of "n/a" criteria.*

Table S3: *Risk of bias assessment of cross-sectional studies by the Newcastle-Ottawa Quality Assessment form which is adapted for cross-sectional studies.*

Year	Author	Representativeness of the sample	Sample Size	Non-respondents	Ascertainment of exposure (risk factor)	Comparability of groups/confounders	Assessment of the outcome	Statistical test	Study quality
2015	Parsa et al.[29]	-	-	-	*	*	-	*	Poor
2020	Yang and Liu[30]	*	-	*	*	*	-	*	Good
2020	Inojosa et al.[31]	*	-	*	*	*	-	*	Good
2020	Inojosa et al.[32]	*	-	*	*	*	-	*	Good
2021	Delphi et al.[33]	-	-	*	*	*	-	*	Good
2021	Cochrane et al.[34]	*	-	*	*	*	-	*	Good
2021	Cochrane et al.[35]	*	-	*	*	*	-	*	Good

'' = low risk of bias, '-' = high risk of bias.*

Section II: Multiple Sclerosis and Auditory Function
Extracted Pure Tone Audiometry Thresholds and Auditory Brainstem Response Results

Table S4: Pure Tone Audiometry Thresholds

Author (Year)	Group	Test Ear	PTA Threshold Per Frequency (Hz)														
			125		250		500		1000		2000		4000		8000	
			$\bar{x}$	SD	$\bar{x}$	SD	$\bar{x}$	SD	$\bar{x}$	SD	$\bar{x}$	SD	$\bar{x}$	SD	$\bar{x}$	SD
Doty et al. (2012)[10]	Male PwMS	Average Across Ears	Not Tested		15.87	5.39	14.05	5.94	12.52	7.43	12.79	8.97	20.03	11.71	17.02	17.26
	Male Controls				14.35	8.24	15.90	8.68	15.53	8.58	17.74	8.93	22.77	16.08	20.23	22.49
	Female PwMS				12.85	6.88	12.49	6.50	12.05	6.59	14.62	7.19	15.65	8.97	13.28	12.38
	Female Controls				11.28	7.77	12.52	7.60	10.45	6.82	11.62	8.17	13.92	8.79	12.65	12.60
Di Mauro et al. (2019)[28]	PwMS	Right	10.30	1.60	10.10	0.08	10.10	0.08	10.40	1.10	11.10	1.70	10.80	1.80	13.00	2.60
	Controls	Right	10.90	1.40	10.80	1.60	11.00	1.90	10.80	2.30	11.00	1.20	11.90	2.40	13.90	2.40
	PwMS	Left	10.30	1.70	10.90	1.20	10.40	1.90	10.40	1.30	10.80	1.10	11.40	1.70	12.50	2.20
	Controls	Left	10.90	1.40	10.80	1.60	11.00	1.30	10.90	2.10	11.10	1.90	11.90	2.80	13.90	2.40
Srinivasan et al. (2021)[21]	PwMS	Average Across Ears	Not Tested		12.22	6.40	13.11	5.92	13.61	5.90	12.55	7.19	13.66	8.06	16.16	9.08
	Controls				8.94	5.05	9.50	4.17	9.88	3.82	9.50	5.45	10.88	5.83	12.05	5.89

Abbreviations:
PwMS = Persons with Multiple Sclerosis, PTA = Pure Tone Audiometry, $\bar{x}$ = Mean, SD = Standard Deviation

For peer review only

Table S5: Auditory Brainstem Response Parameters and Results

Author (Year)	ABR Parameters							Peak Latency (ms)						Interpeak Latencies (ms)						Peak Amplitude (μV)			
	Click Polarity - Transducer	PR	PL	Ave.	BP Filter	Group	Test Ear	I		III		V		I-III		III-V		I-V		I		V	
								x̄	SD	x̄	SD	x̄	SD	x̄	SD	x̄	SD	x̄	SD	x̄	SD	x̄	SD
Lima et al. (2009)[7]	Rarefaction - TDH 39 Headphone	13.10	60 dB SL*	1024	100-3000	PwMS	Right	1.50	0.16	3.63	0.30	5.56	0.40	2.13	0.22	1.93	0.31	4.06	0.33	?	?	?	?
							Left	1.53	0.20	3.63	0.30	5.64	0.43	2.10	0.31	2.01	0.29	4.11	0.39	?	?	?	?
Matas et al. (2010)[9]	Rarefaction - TDH 39 Headphone	19.90	80 dB nHL	2000	100-3000	PwMS	Average Across Ears	1.53	0.13	3.72	0.31	5.78	0.37	2.21	0.26	2.03	0.27	4.26	0.35	?	?	?	?
						Controls		1.53	0.07	3.57	0.09	5.50	0.45	2.04	0.06	1.98	0.08	4.03	0.06	?	?	?	?
Pokryszko-Dragan et al. (2015)[15]	? - Earphone unspecified	20.30	65 dB SL*	200	150-3000	PwMS	Right	1.72	0.19	3.88	0.33	5.81	0.81	2.18	0.19	2.00	0.30	4.19	0.39	0.29	0.18	0.37	0.20
						Controls		1.70	0.14	3.85	0.16	5.73	0.23	2.14	0.12	1.87	0.18	4.02	0.20	0.30	0.10	0.46	0.19
						PwMS	Left	1.68	0.12	3.87	0.17	5.77	0.73	2.19	0.16	1.96	0.30	4.15	0.34	0.32	0.14	0.40	0.24
						Controls		1.69	0.13	3.83	0.12	5.70	0.21	2.13	0.14	1.87	0.17	4.00	0.19	0.30	0.14	0.44	0.16
Kaytancı et al. (2016)[27]	? - TDH 39 Headphone	13.00	70 dB nHL	1500	2000-4000	PwMS	Right	2.03	0.27	4.36	0.49	6.26	0.35	2.33	0.49	1.90	0.28	4.23	0.42	?	?	?	?
						Controls		1.45	0.30	3.52	0.31	5.38	0.53	2.07	0.44	1.85	0.48	3.92	0.53	?	?	?	?
						PwMS	Left	1.86	0.24	4.41	0.39	6.25	0.43	2.54	0.34	1.84	0.20	4.38	0.43	?	?	?	?
						Controls		1.47	0.24	3.40	0.43	5.39	0.46	1.92	0.48	1.99	0.53	3.91	0.47	?	?	?	?
						PwMS	IA Diff.	?	?	?	?	0.40	0.46	0.35	0.24	0.49	0.46	0.40	0.48	?	?	?	?
						Controls		?	?	?	?	0.15	0.12	0.34	0.35	0.27	0.30	0.19	0.16	?	?	?	?
Delphi et al. (2021)[33]	Rarefaction - ?	9.00	70 dB nHL	?	30-3000	PwMS	Right	1.89	0.11	4.25	0.14	6.32	0.13	2.30	0.11	2.21	0.09	4.48	0.13	?	?	?	?
						Controls		1.87	0.14	4.01	0.13	5.79	0.14	2.17	0.09	1.99	0.13	4.12	0.11	?	?	?	?
						PwMS	Left	1.80	0.15	4.30	0.15	6.19	0.14	2.29	0.08	2.19	0.14	4.51	0.11	?	?	?	?
						Controls		1.79	0.11	3.96	0.13	5.87	0.15	2.15	0.14	1.96	0.08	4.09	0.14	?	?	?	?
Srinivasan et al. (2021)[21]	Rarefaction – Insert earphones	21.10	80 dB nHL	1500	100-3000	PwMS	Average Across Ears	1.44	0.18	3.61	0.40	5.63	0.73	2.17	0.38	2.03	0.50	4.19	0.70	?	?	?	?
						Controls		1.43	0.16	3.48	0.19	5.30	0.24	2.04	0.17	1.82	0.18	3.86	0.21	?	?	?	?
		80.10				PwMS	Average Across Ears	?	?	?	?	6.12	0.72	?	?	?	?	?	?	?	?	?	?
						Controls		?	?	?	?	5.76	0.27	?	?	?	?	?	?	?	?	?	?

Abbreviations:
PR = Presentation Rate, PL = Presentation Level, Ave. = Number of Averages, BP = Band Pass, , x̄ = Mean, SD = Standard Deviation, dB SL = Decibels Sensation Level, dB nHL = Decibels normal Hearing Level, PwMS = Persons with Multiple Sclerosis, IA Diff. = Inter-aural Difference, ? = not reported.
* dB SL reference to hearing thresholds (did not specify hearing thresholds to click or to pure tones)

For peer review only - http://bmjopen.bmj.com/site/about/guidelines.xhtml

Section III: Multiple Sclerosis and Vestibular Function
Extracted Vestibular Evoked Myogenic Potentials Results

Table S6: Ocular Vestibular Evoked Myogenic Potentials Parameters and Results

Author (Year)	Stimulus (polarity) - Trasducer	PR (Hz)	PL	Ave.	BP Filter (Hz)	Group	Side	Peak Latency (ms) n1 x̄	n1 SD	p1 x̄	p1 SD	Amplitude (µV) n1-p1 x̄	n1-p1 SD
Gabelić et al. (2013)[12]	Click (?) - Headphone	1	130 dB SPL	100	5-1000	PwMS	Average Across sides	10.30	?	14.90	?	13.10	?
						Controls		9.5	?	14	?	13.3	?
Parsa et al. (2015)[29]	500 Hz Tone Burst (?) - Insert earphones	?	95 db nHL	?	?	PwMS with infratentorial plaque	Right	15.67	1.32	20.59	1.65	6.61	0.88
						PwMS without infratentorial plaque		12.21	1.00	16.90	1.32	6.61	0.60
						Controls		10.2	0.48	15.1	0.87	12.77	4.12
						PwMS with infratentorial plaque	Left	15.74	1.66	21.40	2.88	6.48	0.68
						PwMS without infratentorial plaque		11.78	0.89	16.92	0.82	6.82	0.75
						Controls		10.3	0.51	14.9	0.84	12.6	4.04
Elmoazen et al. (2021)[22]	500 Hz Tone Burst (condensation) -Supra aural headphones	5	95 dB nHL	150	1-1000	PwMS with Brainstem lesions	Average Across sides	11.43	1.42	16.86	1.79	3.59	3.82
						PwMS without Brainstem lesions		10.58	2.50	15.81	1.42	3.22	2.00
						Controls		10.09	0.92	14.71	1.20	2.76	1.28
Cochrane et al. (2021)[34]	Unspecified Tone Burst (?) – earphone unspecified	?	?	100	?	PwMS	?	8.50	1.70	12.90	2.20	5.20	3.50
						Controls		8.40	1.50	12.30	2.10	4.70	2.50

Abbreviations:
PR = Presentation Rate, PL = Presentation Level, Ave. = Number of Averages, BP = Band Pass, , x̄ = Mean, SD = Standard Deviation, dB SPL = Decibels Sound Pressure Level, dB nHL = Decibels normal Hearing Level, PwMS = Persons with Multiple Sclerosis, ? = not reported.

Table S7: Cervical Vestibular Evoked Myogenic Potentials Parameters and Results

Author (Year)	Stimulus (polarity) - Trasducer	PR (Hz)	PL	Ave.	BP Filter (Hz)	Group	Side	Peak Latency (ms)				Amplitude (µV)	
								p13		n23		p13-n23	
								x̄	SD	x̄	SD	x̄	SD
Sartucci and Logi (2002)[2]	Click (Rarefaction) - Headphone	3	140 dB SPL	256	10-1500	PwMS	Right	15.30	3.10	22.50	2.30	63.00	65.50
						Controls		12.5	0.8	21.3	1.5	186.4	82.8
						PwMS	Left	14.00	2.30	20.80	2.30	73.10	58.80
						Controls		12.3	0.9	20.8	1.3	141.1	85.8
Alpini et al. (2004)[3]	Click (Rarefaction) - Headphone	5	95 dB nHL + 70 dB SPL contralateral Masking	400	?	PwMS	Right	14.40	?	22.40	?	?	?
							Left	14.90	?	22.50	?	?	?
Aidar and Suzuki (2005)[4]	Click (Rarefaction) - Headphone	2	95 dB HL	200	20-1000	PwMS	Right	13.40	2.87	18.64	2 .67	12 .17	6 .50
						Controls		11 .12	1.64	15.15	2.51	7.54	5.95
						PwMS	Left	13.49	1 .77	17.84	1.27	13.30	3 .63
						Controls		10.76	1.59	14.86	2.85	7.23	4.76
Patkó et al. (2007)[5]	Click (Rarefaction) - Headphone	5	133 dB SPL	250	10-1600	PwMS	Right	14.40	1.50	21.30	3.20	11.61	3.63
						Controls		12.7	1.5	21.3	3.7	16.02	5.34
						PwMS	Left	14.8	1.5	21.2	3.2	11.07	2.87
						Controls		12.9	1.9	20.7	2.8	13.85	4.3
Eleftheriadou et al. (2009)[25]	Click (Rarefaction) - Headphone	5	105 dB HL	250	5-1500	PwMS with brainstem leasions	Average acrross sides	15.48	1.40	23.94	1.51	?	?
						PwMS without brainstem leasions		14.56	1.20	23.25	1.42	?	?
						Controls		12.67	1.1	21.66	1.11	?	?
Gabelić et al. (2013)[12]	Click (?) - Headphone	1	130 dB SPL	100	5-1000	PwMS	Average across sides	15.80	?	24.90	?	1.90	?
						Controls		14.6	?	22.1	?	1.6	?
Harirchian et al. (2013)[13]	Click (Rarefaction) - Headphone	2	95 db nHL + 40 dB nHL contralateral Masking	200	10-1000	PwMS	Right	22.20	9.30	30.02	11.20	?	?
						Controls		12.9	1.4	21.3	1.6	?	?
						PwMS	Left	20.20	7.05	30.71	16.20	?	?
						Controls		13.1	1.9	20.6	1.9	?	?
						PwMS	IA Diff.	2.05	8.80	0.70	10.90	?	?
						Controls		0.17	2.2	0.4	2.5	?	?

For peer review only - http://bmjopen.bmj.com/site/about/guidelines.xhtml

Table S7 Continued:

Author (Year)	Stimulus (polarity) - Trasducer	PR (Hz)	PL	Ave.	BP Filter (Hz)	Group	Side	Peak Latency (ms) p13 x̄	p13 SD	n23 x̄	n23 SD	Amplitude (µV) p13-n23 x̄	p13-n23 SD
Parsa et al. (2015)[29]	500 Hz Tone Burst (?) -Insert earphones	?	95 db nHL	?	?	PwMS with infratentorial plaque	Right	18.75	1.81	28.15	1.43	118.82	29.97
						PwMS without infratentorial plaque		17.44	1.03	26.89	1.87	135.00	20.31
						Controls		15.25	0.9	24.55	1.07	180	44.97
						PwMS with infratentorial plaque	Left	19.11	1.72	28.88	2.27	119.65	26.02
						PwMS without infratentorial plaque		17.21	1.05	26.87	1.36	133.78	22.89
						Controls		15.31	0.93	24.48	1.18	175	57.59
Kavasoğlu et al. (2017)[17]	Click (Rarefaction) - Headphone	5	100 dB nHL	100	10-1000	PwMS	Right	12.90	2.10	21.20	1.78	7.10	2.80
						Controls		12	0.9	20.6	1.95	7.7	3.1
						PwMS	Left	12.90	2.20	21.40	2.10	7.00	2.50
						Controls		12.03	0.96	20.76	1.63	7.6	3
Koura and Hussein (2018)[18]	500 Hz Tone Burst (Rarefaction) -TDH 39 Headphones	5.1	95 db HL	200	?	PwMS	Right	17.14	0.71	26.82	0.84	41.88	1. 71
						Controls		13.83	1.145	20.76	3.43	61.18	34.64
						PwMS	Left	17.20	0.56	27.17	0.75	54.95	28.27
						Controls		14.43	0.702	22.3	1.725	65.74	18.51
Delphi et al. (2021)[33]	500 Hz Tone Burst (?) -?	5.1	95 dB nHL	?	10-1200	PwMS	Right	18.20	1.93	27.37	2.45	121.34	29.05
						Controls		14.96	1.02	24.21	2.10	169.21	48.76
						PwMS	Left	18.95	2.01	27.21	2.80	-	-
						Controls		15.02	1.10	24.12	2.30	-	-
Elmoazen et al. (2021)[22]	500 Hz Tone Burst (condensation) -Supra aural headphones	5	95 dB nHL	150	1-1000	PwMS with Brainstem lesions	Average across sides	16.49	1.49	25.96	1.93	66.25	52.42
						PwMS without Brainstem lesions		15.21	1.57	23.97	2.06	93.12	78.01
						Controls		14.05	0.94	23.36	2.14	144.42	122.73
Cochrane et al. (2021)[34]	Unspecified Tone Burst (?) – earphone unspecified	?	?	?	?	PwMS	?	13.70	1.30	21.10	2.60	12.70	7.60
						Controls		13.30	0.50	20.60	1.80	16.50	7.10

Abbreviations:
PR = Presentation Rate, PL = Presentation Level, Ave. = Number of Averages, BP = Band Pass, , x̄ = Mean, SD = Standard Deviation, dB SPL = Decibels Sound Pressure Level, dB HL = Decibels Heraing Level, dB nHL = Decibels normal Hearing Level, PwMS = Persons with Multiple Sclerosis, IA Diff. = Inter-aural Difference, ? = not reported. Cells Highlighted in Yellow indicate that "normalized amplitudes" were reported as opposed to absolute amplitudes reported by other studies. Cells Highlighted in Green indicate results were reported without specifying side or if they are averaged across sides.

For peer review only - http://bmjopen.bmj.com/site/about/guidelines.xhtml

REFERENCES

1 Japaridze G, Shakarishvili R, Kevanishvili Z. Auditory brainstem, middle-latency, and slow cortical responses in multiple sclerosis. *Acta Neurol Scand* 2002;**106**:47–53. doi:10.1034/j.1600-0404.2002.01226.x

2 Sartucci F, Logi F. Vestibular-evoked myogenic potentials: A method to assess vestibulo-spinal conduction in multiple sclerosis patients. *Brain Res Bull* 2002;**59**:59–63. doi:10.1016/s0361-9230(02)00842-0

3 Alpini D, Pugnetti L, Caputo D, *et al.* Vestibular evoked myogenic potentials in multiple sclerosis: clinical and imaging correlations. *Mult Scler* 2004;**10**:316–21. doi:10.1191/1352458504ms1041oa

4 Aidar RC, Suzuki FA. Vestibular evoked myogenic potential: new perspectives in multiple sclerosis. *Braz J Otorhinolar* 2005;**71**:48–54. doi:10.1016/s1808-8694(15)31284-2

5 Patkó T, Simó M, Arányi Z. Vestibular click-evoked myogenic potentials: sensitivity and factors determining abnormality in patients with multiple sclerosis. *Mult Scler J* 2007;**13**:193–8. doi:10.1177/1352458506070940

6 Zeigelboim BS, Arruda WO, Mangabeira-Albernaz PL, *et al.* Vestibular findings in relapsing, remitting multiple sclerosis: a study of thirty patients. *Int Tinnitus J* 2008;**14**:139–45.

7 Lima TMA, Crato AN, Mancini PC, *et al.* Alterations in early auditory evoked potentials in multiple sclerosis patients. *Braz J Otorhinolar* 2009;**75**:177–81. doi:10.1016/s1808-8694(15)30775-8

8 Lewis MS, Lilly DJ, Hutter MM, *et al.* Audiometric hearing status of individuals with and without multiple sclerosis. *J Rehabilitation Res Dev* 2010;**47**:669–78. doi:10.1682/jrrd.2010.01.0010

9 Matas CG, Matas SL de A, Oliveira CRS de, *et al.* Auditory evoked potentials and multiple sclerosis. *Arq Neuro-psiquiat* 2010;**68**:528–34. doi:10.1590/s0004-282x2010000400010

10 Doty RL, Tourbier I, Davis S, *et al.* Pure-Tone Auditory Thresholds Are Not Chronically Elevated in Multiple Sclerosis. *Behav Neurosci* 2012;**126**:314–24. doi:10.1037/a0027046

11 Saberi A, Hatamian HR, Nemati S, *et al.* Hearing statement in multiple sclerosis: a case control study using auditory brainstem responses and otoacoustic emissions. *Acta Medica Iranica* 2012;**50**:679–83.

12 Gabeli T, Krbot M, Šefer AB, *et al.* Ocular and Cervical Vestibular Evoked Myogenic Potentials in Patients With Multiple Sclerosis. *J Clin Neurophysiol* 2013;**30**:86–91. doi:10.1097/wnp.0b013e31827eda0c

13 Harirchian MH, Karimi N, Nafisi S, *et al.* Vestibular evoked myogenic potential for diagnoses of multiple sclerosis: is it beneficial? *Medicinski Glasnik Official Publ Medical Assoc Zenica-doboj Canton Bosnia Herzegovina* 2013;**10**:321–6.

For peer review only - http://bmjopen.bmj.com/site/about/guidelines.xhtml

14 Valadbeigi A, Weisi F, Rohbakhsh N, *et al.* Central auditory processing and word discrimination in patients with multiple sclerosis. *Eur Arch Oto-rhino-l* 2014;**271**:2891–6. doi:10.1007/s00405-013-2776-6

15 Pokryszko-Dragan A, Bilinska M, Gruszka E, *et al.* Assessment of visual and auditory evoked potentials in multiple sclerosis patients with and without fatigue. *Neurol Sci* 2015;**36**:235–42. doi:10.1007/s10072-014-1953-8

16 Doty RL, MacGillivray MR, Talab H, *et al.* Balance in multiple sclerosis: relationship to central brain regions. *Exp Brain Res* 2018;**236**:2739–50. doi:10.1007/s00221-018-5332-1

17 Kavasoğlu G, Gökçay F, Yüceyar N, *et al.* Cervical vestibular-evoked myogenic potentials in patients with multiple sclerosis: sensitive in detecting brainstem involvement? *Neurol Sci* 2018;**39**:365–71. doi:10.1007/s10072-017-3215-z

18 Koura R, Hussein M. Vestibular-evoked myogenic potential: an easy neurophysiological tool for evaluating brain stem involvement in multiple sclerosis. *Egypt J Otolaryngology* 2018;**34**:144–8. doi:10.4103/ejo.ejo_73_17

19 Elbeltagy R, Gad N, Ismail M. Central auditory function in multiple sclerosis patients. *Indian J Otology* 2019;**25**:90–6. doi:10.4103/indianjotol.indianjotol_80_18

20 Rishiq D, Harkrider A, Springer C, *et al.* Click-evoked and speech-evoked auditory brainstem responses from individuals with multiple sclerosis. *Neurosci Lett* 2021;**740**:135460. doi:10.1016/j.neulet.2020.135460

21 Srinivasan VS, Krishna R, Munirathinam BR. Effectiveness of Brainstem Auditory Evoked Potentials Scoring in Evaluating Brainstem Dysfunction and Disability Among Individuals With Multiple Sclerosis. *Am J Audiol* 2021;**30**:255–65. doi:10.1044/2020_aja-20-00155

22 Elmoazen D, Kozou H, Gawies M, *et al.* Assessment of Cervical and Ocular Vestibular Evoked Myogenic Potentials in Multiple Sclerosis PatientsS. *Egyptian Journal of Ear, Nose, Throat and Allied Sciences* 2021;**22**:1–9. doi:10.21608/ejentas.2020.44108.1262

23 Versino M, Colnaghi S, Callieco R, *et al.* Vestibular evoked myogenic potentials in multiple sclerosis patients. *Clin Neurophysiol* 2002;**113**:1464–9. doi:10.1016/s1388-2457(02)00155-4

24 Burina A, Sinanović O, Smajlović D, *et al.* Some Aspects of Balance Disorder in Patients with Multiple Sclerosis. *Bosnian J Basic Med* 2008;**8**:80–5. doi:10.17305/bjbms.2008.3003

25 Eleftheriadou A, Deftereos SN, Zarikas V, *et al.* The diagnostic value of earlier and later components of Vestibular Evoked Myogenic Potentials (VEMP) in Multiple Sclerosis. *J Vestib Res* 2009;**19**:59–66. doi:10.3233/ves-2009-0342

26 Degirmenci E, Bir LS, Ardic FN. Clinical and electronystagmographical evaluation of vestibular symptoms in relapsing remitting multiple sclerosis. *Neurol Res* 2010;**32**:986–91. doi:10.1179/016164110x12681290831405

For peer review only - http://bmjopen.bmj.com/site/about/guidelines.xhtml

27 Kaytancı E, Ozdamar OI, Acar GO, *et al.* Evaluation of transiently evoked otoacoustic emissions and auditory brainstem responses in patients with multiple sclerosis. *Ear Nose Throat J* 2016;**95**:E12–7.

28 Di Mauro RD, Di Girolamo SD, Ralli M, *et al.* Subclinical cochlear dysfunction in newly diagnosed relapsing-remitting multiple sclerosis. *Mult Scler Relat Dis* 2019;**33**:55–60. doi:10.1016/j.msard.2019.05.020

29 Parsa MS, Mohammadkhani G, Hajabolhassani F, *et al.* Cervical and ocular vestibular evoked myogenic potentials in multiple sclerosis participants. *Medical J Islamic Repub Iran* 2015;**29**:164.

30 Yang F, Liu X. Relative importance of vision and proprioception in maintaining standing balance in people with multiple sclerosis. *Mult Scler Relat Dis* 2020;**39**:101901. doi:10.1016/j.msard.2019.101901

31 Inojosa H, Schriefer D, Klöditz A, *et al.* Balance Testing in Multiple Sclerosis—Improving Neurological Assessment With Static Posturography? *Front Neurol* 2020;**11**:135. doi:10.3389/fneur.2020.00135

32 Inojosa H, Schriefer D, Trentzsch K, *et al.* Visual Feedback and Postural Control in Multiple Sclerosis. *J Clin Medicine* 2020;**9**:1291. doi:10.3390/jcm9051291

33 Delphi M, Sayaf M, Taheri F, *et al.* Audio and vestibular analyses in patient with multiple sclerosis: a case–control study. *Hear Balance Commun* 2021;**19**:1–6. doi:10.1080/21695717.2021.1933318

34 Cochrane GD, Christy JB, Motl RW. Comprehensive Clinical Assessment of Vestibular Function in Multiple Sclerosis. *J Neurol Phys Ther* 2021;**45**:228–34. doi:10.1097/npt.0000000000000358

35 Cochrane GD, Christy JB, Sandroff BM, *et al.* Cognitive and Central Vestibular Functions Correlate in People With Multiple Sclerosis. *Neurorehab Neural Re* 2021;**35**:1030–8. doi:10.1177/15459683211046268

For peer review only

PRISMA 2020 Checklist

Section and Topic	Item #	Checklist item	Location where item is reported
**TITLE**			
Title	1	Identify the report as a systematic review.	**Page 1 & 4**
**ABSTRACT**			
Abstract	2	See the PRISMA 2020 for Abstracts checklist.	**Page 3 – 4**
**INTRODUCTION**			
Rationale	3	Describe the rationale for the review in the context of existing knowledge.	**Page 4 – 6**
Objectives	4	Provide an explicit statement of the objective(s) or question(s) the review addresses.	**Page 7**
**METHODS**			
Eligibility criteria	5	Specify the inclusion and exclusion criteria for the review and how studies were grouped for the syntheses.	**Page 8 – 10**
Information sources	6	Specify all databases, registers, websites, organisations, reference lists and other sources searched or consulted to identify studies. Specify the date when each source was last searched or consulted.	**Page 7 – 8**
Search strategy	7	Present the full search strategies for all databases, registers and websites, including any filters and limits used.	**Page 8**
Selection process	8	Specify the methods used to decide whether a study met the inclusion criteria of the review, including how many reviewers screened each record and each report retrieved, whether they worked independently, and if applicable, details of automation tools used in the process.	**Page 8 – 9**
Data collection process	9	Specify the methods used to collect data from reports, including how many reviewers collected data from each report, whether they worked independently, any processes for obtaining or confirming data from study investigators, and if applicable, details of automation tools used in the process.	**Page 9 – 10**
Data items	10a	List and define all outcomes for which data were sought. Specify whether all results that were compatible with each outcome domain in each study were sought (e.g. for all measures, time points, analyses), and if not, the methods used to decide which results to collect.	**Page 8**
	10b	List and define all other variables for which data were sought (e.g. participant and intervention characteristics, funding sources). Describe any assumptions made about any missing or unclear information.	**Page 9 – 10**
Study risk of bias assessment	11	Specify the methods used to assess risk of bias in the included studies, including details of the tool(s) used, how many reviewers assessed each study and whether they worked independently, and if applicable, details of automation tools used in the process.	**Page 9**
Effect measures	12	Specify for each outcome the effect measure(s) (e.g. risk ratio, mean difference) used in the synthesis or presentation of results.	**Page 10**
Synthesis methods	13a	Describe the processes used to decide which studies were eligible for each synthesis (e.g. tabulating the study intervention characteristics and comparing against the planned groups for each synthesis (item #5)).	**Not Applicable**
	13b	Describe any methods required to prepare the data for presentation or synthesis, such as handling of missing summary statistics, or data conversions.	**Not Applicable**
	13c	Describe any methods used to tabulate or visually display results of individual studies and syntheses.	**Not Applicable**
	13d	Describe any methods used to synthesize results and provide a rationale for the choice(s). If meta-analysis was performed, describe the model(s), method(s) to identify the presence and extent of statistical heterogeneity, and software package(s) used.	**Not Applicable**
	13e	Describe any methods used to explore possible causes of heterogeneity among study results (e.g. subgroup analysis, meta-regression).	**Not Applicable**
	13f	Describe any sensitivity analyses conducted to assess robustness of the synthesized results.	**Not Applicable**
Reporting bias assessment	14	Describe any methods used to assess risk of bias due to missing results in a synthesis (arising from reporting biases).	**Not Applicable**
Certainty assessment	15	Describe any methods used to assess certainty (or confidence) in the body of evidence for an outcome.	**Not Applicable**
**RESULTS**			

For peer review only - http://bmjopen.bmj.com/site/about/guidelines.xhtml

PRISMA 2020 Checklist

Section and Topic	Item #	Checklist item	Location where item is reported
Study selection	16a	Describe the results of the search and selection process, from the number of records identified in the search to the number of studies included in the review, ideally using a flow diagram.	**Page 10 & Figure 1**
	16b	Cite studies that might appear to meet the inclusion criteria, but which were excluded, and explain why they were excluded.	**Not applicable**
Study characteristics	17	Cite each included study and present its characteristics.	**Table 1 and Table 2**
Risk of bias in studies	18	Present assessments of risk of bias for each included study.	**Supplement Tables S1 – S3**
Results of individual studies	19	For all outcomes, present, for each study: (a) summary statistics for each group (where appropriate) and (b) an effect estimate and its precision (e.g. confidence/credible interval), ideally using structured tables or plots.	**(a) Supplement Tables S4 – S7**<br>**(b) Not applicable**
Results of syntheses	20a	For each synthesis, briefly summarise the characteristics and risk of bias among contributing studies.	**Not applicable**
	20b	Present results of all statistical syntheses conducted. If meta-analysis was done, present for each the summary estimate and its precision (e.g. confidence/credible interval) and measures of statistical heterogeneity. If comparing groups, describe the direction of the effect.	**Not applicable**
	20c	Present results of all investigations of possible causes of heterogeneity among study results.	**Not applicable**
	20d	Present results of all sensitivity analyses conducted to assess the robustness of the synthesized results.	**Not applicable**
Reporting biases	21	Present assessments of risk of bias due to missing results (arising from reporting biases) for each synthesis assessed.	**Not applicable**
Certainty of evidence	22	Present assessments of certainty (or confidence) in the body of evidence for each outcome assessed.	**Not applicable**
**DISCUSSION**			
Discussion	23a	Provide a general interpretation of the results in the context of other evidence.	**Page 18 – 24**
	23b	Discuss any limitations of the evidence included in the review.	**Page 18 – 24**
	23c	Discuss any limitations of the review processes used.	**Not applicable**
	23d	Discuss implications of the results for practice, policy, and future research.	**Page 18 – 24**
**OTHER INFORMATION**			
Registration and protocol	24a	Provide registration information for the review, including register name and registration number, or state that the review was not registered.	**Abstract & Methods**
	24b	Indicate where the review protocol can be accessed, or state that a protocol was not prepared.	**Abstract & Methods**
	24c	Describe and explain any amendments to information provided at registration or in the protocol.	**Not applicable**
Support	25	Describe sources of financial or non-financial support for the review, and the role of the funders or sponsors in the review.	**Not applicable**
Competing interests	26	Declare any competing interests of review authors.	**Page 25**
Availability of data, code and other materials	27	Report which of the following are publicly available and where they can be found: template data collection forms; data extracted from included studies; data used for all analyses; analytic code; any other materials used in the review.	**Not applicable**

From: Page MJ, McKenzie JE, Bossuyt PM, Boutron I, Hoffmann TC, Mulrow CD, et al. The PRISMA 2020 statement: an updated guideline for reporting systematic reviews. BMJ 2021;372:n71. doi: 10.1136/bmj.n71

For more information, visit: http://www.prisma-statement.org/

For peer review only - http://bmjopen.bmj.com/site/about/guidelines.xhtml

BMJ Open

The effects of Multiple Sclerosis on the audio-vestibular system: A systematic review

Journal:	*BMJ Open*
Manuscript ID	bmjopen-2021-060540.R1
Article Type:	Original research
Date Submitted by the Author:	31-Mar-2022
Complete List of Authors:	Gür, Evrim; The University of Manchester, Manchester Centre for Audiology and Deafness (ManCAD), School of Health Sciences, Ellen Wilkinson Building Manchester, UK M13 9PL<br>Binkhamis, Ghada; King Fahad Medical City, Communication and Swallowing Disorders; The University of Manchester, Manchester Centre for Audiology and Deafness (ManCAD)<br>Kluk, Karolina; The University of Manchester, Manchester Centre for Audiology and Deafness (ManCAD), School of Health Sciences, Ellen Wilkinson Building Manchester, UK M13 9PL
**Primary Subject Heading**:	Ear, nose and throat/otolaryngology
Secondary Subject Heading:	Neurology
Keywords:	Audiology < OTOLARYNGOLOGY, Adult otolaryngology < OTOLARYNGOLOGY, Multiple sclerosis < NEUROLOGY

SCHOLARONE™
Manuscripts

I, the Submitting Author has the right to grant and does grant on behalf of all authors of the Work (as defined in the below author licence), an exclusive licence and/or a non-exclusive licence for contributions from authors who are: i) UK Crown employees; ii) where BMJ has agreed a CC-BY licence shall apply, and/or iii) in accordance with the terms applicable for US Federal Government officers or employees acting as part of their official duties; on a worldwide, perpetual, irrevocable, royalty-free basis to BMJ Publishing Group Ltd ("BMJ") its licensees and where the relevant Journal is co-owned by BMJ to the co-owners of the Journal, to publish the Work in this journal and any other BMJ products and to exploit all rights, as set out in our licence.

The Submitting Author accepts and understands that any supply made under these terms is made by BMJ to the Submitting Author unless you are acting as an employee on behalf of your employer or a postgraduate student of an affiliated institution which is paying any applicable article publishing charge ("APC") for Open Access articles. Where the Submitting Author wishes to make the Work available on an Open Access basis (and intends to pay the relevant APC), the terms of reuse of such Open Access shall be governed by a Creative Commons licence – details of these licences and which Creative Commons licence will apply to this Work are set out in our licence referred to above.

Other than as permitted in any relevant BMJ Author's Self Archiving Policies, I confirm this Work has not been accepted for publication elsewhere, is not being considered for publication elsewhere and does not duplicate material already published. I confirm all authors consent to publication of this Work and authorise the granting of this licence.

For peer review only

The effects of Multiple Sclerosis on the audio-vestibular system: A systematic review

Evrim Gür[1,*], Ghada BinKhamis[1,2,*] and Karolina Kluk[1]

[1]*Manchester Centre for Audiology and Deafness, Manchester Academic Health Science Centre, University of Manchester, Manchester, UK;* [2]*Department of Communication and Swallowing Disorders, King Fahad Medical City, Riyadh, Saudi Arabia*

*E.G. and G.B. equally contributed to this work

Address for correspondence:

Ghada BinKhamis: ghada.binkhamis@manchester.ac.uk / gbinkhamis@gmail.com

Karolina Kluk: Karolina.Kluk@manchester.ac.uk

Evrim Gür: evrimguur@gmail.com,

Manchester Centre for Audiology and Deafness (ManCAD), School of Health Sciences, Faculty of Biology Medicine and Health, Room B2.15, Ellen Wilkinson Building, University of Manchester, Oxford Road, Manchester, M13 9PL, United Kingdom.

ORCiD:

1. Ghada BinKhamis: 0000-0002-7696-1073
2. Karolina Kluk: 0000-0003-3638-2787

Word count: 5570

For peer review only

ABSTRACT

Objective: Systematically investigate effects of Multiple Sclerosis (MS) on the audio-vestibular system.

Methods: Systematic review of literature investigating audio-vestibular conditions in persons with MS (PwMS) aged ≥ 18 years. PUBMED, SCOPUS, NICE, Web of Science were searched. Randomised control trials, cohort, case-control, observational, retrospective studies in English, published from 2000 to 21st November 2021, evaluated PwMS with at least one outcome (Pure Tone Audiometry, Auditory Brainstem Response, Otoacoustic Emissions, Cortical Auditory Evoked Potentials, Functional MRI assessing auditory function, Vestibular Evoked Myogenic Potentials, Videonystagmography, Electonystagmography, Posturography, Rotary Chair, Gaps-in-Noise, Word Discrimination Scores, Duration Pattern Sequence Test) were included. Study selection and assessments of bias were independently conducted by two reviewers using the Risk of Bias Assessment Tool for Nonrandomized Studies, Newcastle Ottawa Scale (NOS), and the NOS adapted for cross-sectional studies.

Results: 35 studies were included. Auditory function was evaluated in 714 PwMS and 501 controls, vestibular function was evaluated in 682 PwMS and 446 controls. Peripheral auditory function results were contradictory between studies; some found abnormalities in PwMS, and others found no differences. Tests of brainstem and central auditory functions were more consistently found to be abnormal in PwMS. Most vestibular tests were reported as abnormal in PwMS, abnormalities were either peripheral or central or both. However, quantitative analyses could not be performed due to discrepancies between studies in results reporting, test stimulus and recording parameters.

Conclusions: Although abnormal results on auditory and vestibular tests were noted in PwMS, specific effects of MS on the audio-vestibular system could not be determined due to the heterogeneity between studies that restricted the ability to conduct any quantitative analyses.

For peer review only - http://bmjopen.bmj.com/site/about/guidelines.xhtml

Further research with consistent reporting, consistent stimulus and consistent recording parameters are needed in order to quantify the effects of MS on the auditory and vestibular systems.

Prospero Registration: CRD42020180094

Strengths and limitations of this study:

- This study provides a comprehensive systematic review of the literature on MS and the auditory and vestibular systems from 2000 until 21st November 2021.
- This is the only systematic review that investigated the effects of MS on the audio-vestibular system.
- Differences between studies in reporting of results and in test procedures restricted the ability to conduct any form of quantitative analyses.

Keywords: Multiple sclerosis, auditory function, vestibular function, audio-vestibular system

For peer review only

The effects of Multiple Sclerosis on the audio-vestibular system: A systematic review

INTRODUCTION

Multiple Sclerosis (MS) is a chronic, immune-mediated, inflammatory disease that causes a neurodegenerative process in the Central Nervous System (CNS).[1] It is an autoimmune disease with four main MS subtypes: Relapsing-remitting (RRMS), Primary-Progressive (PPMS), Secondary Progressive (SPMS) and Progressive Relapsing (PRMS). The autoimmune process of MS results in CNS plaques (focal areas of demyelination) in addition to axonal injury or loss.[2,3] These plaques most commonly occur in the white matter of the brain, cerebral cortex including subpial regions, spinal cord and optic nerve,[4] resulting in optic neuritis, double vision, tremor, ataxic gait, weakness and numbness in one or more limbs.[5] It has been suggested that demyelination plaques can also occur in the central auditory and vestibular pathways resulting in hearing loss and balance disorders.[6] For example, plaques may occur in the brainstem, where both efferent and afferent auditory and vestibular pathways are present.[6] Also, the vestibular nuclei and the root entry zone of the eighth cranial nerve have been shown to be one of the most common neuroanatomic locations for inflammatory demyelination;[7] In addition, peripheral neural connections in the internal auditory canal and within the inner ear structures may be affected by demyelination resulting in peripheral auditory and vestibular involvement in persons with MS (PwMS).[8–10] The extent of occurrence of audio-vestibular symptoms in PwMS is unclear, as reports on their prevalence vary in the literature. For example, one study reported a prevalence of audio-vestibular symptoms between 1% and 28%.[11] Whereas others reported that vertigo is seen in 20 – 35 % of PwMS, and hearing loss is seen in 1 – 17 % of PwMS.[12–15] While a recent systematic review on Magnetic Resonance Imaging (MRI) and auditory tests found that 25 % of PwMS had either sudden or progressive hearing loss.[11]

For peer review only - http://bmjopen.bmj.com/site/about/guidelines.xhtml

Literature on the involvement of the auditory system in PwMS is either contradictory or limited. First, several studies that assessed the effects of MS on the peripheral auditory system through evaluating hearing thresholds via Pure Tone Audiometry (PTA) found worse PTA thresholds in PwMS compared to healthy controls;[16–18] while one study did not find any significant differences in PTA thresholds between PwMS and healthy controls.[19] Second, some studies that assessed the effects of MS on the brainstem auditory pathway via Auditory Brainstem Responses (ABR) found that PwMS had abnormal ABRs;[11,20] while one study found no differences in ABRs between PwMS and healthy controls.[21] Finally, only a few studies investigated the effects of MS on speech understanding as assessed via Word Discrimination Scores (WDS) and on temporal processing as assessed via the Gaps-In-Noise (GIN) test. These found poorer WDS in noise and poorer performance on the GIN in PwMS compared to healthy controls.[22,23]

Literature on the involvement of the vestibular system in PwMS generally found abnormalities in PwMS. Studies that assessed the vestibular system via Electronystagmography (ENG) found abnormal results in PwMS on tests of peripheral vestibular function, central vestibular function or both.[24–27] Other studies that investigated the vestibular pathway via Vestibular Evoked Myogenic Potentials (VEMPs) in PwMS found abnormalities in VEMP results including latency delays, reduced amplitudes and inter-aural side differences.[28–39] Further studies investigated the functional integrity of the vestibular system in PwMS via static and dynamic posturography, and found that PwMS performed in general worse than healthy controls.[40–42] Although literature has shown abnormalities on different tests of vestibular function in PwMS, different studies used different test procedures, different parameters, or reported different components of each test result (e.g. VEMP latency versus amplitude).

In summary, numerous studies performed different audio-vestibular test batteries in PwMS. These studies aimed to uncover possible MS-related changes in auditory or vestibular function.

For peer review only - http://bmjopen.bmj.com/site/about/guidelines.xhtml

However, results from literature that investigated auditory function varied, as some indicated no differences, while others reported considerable differences between PwMS and healthy controls. Whereas results from literature on the effects of MS on vestibular function generally showed abnormalities in PwMS, the effects of MS on central versus peripheral vestibular function are still unclear. Therefore, literature needs to be combined and summarised to better understand the potential effects of MS on the auditory and/or vestibular systems. If auditory and/or vestibular disorders in PwMS are better understood, early identification and early intervention could occur, resulting in improved quality of life. This highlights the need for this systematic review with a focus on the effects of MS on the audio-vestibular system. We hypothesise that PwMS experience auditory and vestibular disorders at a higher rate than the general population. This systematic review aims to investigate this hypothesis by examining and synthesising current literature on the effects of MS on the audio-vestibular system.

METHODS

This systematic review followed the Preferred Reporting Items for Systematic reviews and Meta-Analyses (PRISMA) statement,[43] and was pre-registered in PROSPERO (CRD42020180094)

Patient and public involvement

No patient involved

Research ethics approval

As new participant data were not collected, Ethics Approval was not needed.

Data Sources and Searches

A systematic search was conducted in the following electronic bibliographic databases: PubMed, NICE, Scopus and Web of Science using the following terms: “Multiple Sclerosis”, “Auditory Function”, “Vestibular Function”, “Hearing Loss”, “Dizziness”, “Vertigo”.

For peer review only

114 Operators (AND, OR) were used to narrow the search (see Supplement for detailed search strategy). A total of 21 searches were performed by E.G. up until November 21st 2021. No language or date restrictions were applied.

117 Inclusion and exclusion criteria

118 Studies were eligible for inclusion if they met the following criteria: 1) investigated audio-vestibular conditions; 2) participants were diagnosed with any type of MS (RRMS, SPMS, PPMS, PRMS); 3) participants were aged 18 years and older because of maturational effects on outcomes of some audio-vestibular tests; 4) one or more of the pre-selected tests described below were used to evaluate auditory or vestibular function; 5) any of the following study types: randomised controlled trials, cohort, case-controlled, observational, or retrospective studies; 6) results from PwMS were either compared to a healthy control group or to normative data; 7) any geographical location. Studies were excluded based on the following: 1) did not include the following keywords "Multiple Sclerosis", "dizziness", "auditory function", "vestibular", "vertigo" or "hearing loss" in the title or abstract; 2) no available full text in English; 3) published before the year 2000; 4) participants were diagnosed with comorbid diseases.

129 Audio-vestibular tests

130 Evaluation of auditory function was restricted to the following measures: Pure Tone Audiometry (PTA), Auditory Brainstem Response (ABR), Otoacoustic Emissions (OAEs: Transient Evoked (TEOAE) and Distortion Product (DPOAE)), Cortical Auditory Evoked Potentials (CAEP) and Functional MRI assessing auditory function. Evaluation of vestibular function was restricted to the following measures: Vestibular Evoked Myogenic Potentials (VEMPs) including Cervical (cVEMPs) and Ocular (oVEMPs), Videonystagmography (VNG), Electonystagmography (ENG), Posturography and Rotary Chair. Above tests were defined as primary outcomes, the secondary outcomes were defined as measures of speech perception and temporal processing, specifically; Gaps in Noise test (GIN), Word Discrimination Scores

For peer review only - http://bmjopen.bmj.com/site/about/guidelines.xhtml

(WDS) and Duration Pattern Sequence Test (DPST). (Refer to supplement for details on which measures and parameters were extracted for each test).

Study selection

E.G. manually removed duplicates and screened titles and abstracts for inclusion and exclusion criteria. Remaining full texts were independently reviewed by E.G. and G.B., and any disagreements were resolved by discussion or by involving K.K. until a consensus was reached.

Assessment of bias

Risk of bias assessment was done independently by E.G. and G.B.. Case-control studies were assessed using the Risk of Bias Assessment Tool for Nonrandomized Studies (RoBANS [44]) to examine the selection of participants, confounding variables, measurement of exposure, blinding of outcome assessment, incomplete outcome of data and selective outcome reporting. Cohort studies were assessed with the Newcastle Ottawa Scale (NOS [45]). A judgement of 'low risk' of bias, 'high risk' or bias, or 'unclear risk' of bias was provided for each domain. Cross-sectional studies were evaluated with the NOS adapted for cross-sectional studies.[46] Any disagreements were resolved by discussion or by involving the third reviewer (K.K.) until a consensus was reached.

Data extraction and synthesis

The following data were extracted when available: 1) Study details: aims and design. 2) Population: recruitment methods; diagnosis and inclusion/exclusion criteria; syndrome differentiation; course of disease; number of participants: screened, eligible, enrolled, included in the analysis; age; gender. 3) Details of the pathology: specific lesion site, primary or secondary, stable or progressive, additional handicaps related to MS. 4) Methodology: test procedure, stimulus and recording parameters, electrode impendence and inter-electrode impedance levels. 5) Test results, e.g. PTA thresholds, VEMP amplitudes, ABR peak latencies, etc.

For peer review only

E.G. extracted the data and G.B. confirmed the accuracy of all extracted data. Any disagreements were resolved by discussion between the two reviewers. Per pre-registered protocol, investigators were not contacted for unreported data or additional information due to time constraints and given current COVID-19 pandemic related global circumstances.

Criteria for data synthesis: studies that used the same test procedure (either PTA, ABR or VEMP), minimum five participants in each study, minimum two studies for each test method, and the same stimulus and recording parameters were used. Mean, Standard Deviation (SD), and/or individual values were extracted when available, however, the effect size could not be calculated due to inconsistencies between studies in stimulus and recording parameters or in how results were reported.

RESULTS

Description of included studies

3876 studies were identified initially, and 1484 studies were identified after duplicate removal and primary limitations (Figure 1); 1055 were excluded because they did not relate to audio-vestibular conditions, 42 due to no full text availability, 33 were not in English, 177 due to the use of tests other than those in the inclusion criteria, and 87 were excluded because they did not have an abstract. The remaining 90 studies were examined for inclusion, eight were excluded because they were case reports, 22 did not evaluate audio-vestibular conditions, six used tests other than those in the inclusion criteria, 13 included participants under 18 years of age, and six additional duplicates were removed.

A total of 35 studies underwent full-length review. Fourteen evaluated the auditory system, 18 evaluated the vestibular system and three evaluated both auditory and vestibular systems.

Assessment of bias

Case-control studies assessed with RoBANS (Supplement Table S1) were judged as low risk of bias in most categories, except for blinding of outcome assessment that was judged as high

For peer review only

For peer review only - http://bmjopen.bmj.com/site/about/guidelines.xhtml

risk of bias in all 22 studies. Cohort studies assessed with NOS (Supplement Table S2) were judged as either good (*n* = 2), poor (*n* = 2), or unknown (*n* = 2, rating could not be assigned due to one or more category that was judged as "not applicable" e.g. two studies were evaluated as not applicable for "selection of the non-exposed group" due to lack of a control group). Cross-sectional studies were assessed using NOS adapted for cross-sectional (Supplement Table S3) and most were judged as good (*n* = 6) with one judged as poor.

MS and auditory function

Seventeen studies investigated the auditory systems involvement in PwMS (Table 1 – see Supplement Table S4 for an alternative table format summarizing findings based on outcome): three assessed OAEs, five assessed PTA, 13 evaluated ABRs, two investigated CAEPs, and three of the 17 studies performed speech perception and/or temporal processing tests. No studies that conducted functional MRI to assess auditory function were located.

Three studies compared OAEs in PwMS and healthy controls with a total number of 218 participants, 120 PwMS and 98 controls. Two studies did not find differences between PwMS and controls in TEOAEs and/or DPOAEs.[14,47] However, the third study found weaker responses in both TEOAEs and DPOAEs in PwMS compared to healthy controls.[21]

Five studies performed PTA on PwMS to assess hearing levels. A total of 510 participants (265 PwMS and 245 healthy controls) were evaluated with PTA, sample size varied from 80 to 146 and age range was between 18 and 65 years. Two out of five studies reported no significant difference in PTA thresholds between PwMS and controls,[19,21]. One reported worse PTA thresholds only in female PwMS compared to female controls,[13] and two reported worse thresholds in PwMS compared to controls,[18,48] with one finding worse thresholds in participants with SPMS when compared to RRMS.[18] Three of five studies provided mean and SD values of PTA thresholds (Supplement Table S5). However, reported values could not be pooled because the way that authors presented the data differed between each other. For

For peer review only - http://bmjopen.bmj.com/site/about/guidelines.xhtml

instance, Doty et al. (2012) reported average PTA thresholds across right and left ears for each frequency in male and female participants separately,[19] while Di Mauro et al. (2019) reported values for each ear separately for all participants.[21]

Thirteen studies assessed ABRs (Table 1) and included 881 participants (548 PwMS and 333 healthy controls), sample size varied from 25 to 126 and age range was between 18 and 68 years. Twelve of the 13 studies reported abnormalities in different ABR components in PwMS as follows:

- Prolonged absolute latencies of wave I[47] III and V.[47–50]
- Prolonged interpeak latencies of I–III,[14,28,30,48–53] III–V,[14,28,30,48,50–53] and I–V.[20,28,47–51,53]
- Interaural interpeak latency differences for I–III and I–V[28] and III–V.[28,51]
- Absent waves III and V.[28,30,48,51,52]
- Absent waves at high stimulus presentation rates.[54]
- Reduced wave V amplitude.[53]
- Increased I/III and I/V amplitude ratios.[51]
- Reduced V/I amplitude ratio.[48,50]
- Poor waveform morphology and/or only wave I within normal limits.[20]

Only Di Mauro et al. (2019) did not find any differences in ABRs between PwMS and controls.[21] Six out of 13 studies shared mean, and SD values for latencies, amplitudes and/or inter-aural latency differences (Supplement Table S6); however, data could not be combined, and the effect size calculation was not performed due to the variability in stimulus and recording parameters. For example, Matas et al. (2010) presented rarefaction clicks at 80 dB nHL with a presentation rate of 19.9 clicks per second,[49] while Kaytancı et al. (2016) presented clicks (polarity unspecified) at 70 dB nHL with a presentation rate of 13.0 clicks per second.[47]

For peer review only

Cortical evoked potentials were evaluated by two studies using two different tests (Table 1). Japaridze et al. (2002) reported 30% abnormality in slow cortical potentials (SCPs); abnormalities were either absent peaks or prolonged peak latencies.[51] Matas et al. (2010) assessed P300 peak latency (defined as a cognitive potential) and did not find any significant differences between PwMS and healthy controls; however, percentage of abnormalities in P300 results was higher in the PwMS (16%) compared to controls (0%).[49]

Three studies assessed speech perception and temporal processing (Table 1) with total of 188 participants, 93 PwMS and 95 healthy controls and an age range of 18-65 years. Two of the three found that PwMS had poorer WDS in noise and an increased approximate threshold plus lower percent correct answers on the GIN test compared to controls;[22,23] and one of the two also found that PwMS had lower percent correct answers on the DPST.[22] However, the third study found no significant difference between PwMS and controls on WDS in quiet.[18]

For peer review only

Table 1. *Characteristics of studies examining the effects of MS on auditory function*

Author (Year)	*Study Type*	*Sample Size*		*PwMS Mean Age (years)*	*PwMS Age range (Years)*	*% Female*	*MS Type (sample size)*	*MS Duration Mean (range) years*	*Test Battery*	*Results of PwMS compared to controls / normative data*
		PwMS	*Healthy Controls*							
Japaridze et al. (2002)[51]	Case-Control	40	33	30.5	18-57	77.5	?	?	ABR<br>CAEP (SCP)	65% Abnormal<br>30% Abnormal
Versino et al. (2002)[28]	Cohort	65	18	35.5	19-61	?	?	?	ABR	37.5% Abnormal
Burina et al. (2008)[52]	Cohort	60	NA	37.2	?	68.3	RRMS	?	ABR	95% Abnormal
Eleftheriadou et al. (2009)[30]	Cohort	46	40	40	20-66	45.6	RRMS	4.6	ABR	26% Abnormal
Lima et al. (2009)[20]	Case-control	25	NA	42.6 (female)<br>38 (male)	33-53 (female)<br>24-56 (male)	64.0	RRMS (14), PPMS (8), Unspecified Progressive (3)	?	ABR	30% Abnormal
Lewis et al. (2010)[18]	Case-control	47	49	51.4	21-65	57.7 RRMS, 28.6 SPMS	RRMS (26), SPMS (21)	RRMS: 12.6 (2-43)<br>SPMS: 23.3 (8-50)	PTA<br>WDS	Worse thresholds<br>No significant difference
Matas et al. (2010)[49]	Case-control	25	25	34.88	25-55	76.0	RRMS	4.25 (?)	ABR<br>CAEP (P300)	Significantly different<br>16% Abnormal
Doty et al. (2012)[19]	Case-control	73	73	Males: 45.24<br>Females: 45.6	?	71.2	RRMS (57), PPMS (3), SPMS (6), Unspecified (7)	Males: 7.36 (?)<br>Females: 7.84 (?)	PTA	No significant difference
Saberi et al. (2012)[14]	Case-control	60	38	29.9	?	73.3	?	3.2 (?)	PTA<br>TEOAE & DPOAE<br>ABR	Worse thresholds in female PwMS<br>No significant difference<br>20% Abnormal
Valadbeigi et al. (2014)[22]	Case-control	26	26	28.9	18-40	?	RRMS	?	GIN<br>WDS<br>DPST	Significantly different<br>Lower scores PwMS<br>Significantly different
Pokryszko-Dragan et al. (2015)[53]	Case-control	86	40	39.55	19-60	72.0	?	8.57 (1-30)	ABR	Significantly different
Kaytancı et al. (2016)[47]	Cohort	20	20	31.3	?	55.0	?	?	TEOAE<br>ABR	No significant difference<br>Significantly different
Di Mauro et al. (2019)[21]	Cohort	40	40	37	18-50	60.0	RRMS	0.8 (0.8-2)	PTA<br>TEOAE & DPOAE<br>ABR	No significant difference<br>Significantly lower<br>No difference
Elbeltagy et al. (2019)[23]	Case-control	20	20	37.6	30-50	?	RRMS	?	GIN<br>WDS	Significantly different<br>Significantly lower
Delphi et al. (2021)[50]	Cross-sectional	25	25	31.43	18-45	72.0	?	?	ABR	Significantly different
Rishiq et al. (2021)[54]	Case-control	11	9	49.5	34-68	72.7	RRMS (9), PPMS (1), SPMS (1)	11.9 (2-30)	ABR	Significantly different at high click rate
Srinivasan et al. (2021)[48]	Case-control	45	45	31.77	18-50	73.3	RRMS	6.1 (5.37)	PTA<br>ABR	Significantly different<br>Significantly different and 55.56 % abnormal

Abbreviations: PTA= Pure Tone Audiometry, ABR= Auditory Brainstem Response, OAE= Oto-acoustic Emissions, TEOAE= Transient Evoked Oto acoustic emission, DPOAE= Distortion Product Oto acoustic Emissions, CAEP= Cortical Auditory Evoked Potentials, SCP= Slow Cortical Potentials, GIN= Gaps in Noise test, WDS= Word Discrimination Scores, DPST= Duration pattern sequence test, MS= Multiple Sclerosis, PwMS = Persons with Multiple Sclerosis, ? = not reported.

Cells shaded in grey represent test results that were abnormal or showed a difference between PwMS and healthy controls

For peer review only

For peer review only - http://bmjopen.bmj.com/site/about/guidelines.xhtml

MS and vestibular function

A total of 21 studies that investigated the vestibular system in PwMS were identified (Table 2 – see Supplement Table S7 for an alternative table format summarizing findings based on outcome). Two studies investigated vestibular function using ENG; three used static posturography, one used dynamic posturography, 14 used cVEMPs and four also used oVEMPs, and two used Rotary Chair testing.

A total of 90 participants (60 PwMS and 30 healthy controls) were evaluated using ENG, age ranged between 24 and 64. Both studies found ENG abnormalities in the peripheral and central vestibular systems in PwMS.[27,55] Degirmenci et al. (2010) noted that 90% of PwMS showed ENG abnormalities compared to 6.7% of the controls, and there was a significant difference between groups.[27] Abnormalities suggestive of central vestibular pathology were found in 83.3% of PwMS and in 6.7% of controls, and abnormalities suggestive of peripheral vestibular pathology were found in 36.7% of PwMS and in 0% of controls.[27] Zeigelboim et al. (2008) also reported ENG abnormalities in 86.7% (83.4% Peripheral) of their PwMS.[55]

Four studies assessed posturography with a total of 314 participants (187 PwMS and 127 healthy controls). All four studies found significant differences between PwMS and controls.[40,41,56,57] Two studies reported static posturography results for the same sample,[40,41] they noted increased delineated area, average sway and speed of sway for both open and closed eyes in PwMS compared to controls. A third study also conducted static posturography and found that sway area and path length were significantly greater in PwMS than in controls.[56] The fourth study evaluated dynamic posturography and found longer motor control test latencies, lower equilibrium scores on the sensory organisation tests, and a compromised ability to use three sensory inputs (somatosensory, vestibular, and visual preference) in PwMS compared to controls.[57]

For peer review only

A total of 14 studies assessed VEMPs and included 724 participants (435 PwMS and 289 healthy controls), sample size varied from 30 to 88, and age range was between 18 and 71 years. Four studies evaluated both oVEMPs and cVEMPs[35,58–60] and 10 studies evaluated cVEMPs.[28–30,50,61–66] Thirteen of the 14 studies reported abnormalities in different VEMP components in PwMS as follows:

- Prolonged latencies of p13,[28–30,35,50,58,60–66] n23,[28,30,35,50,58,60–66] and p1 and n1.[35,58,60]
- Reduced p13-n23 amplitude.[28,50,61,63]
- Inter-side asymmetry for p13,[28,29,63] and n23 and n23 latencies.[28]
- Absent waves.[30,62,63,66]

Ten of 14 studies compared VEMPs in PwMS to healthy controls.[30,35,50,58–63,65] Except for two,[59,65] all found a significant difference between PwMS and controls. The remaining VEMP studies used data from controls as normative data.[28,29,64,66]

Thirteen studies shared mean and SD values of p13, n23, p1 and n1 latencies (Supplement Tables S8 and S9). However, data could not be analysed because intensity level, stimulus type, stimulus polarity and other VEMP recording parameters differed between studies. For instance, Sartucci and Logi (2002) presented clicks at 140 dBSPL at a rate of three per second,[61] Aidar and Suzuki (2005) presented clicks at 95 dBHL at a rate of two per second,[62] Kavasoğlu et al. (2017) presented clicks at 100 dBnHL at a rate of five per second,[65] and Koura and Hussein (2018) presented a 500-Hz tone burst at 95 dBHL.[66]

Two studies conducted Rotary Chair testing. However, they tested and reported results from the same sample.[59,67] Forty PwMS and 20 healthy controls were tested, results showed that PwMS had lower vestibular-ocular reflex (VOR) cancellation gain with a visual target and larger variances in their responses on both the subjective visual vertical and horizontal (SVV/H)

For peer review only

For peer review only - http://bmjopen.bmj.com/site/about/guidelines.xhtml

tests compared to controls.[59] Also, that PwMS performed worse than controls on central vestibular measures but not on peripheral.[67]

For peer review only

For peer review only - http://bmjopen.bmj.com/site/about/guidelines.xhtml

Table 2. *Characteristics of studies examining the effects of MS on vestibular function*

Author (Year)	*Study Type*	*Sample Size*		*Mean Age PwMS (years)*	*PwMS Age range (Years)*	*% Female*	*MS Type (sample size)*	*MS Duration Mean (range) years*	*Test Battery*	*Results of PwMS compared to controls / normative data*
		PwMS	*Healthy Controls*							
Sartucci & Logi (2002)[61]	Case-Control	15	15	44.5	26-59	66.6	?	?	cVEMP	Significantly different
Versino et al. (2002)[28]	Cohort	70	18	35.5	19-61	?	?	?	cVEMP	31.4% Abnormal
Alpini et al. (2004)[29]	Case-control	40	25	38	17-71	57.5	?	?	cVEMP	70% Abnormal
Aidar and Suzuki (2005)[62]	Case-control	15	15	39.3	?	?	?	?	cVEMP	Significantly different
Patkó et al. (2007)[63]	Case-control	30	30	43.4	27-60	66.6	?	?	cVEMP	Significantly different
Zeigelboim et al. (2008)[55]	Case-control	30	0	42.23	27-64	80	RRMS	?	ENG	86.7% Abnormal
Eleftheriadou et al. (2009)[30]	Cohort	46	40	40	20-66	45.6	RRMS	4.6 (?)	cVEMP	Significantly different
Degirmenci et al. (2010)[27]	Cohort	30	30	37.9	23-56	56.7	RRMS	?	ENG	90% Abnormal
Gabelić et al. (2013)[58]	Case control	30	15	?	?	46.6	RRMS	3.93 (0.2-21)	cVEMP<br>oVEMP	Significantly different<br>Significantly different
Harirchian et al. (2013)[64]	Case-control	20	20	30	20-40	50	RRMS<br>SPMS	?	cVEMP	70% Abnormal
Parsa et al. (2015)[35]	Cross-sectional	34	15	29.8	?	100	?	?	cVEMP<br>oVEMP	Significantly different<br>Significantly different
Doty et al. (2018)[57]	Case-control	58	72	Males:44.61<br>Females: 44.60	?	68.9	?	Male: 7.03 (?)<br>Female: 6.54 (?)	Dynamic Posturography	Significantly different
Kavasoğlu et al. (2018)[65]	Case-control	30	31	30	18-45	60.0	?	< 1 (?)	cVEMP	23.3% Abnormal<br>No significant difference
Koura and Hussein (2018)[66]	Case-control	20	10	36.80	?	65.0	?	4.4 (?)	cVEMP	100% Abnormal
Inojosa et al. (2020)[40,41]	Cross-sectional	99	30	35.01	18-50	68.7	?	5.5 (?)	Static Posturography	Significantly different
Yang and Liu (2020)[56]	Cross-sectional	30	25	50.8	?	76.7	?	14 (?)	Static Posturography	Significantly different
Delphi et al. (2021)[50]	Cross-sectional	25	25	31.43	18-45	72.0	?	?	cVEMP	Significantly different
Elmoazen et al. (2021)[60]	Case-control	20	10	Brainstem lesions: 40<br>No Brainstem lesion: 34.1	?	?	?	?	cVEMP<br>oVEMP	Significantly different<br>Significantly different
Cochrane et al. (2021)[59,67]	Cross-sectional	40	20	42.4	21-55	88.0	RRMS	9.9	Rotary Chair<br>cVEMP<br>oVEMP	Significantly different<br>No significant difference<br>No significant difference

Abbreviations: cVEMP = Cervical Vestibular Evoked Myogenic Potentials, oVEMP = Ocular Vestibular Evoked Myogenic Potentials, MS= Multiple Sclerosis, PwMS = Persons with Multiple Sclerosis, ? = not reported.
Cells shaded in grey represent test results that were abnormal or showed a difference between PwMS and healthy controls.

For peer review only - http://bmjopen.bmj.com/site/about/guidelines.xhtml

DISCUSSION

In this systematic review, 35 studies were reviewed: 22 case-control studies, six cohort studies, and seven cross-sectional studies. The aim was to investigate the effects of MS on the audio-vestibular system by examining and synthesising current literature that used different auditory and vestibular tests. Additionally, this systematic review presents an overview of the last 21 years of literature specific to MS in the context of the auditory and vestibular systems. The selected outcome measures for the auditory system were: OAE, PTA, ABR, CAEP, WDS, GIN, DPST. Vestibular system outcome measures were: VNG, ENG, Rotary Chair, VEMPs, Static and dynamic Posturography. The included studies on auditory function evaluated 1215 individuals in total, 714 PwMS and 501 healthy controls, the included studies on vestibular function covered 1128 individuals, 682 PwMS and 446 healthy controls. Included studies assessed participants who were over the age of 18, and the geographical location of individuals was not limited to a specific continent, country or hemisphere.

Neither meta-analyses nor effect size calculations could be performed due to the inconsistencies across studies in reporting results or in test stimulus and recording parameters that lead to the inability to combine their data (Supplement Tables S5, S6, S8, S9). This is a significant methodological limitation especially that the impact of this systematic review on clinical practice would have been much greater had quantitative analyses been performed.

For peer review only

Auditory system and MS

The specific effects of MS on the auditory system, based on results of auditory tests included in this systematic review, could not be established.

OAEs in PwMS

Effect of MS on the peripheral auditory system, as demonstrated by OAEs, was only found by one of the three included studies.[21] Di Mauro et al. (2019) reported abnormal OAEs with normal ABRs in participants newly diagnosed with RRMS, showing cochlear rather than

For peer review only - http://bmjopen.bmj.com/site/about/guidelines.xhtml

retrocochlear involvement.[21] They suggested that this cochlear dysfunction may be caused by a demyelinating process in the brainstem's medial olivocochlear bunch that innervates outer hair cells[68] resulting in decreased OAE amplitudes.[21] However, reduced OAE amplitudes were not reported by others, and it is unclear why Di Mauro et al. (2019) found differences between PwMS and controls, and both Kaytancı et al. (2016) and Saberi et al. (2012) did not.[14,47] Therefore, there is very limited evidence to date on the effects of MS on OAEs, and there is need for further investigations.

PTA in PwMS

Effects of MS on PTA thresholds could not be defined. Three of five included studies reported worse PTA thresholds in PwMS compared to controls.[14,18,48] However, Srinivasan et al. (2021) included only participants with normal hearing, i.e. test sample was biased towards better PTA,[48] therefore the difference between PwMS and controls, although statistically significant, is not clinically significant. Apart from Di Mauro et al. (2019), who found no difference between PwMS and controls but only included participants with normal hearing that were recently diagnosed with RRMS,[21] reasons behind differences in PTA results between studies are unclear. Type and duration of MS do not appear to be factors, as those who found a difference included participants with a mixture of MS types and MS durations. For example, Lewis et al. (2010) included participants (*n* = 47) with RRMS (mean MS duration: 12.6 years) and SPMS (mean MS duration: 23.3 years),[18] and Saberi et al. (2012) included PwMS (*n* = 60, MS type not reported) with a mean MS duration of 3.2 years,[14] and they both found differences in PTA thresholds between PwMS and controls. While Doty et al. (2012) did not find any difference in PTA thresholds with the inclusion of participants (*n* = 73) with different MS types (RRMS, PPMS, SPMS, Unspecified MS) and a mean MS duration of 7.36 years in males and 7.84 years in females.[19] Therefore, conclusions on the effects of MS on PTA thresholds cannot be reached given the limited literature and the inability to combine results for

For peer review only

For peer review only - http://bmjopen.bmj.com/site/about/guidelines.xhtml

a meta-analysis. Thus, there is a clear need for further studies investigating changes in PTA thresholds in PwMS.

ABRs in PwMS

The effects of MS on the brainstem auditory pathway as demonstrated by ABRs were more evident; 12 of 13 included studies found abnormalities in different ABR components in PwMS.[14,20,28,30,47–54] Demyelination plaques tend to occur in the brainstem, and the impairment of the auditory olivocochlear pathways by demyelination is possible.[6] Therefore, ABR abnormalities such as prolonged latencies are thought to be related to the effects of MS plaques in the brainstem on the integrity of the auditory pathways.[11] Abnormal ABR findings in PwMS reported by studies included in this review were consistent with previous literature.[25,69] Only Di Mauro et al. (2019) found no differences in ABRs between PwMS and controls; however, PwMS with hearing loss or brainstem lesions in their MRI were excluded from the study, plus the authors included only participants newly diagnosed with RRMS.[21] Matas et al. (2010) included normally hearing participants with RRMS (mean MS duration: 4.25 years) and found differences in ABRs between PwMS and controls.[49] Burina et al. (2008) also included participants with RRMS and brainstem or cerebellum lesions and found abnormal ABRs.[52] Therefore, the reasons Di Mauro et al. (2019) did not find differences in ABRs between PwMS and controls are likely related to the short duration of MS and the exclusion of participants with brainstem lesions. Although most studies that investigated ABRs in PwMS found abnormalities, the nature of these abnormalities could not be established. This is due to the differences between studies in reported ABR measures (e.g. absolute peak latencies versus peak amplitudes) and in stimulus and recording parameters (e.g. presentation levels, stimulus polarity, transducer). Therefore, there is a clear need for consistency across studies to better understand the effects of MS on ABRs.

For peer review only

CAEPs in PwMS

The effects of MS plaques on the integrity of the central auditory pathway as measured by CAEPs was much less studied, only two studies were included in this systematic review.[49,51] Both found abnormalities suggesting possible cortical involvement in PwMS; however, these studies could not be compared, and data could not be synthesised due to the use of different testing techniques. Therefore, further studies are needed to better understand the effects of MS on CAEPs.

Speech perception and temporal processing in PwMS

The effects of MS on speech perception and temporal processing were also infrequently studied, three studies were included in this systematic review.[18,22,23] Two of the three found abnormalities on WDS tests in PwMS, both studies that found abnormalities performed WDS testing in noise,[22,23] and the study that did not find abnormal WDS tested in quiet.[18] It is therefore likely that Lewis et al. (2010) did not find abnormal WDS in PwMS because they did not test in background noise.[18] This is supported by Valadbeigi et al.'s (2014) findings of worse WDS in noise than in quiet in PwMS.[22] These abnormalities in WDS are possibly related to MS lesions in the central auditory pathways. Any degeneration caused by MS plaques could lead to a misperception of the acoustic signal leading to poorer performance on WDS.[70] Additionally, the two studies that performed the GIN test found abnormalities in PwMS indicating lower temporal resolution performance than in controls.[22,23] A previous study suggested that persons with lesions in the central auditory system had worse temporal resolution performance and therefore poorer performance on the GIN test compared to healthy controls.[71] Therefore, it is possible that temporal resolution is affected by MS plaques in the CNS. Since the evidence regarding abnormalities in speech perception and temporal processing in PwMS was limited, this systematic review concludes that there is need for further research to evaluate MS's effect on functional auditory performance.

For peer review only

Vestibular System and MS

All studies included in this systematic review found abnormalities on vestibular tests suggesting that the vestibular system may be more affected by MS than the auditory system.

ENGs in PwMS

The two included studies that performed ENGs in PwMS found abnormalities suggestive of peripheral and/or central vestibular pathology.[27,55] Zeigelboim et al. (2008) primarily found peripheral vestibular pathology (83.4%) in their participants with RRMS,[55] while Degirmenci et al. (2010) mainly found central vestibular pathology (83.3%) in their participants with RRMS.[27] These contradictory results might be due to the inclusion of participants with RRMS in both studies, as persons with RRMS may differ in terms of lesion site in the CNS, severity of impairment, developmental stage of disease and frequency of attacks.[55] Given this limited and contradictory evidence, the effects of MS on ENGs are still unknown. Therefore, more literature is needed on different MS subtypes to better understand this.

Posturography in PwMS

The four included studies that investigated postural control using either static or dynamic posturography found abnormal posturography results in PwMS.[40,41,56,57] To maintain balance, the CNS evaluates the input from the vestibular, visual and somatosensory systems, then weighs the input according to where reliable information is coming from, and MS may affect each of these three systems.[56] Maintaining body balance in PwMS requires increasing the weight on the other two systems if one sensory system input is impaired. Yang and Liu (2020) concluded that PwMS rely mostly on cues from visual and proprioceptive systems to maintain their balance.[56] Lord et al. (1991) and Doty et al. (2018) also stated that PwMS had difficulty maintaining their balance when visual and proprioceptive feedback were inconsistent or conflicting.[57,72] In addition, a previous study reported that PwMS have slower somatosensory conduction than healthy individuals.[73] Thus, PwMS might receive impaired

For peer review only - http://bmjopen.bmj.com/site/about/guidelines.xhtml

or delayed proprioceptive feedback resulting in postural instability.[56,73,74] Another possible cause of instability in PwMS is that motor neurons might be affected by demyelination plaques, leading to an impairment in the transmission from the CNS to the motor end units.[56,75] Although, all four included studies found greater instability and impaired balance in PwMS, the evidence regarding the effects of MS on functional balance outcomes is very limited. Further research is required to make a clear conclusion on this subject.

VEMPs in PwMS

Thirteen of the 14 included studies that assessed VEMPs reported abnormalities in PwMS. Significant differences between PwMS and controls were reported by eight of the ten studies that compared the two groups.[30,35,50,58,60–63] Both Kavasoğlu et al. (2018) and Cochrane et al. (2021) did not find differences between PwMS and controls.[59,65] However, Kavasoğlu et al. (2018) found VEMP latency delays exceeding 2.5 SD from normative data in 23% of their PwMS;[65] Cochrane et al. (2021) did not report whether any VEMP abnormalities were found in their PwMS.[59] Kavasoğlu et al. (2018) included PwMS that were recently diagnosed (within less than one year), this may be the reason why they did not find differences between PwMS and controls.[65] Cochrane et al. (2021) attributed the lack of difference between PwMS and controls to the fact that their participants had low scores on the disability scale indicating that they were less likely to have lesions affecting VEMP results.[59] However, Gabelić et al. (2013) found differences between PwMS and controls and their participants had similar disability scale scores to Cochrane et al. (2021).[58] The remaining studies that reported percent PwMS that had abnormal VEMPs found various results. For example, Alpini et al. (2004) found abnormalities in 70 %[29] Versino et al. (2002) found abnormalities in 31.4 %[28] and Koura and Hussein (2018) found abnormalities in 100 % of PwMS who participated in their study.[66] The majority did not report type or duration of MS, or severity of impairment, therefore, it is difficult to speculate why some studies reported higher percentages of VEMP abnormalities in

For peer review only

For peer review only - http://bmjopen.bmj.com/site/about/guidelines.xhtml

PwMS than others. The most common reported cVEMP abnormalities were prolonged p13 and n23 latencies[28–30,35,58,61–66] and the most common reported oVEMP abnormalities were prolonged p1 and n1 latencies.[35,58] Prolonged latencies may be a result of inflammatory demyelination in the vestibular nuclei and the root entry zone of the eighth cranial nerve[7] leading to reduced neural transmission speed and consequently prolonged latencies. Additionally, Aidar and Suzuki (2005), and Koura and Hussein (2018) noted the absence of waves in 30 % and 40 % of PwMS,[62,66] respectively, which may be caused by a lesion in the vestibulospinal tract.[76] Although all studies showed VEMP abnormalities in PwMS, the nature of these abnormalities and their relationships to MS could not be established. This is due to the differences between studies in VEMP stimulus and recording parameters (e.g. presentation levels, stimulus polarity, transducer), resulting in the inability to combine data for a meta-analysis. In addition, type, duration, and severity of MS were not consistently reported. Therefore, there is a clear need for consistency across studies to better understand the effects of MS on the vestibular system by using VEMPs.

Rotary Chair testing in PwMS

The two studies that reported results on Rotary Chair testing from the same participants found differences that indicate central rather than peripheral vestibular impairments in PwMS compared to controls.[59,67] They suggested that central integration of peripheral input is what is impaired in PwMS.[59,67] However, more studies are needed to confirm these findings.

CONCLUSIONS

This systematic review aimed at understanding the effects of MS on the auditory and vestibular systems. No previous review that investigated this relationship was found. In this systematic review, an effect of MS on the auditory system was consistently found in the results of ABRs, CAEPs, WDS and GIN tests, but not in OAEs or PTAs. Indicating that the brainstem and central auditory systems were more likely to be affected by MS than the peripheral auditory system.

For peer review only

Furthermore, an effect of MS on the vestibular system was consistently found in the results of ENG, Dynamic and Static Posturography and VEMPs. This suggests that both the peripheral and central vestibular systems may be involved in the degenerative process of MS. However, quantitative analyses could not be performed in this systematic review due to inconsistencies across studies in reporting of results, in test procedures and in test stimulus and recording parameters. Therefore, although abnormal results in PwMS on both auditory and vestibular tests were identified, further studies are necessary to quantify this effect and obtain more robust evidence on the effects of MS on the auditory and vestibular systems. Future studies should use consistent test parameters and account for confounding factors such as type, duration and severity of MS.

CLINICAL IMPLICATIONS

This systematic review clearly demonstrates that audio-vestibular function is affected in PwMS. This suggests that tests of audio-vestibular function should be incorporated in the assessment protocols of PwMS. A test battery approach that includes tests of peripheral and central function would be most appropriate to ensure all potential regions that may be affected by MS plagues are evaluated. This review found that ABRs are the most sensitive measure of auditory function in PwMS; therefore, at least ABR testing must be included as part of the assessment of PwMS. Additionally, OAEs and PTA should also be included given that there is some evidence that the MS inflammatory process could affect the inner ear. For vestibular function, this review found that all tests are affected in PwMS suggesting that the vestibular test battery should be comprehensive and incorporate ENG/VNG, VEMPs, Rotary Chair, and Posturography to ensure that the whole vestibular pathway is evaluated.

Figure Legend:

Figure 1. Flow diagram of systematic literature review

For peer review only

Contributorship Statement: E.G and G.B equally contributed to this work (co-first authors). EG, G.B and K.K conceptualized, planned and designed this systematic review, E.G conducted the searches and initial screening of titles and abstracts. E.G and G.B independently reviewed full texts and conducted assessment of bias, E.G extracted data and this was verified by G.B, disagreements at any stage were resolved by discussion and by involvement of K.K.

E.G and G.B interpreted the results and this was reviewed by K.K.

E.G and G.B wrote the original manuscript and also reviewed and edited the manuscript. K.K was responsible for supervision, administration, and reviewing and editing the manuscript.

Funding: This research received no specific grant from any funding agency in the public, commercial or not-for-profit sectors. KK was supported by the NIHR Manchester Biomedical Research Centre, UK IS-BRC-1215-20007 and MRC Programme Grant MR/L003589/1.

Competing interest: No competing interests.

Data Sharing: No additional data available.

For peer review only

REFERENCES

1 Sospedra M, Martin R. Immunology of Multiple Sclerosis. *Annu Rev Immunol* 2005;**23**:683–747. doi:10.1146/annurev.immunol.23.021704.115707

2 Gold R, Linington C, Lassmann H. Understanding pathogenesis and therapy of multiple sclerosis via animal models: 70 years of merits and culprits in experimental autoimmune encephalomyelitis research. *Brain* 2006;**129**:1953–71. doi:10.1093/brain/awl075

3 Trapp BD, Peterson J, Ransohoff RM, *et al.* Axonal Transection in the Lesions of Multiple Sclerosis. *New Engl J Medicine* 1998;**338**:278–85. doi:10.1056/nejm199801293380502

4 Popescu BFGh, Lucchinetti CF. Pathology of Demyelinating Diseases. *Annu Rev Pathology Mech Dis* 2012;**7**:185–217. doi:10.1146/annurev-pathol-011811-132443

5 Rolak LA. Multiple Sclerosis: It's Not The Disease You Thought It Was. *Clin Medicine Res* 2003;**1**:57–60. doi:10.3121/cmr.1.1.57

6 Coelho A, erani B, Prasher D, *et al.* Auditory Efferent Function Is Affected in Multiple Sclerosis. *Ear Hearing* 2007;**28**:593–604. doi:10.1097/aud.0b013e31812f716e

7 Brandt T, Dieterich M. Preliminary Classification of Vestibular Brain-Stem Disorders. In: L.R. C, H.C. H, eds. *Brain-Stem Localization and Function*. Berlin, Heidelberg: : Springer 1993. 79–91. doi:10.1007/978-3-642-78172-8_9

8 Bogie JFJ, Stinissen P, Hendriks JJA. Macrophage subsets and microglia in multiple sclerosis. *Acta Neuropathol* 2014;**128**:191–213. doi:10.1007/s00401-014-1310-2

9 O'Malley JT, Nadol JB, McKenna MJ. Anti CD163+, Iba1+, and CD68+ Cells in the Adult Human Inner Ear. *Otol Neurotol* 2016;**37**:99–108. doi:10.1097/mao.0000000000000879

10 Di Stadio AD, Ralli M. Inner ear involvement in multiple sclerosis: An underestimated condition? *Mult Scler J* 2018;**24**:1264–5. doi:10.1177/1352458517750010

11 Di Stadio AD, Dipietro L, Ralli M, *et al.* Sudden hearing loss as an early detector of multiple sclerosis: a systematic review. *Eur Rev Med Pharmaco* 2018;**22**:4611–24. doi:10.26355/eurrev_201807_15520

12 Franklin DJ, Coker NJ, Jenkins HA. Sudden Sensorineural Hearing Loss as a Presentation of Multiple Sclerosis. *Archives Otolaryngology Head Neck Surg* 1989;**115**:41–5. doi:10.1001/archotol.1989.01860250043023

13 Drulović B, Ribarić-jankes K, Kostić V, *et al.* Multiple Sclerosis as the Cause of Sudden 'Pontine' Deafness. *Int J Audiol* 1994;**33**:195–201. doi:10.3109/00206099409071880

For peer review only

For peer review only - http://bmjopen.bmj.com/site/about/guidelines.xhtml

14 Saberi A, Hatamian HR, Nemati S, *et al.* Hearing statement in multiple sclerosis: a case control study using auditory brainstem responses and otoacoustic emissions. *Acta Medica Iranica* 2012;**50**:679–83.

15 Furst M, Levine RA. Hearing disorders in multiple sclerosis. *Handb Clin Neurology* 2015;**129**:649–65. doi:10.1016/b978-0-444-62630-1.00036-6

16 Dayal VS, Swisher LP. Pure tone thresholds in multiple sclerosis: A further study. *Laryngoscope* 1967;**77**:2169–77. doi:10.1288/00005537-196712000-00007

17 Cohen M, Rudge P. The Effect of Multiple Sclerosis on Pure Tone Thresholds. *Acta Oto-laryngol* 1984;**97**:291–5. doi:10.3109/00016488409130991

18 Lewis MS, Lilly DJ, Hutter MM, *et al.* Audiometric hearing status of individuals with and without multiple sclerosis. *J Rehabilitation Res Dev* 2010;**47**:669–78. doi:10.1682/jrrd.2010.01.0010

19 Doty RL, Tourbier I, Davis S, *et al.* Pure-Tone Auditory Thresholds Are Not Chronically Elevated in Multiple Sclerosis. *Behav Neurosci* 2012;**126**:314–24. doi:10.1037/a0027046

20 Lima TMA, Crato AN, Mancini PC, *et al.* Alterations in early auditory evoked potentials in multiple sclerosis patients. *Braz J Otorhinolar* 2009;**75**:177–81. doi:10.1016/s1808-8694(15)30775-8

21 Di Mauro RD, Di Girolamo SD, Ralli M, *et al.* Subclinical cochlear dysfunction in newly diagnosed relapsing-remitting multiple sclerosis. *Mult Scler Relat Dis* 2019;**33**:55–60. doi:10.1016/j.msard.2019.05.020

22 Valadbeigi A, Weisi F, Rohbakhsh N, *et al.* Central auditory processing and word discrimination in patients with multiple sclerosis. *Eur Arch Oto-rhino-l* 2014;**271**:2891–6. doi:10.1007/s00405-013-2776-6

23 Elbeltagy R, Gad N, Ismail M. Central auditory function in multiple sclerosis patients. *Indian J Otology* 2019;**25**:90–6. doi:10.4103/indianjotol.indianjotol_80_18

24 Noffsinger D, Olsen WO, Carhart R, *et al.* Auditory and vestibular aberrations in multiple sclerosis. *Acta Oto-laryngologica Suppl* 1972;**303**:1–63.

25 Grénman R. Involvement of the audiovestibular system in multiple sclerosis. An otoneurologic and audiologic study. *Acta Oto-laryngologica Suppl* 1985;**420**:1–95.

26 Cipparrone L, Fratiglioni L, Siracusa G, *et al.* Electronystagmography in the diagnosis of multiple sclerosis. *Acta Neurol Scand* 1989;**80**:193–200. doi:10.1111/j.1600-0404.1989.tb03862.x

27 Degirmenci E, Bir LS, Ardic FN. Clinical and electronystagmographical evaluation of vestibular symptoms in relapsing remitting multiple sclerosis. *Neurol Res* 2010;**32**:986–91. doi:10.1179/016164110x12681290831405

For peer review only

For peer review only - http://bmjopen.bmj.com/site/about/guidelines.xhtml

28 Versino M, Colnaghi S, Callieco R, *et al.* Vestibular evoked myogenic potentials in multiple sclerosis patients. *Clin Neurophysiol* 2002;**113**:1464–9. doi:10.1016/s1388-2457(02)00155-4

29 Alpini D, Pugnetti L, Caputo D, *et al.* Vestibular evoked myogenic potentials in multiple sclerosis: clinical and imaging correlations. *Mult Scler* 2004;**10**:316–21. doi:10.1191/1352458504ms1041oa

30 Eleftheriadou A, Deftereos SN, Zarikas V, *et al.* The diagnostic value of earlier and later components of Vestibular Evoked Myogenic Potentials (VEMP) in Multiple Sclerosis. *J Vestib Res* 2009;**19**:59–66. doi:10.3233/ves-2009-0342

31 Gazioglu S, Boz C. Ocular and cervical vestibular evoked myogenic potentials in multiple sclerosis patients. *Clin Neurophysiol* 2012;**123**:1872–9. doi:10.1016/j.clinph.2012.01.022

32 Güven H, Bayır Ö, Aytaç E, *et al.* Vestibular-evoked myogenic potentials, clinical evaluation, and imaging findings in multiple sclerosis. *Neurol Sci* 2014;**35**:221–6. doi:10.1007/s10072-013-1483-9

33 Crnošija L, Skorić MK, Gabelić T, *et al.* Correlation of the VEMP score, ambulation and upper extremity function in clinically isolated syndrome. *J Neurol Sci* 2015;**359**:197–201. doi:10.1016/j.jns.2015.10.049

34 Gabelić T, Skorić MK, Adamec I, *et al.* The vestibular evoked myogenic potentials (VEMP) score: a promising tool for evaluation of brainstem involvement in multiple sclerosis. *Eur J Neurol* 2015;**22**:261-e21. doi:10.1111/ene.12557

35 Parsa MS, Mohammadkhani G, Hajabolhassani F, *et al.* Cervical and ocular vestibular evoked myogenic potentials in multiple sclerosis participants. *Medical J Islamic Repub Iran* 2015;**29**:164.

36 Crnošija L, Skorić MK, Gabelić T, *et al.* Vestibular evoked myogenic potentials and MRI in early multiple sclerosis: Validation of the VEMP score. *J Neurol Sci* 2017;**372**:28–32. doi:10.1016/j.jns.2016.11.028

37 Di Stadio AD, Dipietro L, Ralli M, *et al.* The role of vestibular evoked myogenic potentials in multiple sclerosis-related vertigo. A systematic review of the literature. *Mult Scler Relat Dis* 2019;**28**:159–64. doi:10.1016/j.msard.2018.12.031

38 Shimizu K, Murofushi T, Sakurai M, *et al.* Vestibular evoked myogenic potentials in multiple sclerosis. *J Neurology Neurosurg Psychiatry* 2000;**69**:276–7. doi:10.1136/jnnp.69.2.276

39 Murofushi T, Shimizu K, Takegoshi H, *et al.* Diagnostic Value of Prolonged Latencies in the Vestibular Evoked Myogenic Potential. *Archives Otolaryngology Head Neck Surg* 2001;**127**:1069–72. doi:10.1001/archotol.127.9.1069

40 Inojosa H, Schriefer D, Klöditz A, *et al.* Balance Testing in Multiple Sclerosis—Improving Neurological Assessment With Static Posturography? *Front Neurol* 2020;**11**:135. doi:10.3389/fneur.2020.00135

For peer review only

For peer review only - http://bmjopen.bmj.com/site/about/guidelines.xhtml

41 Inojosa H, Schriefer D, Trentzsch K, *et al.* Visual Feedback and Postural Control in Multiple Sclerosis. *J Clin Medicine* 2020;**9**:1291. doi:10.3390/jcm9051291

42 Prosperini L, Petsas N, Raz E, *et al.* Balance deficit with opened or closed eyes reveals involvement of different structures of the central nervous system in multiple sclerosis. *Mult Scler J* 2014;**20**:81–90. doi:10.1177/1352458513490546

43 Page MJ, McKenzie JE, Bossuyt PM, *et al.* The PRISMA 2020 statement: an updated guideline for reporting systematic reviews. *Bmj* 2021;**372**:n71. doi:10.1136/bmj.n71

44 Kim SY, Park JE, Lee YJ, *et al.* Testing a tool for assessing the risk of bias for nonrandomized studies showed moderate reliability and promising validity. *J Clin Epidemiol* 2013;**66**:408–14. doi:10.1016/j.jclinepi.2012.09.016

45 Wells G, Shea B, O'Connell D, *et al.* The Newcastle-Ottawa Scale (NOS) for assessing the quality of nonrandomised studies in meta-analyses. Ottawa: Ottawa Hospital Research Institute. http://www.ohri.ca/programs/clinical_epidemiology/oxford.asp (accessed 2 Jul 2020).

46 Moskalewicz A, Oremus M. No clear choice between Newcastle–Ottawa Scale and Appraisal Tool for Cross-Sectional Studies to assess methodological quality in cross-sectional studies of health-related quality of life and breast cancer. *J Clin Epidemiol* 2020;**120**:94–103. doi:10.1016/j.jclinepi.2019.12.013

47 Kaytancı E, Ozdamar OI, Acar GO, *et al.* Evaluation of transiently evoked otoacoustic emissions and auditory brainstem responses in patients with multiple sclerosis. *Ear Nose Throat J* 2016;**95**:E12–7.

48 Srinivasan VS, Krishna R, Munirathinam BR. Effectiveness of Brainstem Auditory Evoked Potentials Scoring in Evaluating Brainstem Dysfunction and Disability Among Individuals With Multiple Sclerosis. *Am J Audiol* 2021;**30**:255–65. doi:10.1044/2020_aja-20-00155

49 Matas CG, Matas SL de A, Oliveira CRS de, *et al.* Auditory evoked potentials and multiple sclerosis. *Arq Neuro-psiquiat* 2010;**68**:528–34. doi:10.1590/s0004-282x2010000400010

50 Delphi M, Sayaf M, Taheri F, *et al.* Audio and vestibular analyses in patient with multiple sclerosis: a case–control study. *Hear Balance Commun* 2021;**19**:1–6. doi:10.1080/21695717.2021.1933318

51 Japaridze G, Shakarishvili R, Kevanishvili Z. Auditory brainstem, middle-latency, and slow cortical responses in multiple sclerosis. *Acta Neurol Scand* 2002;**106**:47–53. doi:10.1034/j.1600-0404.2002.01226.x

52 Burina A, Sinanović O, Smajlović D, *et al.* Some Aspects of Balance Disorder in Patients with Multiple Sclerosis. *Bosnian J Basic Med* 2008;**8**:80–5. doi:10.17305/bjbms.2008.3003

For peer review only - http://bmjopen.bmj.com/site/about/guidelines.xhtml

53 Pokryszko-Dragan A, Bilinska M, Gruszka E, *et al.* Assessment of visual and auditory evoked potentials in multiple sclerosis patients with and without fatigue. *Neurol Sci* 2015;**36**:235–42. doi:10.1007/s10072-014-1953-8

54 Rishiq D, Harkrider A, Springer C, *et al.* Click-evoked and speech-evoked auditory brainstem responses from individuals with multiple sclerosis. *Neurosci Lett* 2021;**740**:135460. doi:10.1016/j.neulet.2020.135460

55 Zeigelboim BS, Arruda WO, Mangabeira-Albernaz PL, *et al.* Vestibular findings in relapsing, remitting multiple sclerosis: a study of thirty patients. *Int Tinnitus J* 2008;**14**:139–45.

56 Yang F, Liu X. Relative importance of vision and proprioception in maintaining standing balance in people with multiple sclerosis. *Mult Scler Relat Dis* 2020;**39**:101901. doi:10.1016/j.msard.2019.101901

57 Doty RL, MacGillivray MR, Talab H, *et al.* Balance in multiple sclerosis: relationship to central brain regions. *Exp Brain Res* 2018;**236**:2739–50. doi:10.1007/s00221-018-5332-1

58 Gabeli T, Krbot M, Šefer AB, *et al.* Ocular and Cervical Vestibular Evoked Myogenic Potentials in Patients With Multiple Sclerosis. *J Clin Neurophysiol* 2013;**30**:86–91. doi:10.1097/wnp.0b013e31827eda0c

59 Cochrane GD, Christy JB, Motl RW. Comprehensive Clinical Assessment of Vestibular Function in Multiple Sclerosis. *J Neurol Phys Ther* 2021;**45**:228–34. doi:10.1097/npt.0000000000000358

60 Elmoazen D, Kozou H, Gawies M, *et al.* Assessment of Cervical and Ocular Vestibular Evoked Myogenic Potentials in Multiple Sclerosis PatientsS. *Egyptian Journal of Ear, Nose, Throat and Allied Sciences* 2021;**22**:1–9. doi:10.21608/ejentas.2020.44108.1262

61 Sartucci F, Logi F. Vestibular-evoked myogenic potentials: A method to assess vestibulo-spinal conduction in multiple sclerosis patients. *Brain Res Bull* 2002;**59**:59–63. doi:10.1016/s0361-9230(02)00842-0

62 Aidar RC, Suzuki FA. Vestibular evoked myogenic potential: new perspectives in multiple sclerosis. *Braz J Otorhinolar* 2005;**71**:48–54. doi:10.1016/s1808-8694(15)31284-2

63 Patkó T, Simó M, Arányi Z. Vestibular click-evoked myogenic potentials: sensitivity and factors determining abnormality in patients with multiple sclerosis. *Mult Scler J* 2007;**13**:193–8. doi:10.1177/1352458506070940

64 Harirchian MH, Karimi N, Nafisi S, *et al.* Vestibular evoked myogenic potential for diagnoses of multiple sclerosis: is it beneficial? *Medicinski Glasnik Official Publ Medical Assoc Zenica-doboj Canton Bosnia Herzegovina* 2013;**10**:321–6.

65 Kavasoğlu G, Gökçay F, Yüceyar N, *et al.* Cervical vestibular-evoked myogenic potentials in patients with multiple sclerosis: sensitive in detecting brainstem involvement? *Neurol Sci* 2018;**39**:365–71. doi:10.1007/s10072-017-3215-z

For peer review only - http://bmjopen.bmj.com/site/about/guidelines.xhtml

66 Koura R, Hussein M. Vestibular-evoked myogenic potential: an easy neurophysiological tool for evaluating brain stem involvement in multiple sclerosis. *Egypt J Otolaryngology* 2018;**34**:144–8. doi:10.4103/ejo.ejo_73_17

67 Cochrane GD, Christy JB, Sandroff BM, *et al.* Cognitive and Central Vestibular Functions Correlate in People With Multiple Sclerosis. *Neurorehab Neural Re* 2021;**35**:1030–8. doi:10.1177/15459683211046268

68 Veuillet E, Khalfa S, Collet L. Clinical relevance of medial efferent auditory pathways. *Scand Audiology Suppl* 1999;**51**:53–62.

69 Protti-Patterson E, Young ML. The use of subjective and objective audiologic test procedures in the diagnosis of multiple sclerosis. *Otolaryng Clin N Am* 1985;**18**:241–55.

70 Magnano I, Aiello I, Piras MR. Cognitive impairment and neurophysiological correlates in MS. *J Neurol Sci* 2006;**245**:117–22. doi:10.1016/j.jns.2005.08.027

71 Musiek FE, Shinn JB, Jirsa R, *et al.* GIN (Gaps-In-Noise) Test Performance in Subjects with Confirmed Central Auditory Nervous System Involvement. *Ear Hearing* 2005;**26**:608–18. doi:10.1097/01.aud.0000188069.80699.41

72 Lord SR, Clark RD, Webster IW. Postural Stability and Associated Physiological Factors in a Population of Aged Persons. *J Gerontology* 1991;**46**:M69–76. doi:10.1093/geronj/46.3.m69

73 Cameron MH, Lord S. Postural Control in Multiple Sclerosis: Implications for Fall Prevention. *Curr Neurol Neurosci* 2010;**10**:407–12. doi:10.1007/s11910-010-0128-0

74 Cameron MH, Horak FB, Herndon RR, *et al.* Imbalance in multiple sclerosis: A result of slowed spinal somatosensory conduction. *Somatosens Mot Res* 2008;**25**:113–22. doi:10.1080/08990220802131127

75 Cattaneo D, Jonsdottir J. Sensory impairments in quiet standing in subjects with multiple sclerosis. *Mult Scler* 2009;**15**:59–67. doi:10.1177/1352458508096874

76 Colebatch JG, Halmagyi GM, Skuse NF. Myogenic potentials generated by a click-evoked vestibulocollic reflex. *J Neurology Neurosurg Psychiatry* 1994;**57**:190–7. doi:10.1136/jnnp.57.2.190

For peer review only - http://bmjopen.bmj.com/site/about/guidelines.xhtml

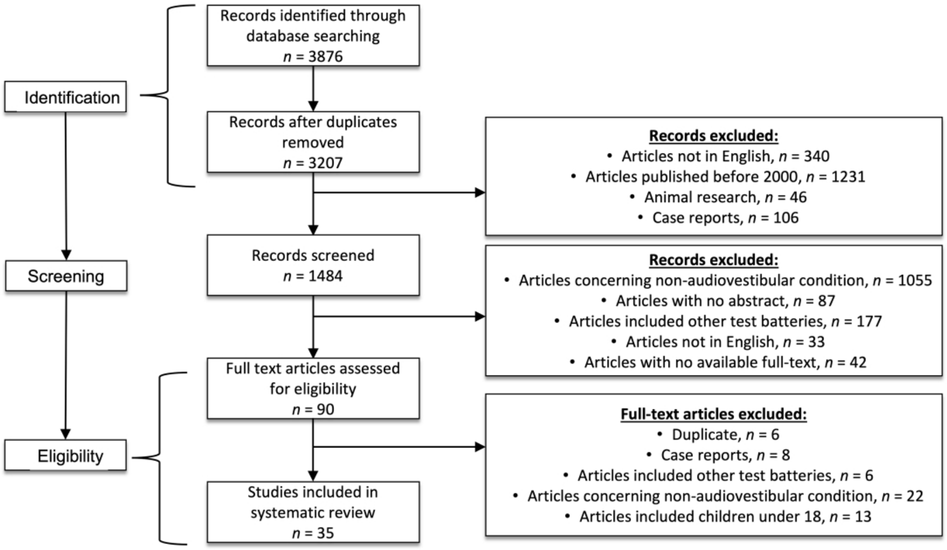

Figure 1. Flow diagram of systematic literature review

Supplement

Section I: Literature Search Strategy

Database searched: **Scopus**

Date of coverage: **2000 to November 2021**

Search undertaken: **August 2020, September 2020, February 2021, November 2021**

((TITLE-ABS-KEY("multiple sclerosis")AND (hearing OR auditory OR "hearing loss" OR "auditory function" OR “hearing dysfunction”)) OR (TITLE-ABS-KEY("multiple sclerosis") AND (dizziness OR vestibular OR vertigo OR equilibrium OR balance OR balanced OR “vestibular function”)

For peer review only

Database searched: Pubmed

Date of coverage: 2000 to November 2021

Search undertaken: August 2020, September 2020, February 2021, November 2021

(("multiple sclerosis"[MeSH Terms] AND ("auditorially"[All Fields] OR "auditory"[All Fields] OR ("hearing"[MeSH Terms] OR "hearing"[All Fields] OR "hearings"[All Fields]) OR "hearing loss"[All Fields] OR "hearing" [All Fields] OR "auditory function"[All Fields]) OR ("multiple sclerosis"[MeSH Terms] AND ("vestibular"[All Fields] OR ("dizziness"[MeSH Terms] OR "dizziness"[All Fields] OR "dizzy"[All Fields] OR "vertigo"[MeSH Terms] OR "vertigo"[All Fields]) OR ("vertigo"[MeSH Terms] OR "vertigo"[All Fields] OR "vertigos"[All Fields] OR "vertigoes"[All Fields]) OR ("equilibrium"[All Fields] OR "equilibriums"[All Fields]) OR ("balance"[All Fields] OR "balanced"[All Fields] OR "balances"[All Fields] OR "balancing"[All Fields]) OR "vestibular function"[All Fields]) AND

Translations

Multiple Sclerosis[MeSH Terms]: "multiple sclerosis"[MeSH Terms]

auditory: "auditorially"[All Fields] OR "auditory"[All Fields]

hearing: "hearing"[MeSH Terms] OR "hearing"[All Fields] OR "hearings"[All Fields]

multiple sclerosis[MeSH Terms]: "multiple sclerosis"[MeSH Terms]

dizziness: "dizziness"[MeSH Terms] OR "dizziness"[All Fields] OR "dizzy"[All Fields] OR "vertigo"[MeSH Terms] OR "vertigo"[All Fields]

vertigo: "vertigo"[MeSH Terms] OR "vertigo"[All Fields] OR "vertigos"[All Fields] OR "vertigoes"[All Fields]

equilibrium: "equilibrium"[All Fields] OR "equilibriums"[All Fields]

balance: "balance"[All Fields] OR "balanced"[All Fields] OR "balances"[All Fields] OR "balancing"[All Fields]

For peer review only - http://bmjopen.bmj.com/site/about/guidelines.xhtml

Database searched: Web of Science

Date of coverage: 2000 to November 2021

Search undertaken: August 2020, September 2020, February 2021, November 2021

Databases= WOS, BCI, BIOSIS, CCC, DRCI, DIIDW, KJD, MEDLINE, RSCI, SCIELO, ZOOREC, Timespan=2000-2021

#1 TS=("multiple sclerosis")

#2 TS=(auditory OR audio OR "auditory function" OR “auditory system”)

#3 TS=(hear* OR hearing OR "hearing loss")

#4 #2 OR #3

#5 #4 AND #1

#6 TS=(vestibular OR vestibul* OR "vestibular dysfunction" OR "vestibular system")

#7 TS=(vertigo)

#8 TS=(dizzy OR dizziness)

#9 TS=(balance OR balanced OR balances OR balanc*)

#10 #6 OR #7 OR #8 OR #9

#11 #1 AND #10

#12 #5 OR #11 AND

For peer review only

Database searched: NICE

Date of coverage: 2000 to November 2021

Search undertaken: August 2020, September 2020, February 2021, November 2021

(("multiple sclerosis") AND (hearing OR auditory OR "hearing loss" OR "auditory function" OR hear* OR “hearing dysfunction)) OR (("multiple sclerosis") AND (dizziness OR vestibular OR vertigo OR equilibrium OR balance OR balanced OR “vestibular function”))

For peer review only - http://bmjopen.bmj.com/site/about/guidelines.xhtml

Section II: Assessment of Bias

Table S1: *Assessment of Bias for case-control studies according to RoBANs*

Year	Author	Selection of participants	Confounding Variables	Measurement of Exposure	Blinding of the Outcome of the Assessment	Incomplete Outcome of Data	Selective Outcome Reporting
2002	Japaridze et al.[1]	?	+	+	-	+	+
2002	Sartucci and Logi[2]	+	+	+	-	+	+
2004	Alpini et al.[3]	-	?	+	-	+	+
2005	Aidar and Suzuki[4]	+	+	+	-	+	-
2007	Patkó et al.[5]	?	-	+	-	+	+
2008	Zeigelboim et al.[6]	?	+	+	-	+	+
2009	Lima et al.[7]	?	?	+	-	+	+
2010	Lewis et al.[8]	+	+	+	-	+	+
2010	Matas et al.[9]	+	+	+	-	+	+
2012	Doty et al.[10]	-	+	+	-	+	+
2012	Saberi et al.[11]	+	+	+	-	+	+
2013	Gabelić et al.[12]	+	+	+	-	+	+
2013	Harirchian et al.[13]	+	+	+	-	+	+
2014	Valadbeigi et al.[14]	+	+	+	-	+	+
2015	Pokryszko-Dragan et al.[15]	+	+	+	-	+	+
2018	Doty et al.[16]	-	+	+	-	+	+
2018	Kavasoğlu et al.[17]	+	?	+	-	+	+
2018	Koura et al.[18]	+	+	+	-	+	+
2019	Elbeltagy et al.[19]	-	+	+	-	+	+
2021	Rishiq et al.[20]	?	+	+	-	+	+
2021	Srinivasan et al.[21]	+	+	+	-	+	+
2021	Elmoazen et al.[22]	+	+	+	-	+	+

Green "+" = low risk of bias, red "-" = high risk of bias, yellow "?" = unclear risk of bias.

Table S2: *Risk of bias assessment of cohort studies by the Newcastle-Ottawa Quality Assessment form for cohort studies.*

Year	Author	Representativeness of exposed cohort	Selection of non-exposed cohort	Ascertainment of exposure	Outcome of interest not present at the beginning	Comparability of cohorts/confounders	Assessment of the outcome	Follow-up Length	Adequacy of follow-up of cohorts	Study quality
2002	Versino et al.[23]	*	***n/a***	*	*	***n/a***	-	***n/a***	*	**?**
2008	Burina et al.[24]	-	***n/a***	*	***n/a***	-	-	-	-	**?**
2009	Eleftheriadou et al.[25]	*	-	*	*	*	-	*	*	**Poor**
2010	Degirmenci et al.[26]	*	-	*	*	*	*	*	*	**Good**
2016	Kaytancı et al.[27]	-	-	*	-	*	-	*	*	**Poor**
2019	Di Mauro et al.[28]	*	-	*	*	*	-	*	*	**Good**

'' = low risk of bias, '-' = high risk of bias, "n/a" = the criteria does not apply to the paper, "?" = unknown quality of the paper due to the presence of "n/a" criteria.*

Table S3: *Risk of bias assessment of cross-sectional studies by the Newcastle-Ottawa Quality Assessment form which is adapted for cross-sectional studies.*

Year	Author	Representativeness of the sample	Sample Size	Non-respondents	Ascertainment of exposure (risk factor)	Comparability of groups/confounders	Assessment of the outcome	Statistical test	Study quality
2015	Parsa et al.[29]	-	-	-	*	*	-	*	**Poor**
2020	Yang and Liu[30]	*	-	*	*	*	-	*	**Good**
2020	Inojosa et al.[31]	*	-	*	*	*	-	*	**Good**
2020	Inojosa et al.[32]	*	-	*	*	*	-	*	**Good**
2021	Delphi et al.[33]	-	-	*	*	*	-	*	**Good**
2021	Cochrane et al.[34]	*	-	*	*	*	-	*	**Good**
2021	Cochrane et al.[35]	*	-	*	*	*	-	*	**Good**

'' = low risk of bias, '-' = high risk of bias.*

Section III: Multiple Sclerosis and Auditory Function

Table S4. Characteristics of studies examining the effects of MS on auditory function

Test Battery	*Author (Year)*	*Study Type*	*Sample Size*		*PwMS Mean Age (years)*	*PwMS Age range (Years)*	*% Female*	*MS Type (sample size)*	*MS Duration Mean (range) years*	*Results of PwMS compared to controls / normative data*
			PwMS	*Controls*						
ABR	Japaridze et al. (2002)[1]	Case-Control	40	33	30.5	18-57	77.5	?	?	65% Abnormal
	Versino et al. (2002)[23]	Cohort	65	18	35.5	19-61	?	?	?	37.5% Abnormal
	Burina et al. (2008)[24]	Cohort	60	NA	37.2	?	68.3	RRMS	?	95% Abnormal
	Eleftheriadou et al. (2009)[25]	Cohort	46	40	40	20-66	45.6	RRMS	4.6	26% Abnormal
	Lima et al. (2009)[7]	Case-control	25	NA	42.6 (female)<br>38 (male)	33-53 (female)<br>24-56 (male)	64.0	RRMS (14), PPMS (8), Unspecified Progressive (3)	?	30% Abnormal
	Matas et al. (2010)[9]	Case-control	25	25	34.88	25-55	76.0	RRMS	4.25 (?)	Significantly different
	Saberi et al. (2012)[11]	Case-control	60	38	29.9	?	73.3	?	3.2 (?)	20% Abnormal
	Pokryszko-Dragan et al. (2015)[15]	Case-control	86	40	39.55	19-60	72.0	?	8.57 (1-30)	Significantly different
	Kaytancı et al. (2016)[27]	Cohort	20	20	31.3	?	55.0	?	?	Significantly different
	Di Mauro et al. (2019)[28]	Cohort	40	40	37	18-50	60.0	RRMS	0.8 (0.8-2)	No difference
	Delphi et al. (2021)[33]	Cross-sectional	25	25	31.43	18-45	72.0	?	?	Significantly different
	Rishiq et al. (2021)[20]	Case-control	11	9	49.5	34-68	72.7	RRMS (9), PPMS (1), SPMS (1)	11.9 (2-30)	Significantly different at high click rate
	Srinivasan et al. (2021)[21]	Case-control	45	45	31.77	18-50	73.3	RRMS	6.1 (5.37)	Significantly different and 55.56 % abnormal
PTA	Lewis et al. (2010)[8]	Case-control	47	49	51.4	21-65	57.7 RRMS, 28.6 SPMS	RRMS (26), SPMS (21)	RRMS: 12.6 (2-43)<br>SPMS: 23.3 (8-50)	Worse thresholds
	Doty et al. (2012)[10]	Case-control	73	73	Males: 45.24<br>Females: 45.6	?	71.2	RRMS (57), PPMS (3), SPMS (6), Unspecified (7)	Males: 7.36 (?)<br>Females: 7.84 (?)	No significant difference
	Saberi et al. (2012)[11]	Case-control	60	38	29.9	?	73.3	?	3.2 (?)	Worse thresholds in female PwMS
	Di Mauro et al. (2019)[28]	Cohort	40	40	37	18-50	60.0	RRMS	0.8 (0.8-2)	No significant difference
	Srinivasan et al. (2021)[21]	Case-control	45	45	31.77	18-50	73.3	RRMS	6.1 (5.37)	Significantly different
TEOAEs & DPOAEs	Saberi et al. (2012)[11]	Case-control	60	38	29.9	?	73.3	?	3.2 (?)	No significant difference
	Kaytancı et al. (2016)[27]	Cohort	20	20	31.3	?	55.0	?	?	No significant difference
	Di Mauro et al. (2019)[28]	Cohort	40	40	37	18-50	60.0	RRMS	0.8 (0.8-2)	Significantly lower

For peer review only

Table S4 continued: Characteristics of studies examining the effects of MS on auditory function

Test Battery	Author (Year)	Study Type	Sample Size		PwMS Mean Age (years)	PwMS Age range (Years)	% Female	MS Type (sample size)	MS Duration Mean (range) years	Results of PwMS compared to controls / normative data
			PwMS	Controls						
WDS	Lewis et al. (2010)[8]	Case-control	47	49	51.4	21-65	57.7 RRMS, 28.6 SPMS	RRMS (26), SPMS (21)	RRMS: 12.6 (2-43) SPMS: 23.3 (8-50)	No significant difference
	Valadbeigi et al. (2014)[[14]	Case-control	26	26	28.9	18-40	?	RRMS	?	Lower scores PwMS
	Elbeltagy et al. (2019)[19]	Case-control	20	20	37.6	30-50	?	RRMS	?	Significantly lower
DPST	Valadbeigi et al. (2014)[14]	Case-control	26	26	28.9	18-40	?	RRMS	?	Significantly different
GIN	Valadbeigi et al. (2014)[14]	Case-control	26	26	28.9	18-40	?	RRMS	?	Significantly different
	Elbeltagy et al. (2019)[19]	Case-control	20	20	37.6	30-50	?	RRMS	?	Significantly different
CAEP	Japaridze et al. (2002)[1]	Case-Control	40	33	30.5	18-57	77.5	?	?	30% Abnormal
	Matas et al. (2010)[9]	Case-control	25	25	34.88	25-55	76.0	RRMS	4.25 (?)	16% Abnormal

Abbreviations: PTA= Pure Tone Audiometry, ABR= Auditory Brainstem Response, OAE= Oto-acoustic Emissions, TEOAE= Transient Evoked Oto acoustic emission, DPOAE= Distortion Product Oto acoustic Emissions, CAEP= Cortical Auditory Evoked Potentials, SCP= Slow Cortical Potentials, GIN= Gaps in Noise test, WDS= Word Discrimination Scores, DPST= Duration pattern sequence test, MS= Multiple Sclerosis, PwMS = Persons with Multiple Sclerosis, ? = not reported.

Cells shaded in grey represent test results that were abnormal or showed a difference between PwMS and Controls

For peer review only - http://bmjopen.bmj.com/site/about/guidelines.xhtml

Extracted Pure Tone Audiometry Thresholds and Auditory Brainstem Response Results

Table S5: Pure Tone Audiometry Thresholds

Author (Year)	Group	Test Ear	PTA Threshold Per Frequency (Hz)													
			125		250		500		1000		2000		4000		8000	
			$\bar{x}$	SD	$\bar{x}$	SD	$\bar{x}$	SD	$\bar{x}$	SD	$\bar{x}$	SD	$\bar{x}$	SD	$\bar{x}$	SD
Doty et al. (2012)[10]	Male PwMS	Average Across Ears	Not Tested		15.87	5.39	14.05	5.94	12.52	7.43	12.79	8.97	20.03	11.71	17.02	17.26
	Male Controls				14.35	8.24	15.90	8.68	15.53	8.58	17.74	8.93	22.77	16.08	20.23	22.49
	Female PwMS				12.85	6.88	12.49	6.50	12.05	6.59	14.62	7.19	15.65	8.97	13.28	12.38
	Female Controls				11.28	7.77	12.52	7.60	10.45	6.82	11.62	8.17	13.92	8.79	12.65	12.60
Di Mauro et al. (2019)[28]	PwMS	Right	10.30	1.60	10.10	0.08	10.10	0.08	10.40	1.10	11.10	1.70	10.80	1.80	13.00	2.60
	Controls	Right	10.90	1.40	10.80	1.60	11.00	1.90	10.80	2.30	11.00	1.20	11.90	2.40	13.90	2.40
	PwMS	Left	10.30	1.70	10.90	1.20	10.40	1.90	10.40	1.30	10.80	1.10	11.40	1.70	12.50	2.20
	Controls	Left	10.90	1.40	10.80	1.60	11.00	1.30	10.90	2.10	11.10	1.90	11.90	2.80	13.90	2.40
Srinivasan et al. (2021)[21]	PwMS	Average Across Ears	Not Tested		12.22	6.40	13.11	5.92	13.61	5.90	12.55	7.19	13.66	8.06	16.16	9.08
	Controls				8.94	5.05	9.50	4.17	9.88	3.82	9.50	5.45	10.88	5.83	12.05	5.89

Abbreviations:
PwMS = Persons with Multiple Sclerosis, PTA = Pure Tone Audiometry, $\bar{x}$ = Mean, SD = Standard Deviation

For peer review only

Table S6: Auditory Brainstem Response Parameters and Results

Author (Year)	ABR Parameters							Peak Latency (ms)						Interpeak Latencies (ms)						Peak Amplitude (µV)			
	Click Polarity - Transducer	PR	PL	Ave.	BP Filter	Group	Test Ear	I		III		V		I-III		III-V		I-V		I		V	
								$\bar{x}$	SD	$\bar{x}$	SD	$\bar{x}$	SD	$\bar{x}$	SD	$\bar{x}$	SD	$\bar{x}$	SD	$\bar{x}$	SD	$\bar{x}$	SD
Lima et al. (2009)[7]	Rarefaction - TDH 39 Headphone	13.10	60 dB SL*	1024	100-3000	PwMS	Right	1.50	0.16	3.63	0.30	5.56	0.40	2.13	0.22	1.93	0.31	4.06	0.33	?	?	?	?
							Left	1.53	0.20	3.63	0.30	5.64	0.43	2.10	0.31	2.01	0.29	4.11	0.39	?	?	?	?
Matas et al. (2010)[9]	Rarefaction - TDH 39 Headphone	19.90	80 dB nHL	2000	100-3000	PwMS	Average Across Ears	1.53	0.13	3.72	0.31	5.78	0.37	2.21	0.26	2.03	0.27	4.26	0.35	?	?	?	?
						Controls		1.53	0.07	3.57	0.09	5.50	0.45	2.04	0.06	1.98	0.08	4.03	0.06	?	?	?	?
Pokryszko-Dragan et al. (2015)[15]	? - Earphone unspecified	20.30	65 dB SL*	200	150-3000	PwMS	Right	1.72	0.19	3.88	0.33	5.81	0.81	2.18	0.19	2.00	0.30	4.19	0.39	0.29	0.18	0.37	0.20
						Controls		1.70	0.14	3.85	0.16	5.73	0.23	2.14	0.12	1.87	0.18	4.02	0.20	0.30	0.10	0.46	0.19
						PwMS	Left	1.68	0.12	3.87	0.17	5.77	0.73	2.19	0.16	1.96	0.30	4.15	0.34	0.32	0.14	0.40	0.24
						Controls		1.69	0.13	3.83	0.12	5.70	0.21	2.13	0.14	1.87	0.17	4.00	0.19	0.30	0.14	0.44	0.16
Kaytancı et al. (2016)[27]	? - TDH 39 Headphone	13.00	70 dB nHL	1500	2000-4000	PwMS	Right	2.03	0.27	4.36	0.49	6.26	0.35	2.33	0.49	1.90	0.28	4.23	0.42	?	?	?	?
						Controls		1.45	0.30	3.52	0.31	5.38	0.53	2.07	0.44	1.85	0.48	3.92	0.53	?	?	?	?
						PwMS	Left	1.86	0.24	4.41	0.39	6.25	0.43	2.54	0.34	1.84	0.20	4.38	0.43	?	?	?	?
						Controls		1.47	0.24	3.40	0.43	5.39	0.46	1.92	0.48	1.99	0.53	3.91	0.47	?	?	?	?
						PwMS	IA Diff.	?	?	?	?	0.40	0.46	0.35	0.24	0.49	0.46	0.40	0.48	?	?	?	?
						Controls		?	?	?	?	0.15	0.12	0.34	0.35	0.27	0.30	0.19	0.16	?	?	?	?
Delphi et al. (2021)[33]	Rarefaction - ?	9.00	70 dB nHL	?	30-3000	PwMS	Right	1.89	0.11	4.25	0.14	6.32	0.13	2.30	0.11	2.21	0.09	4.48	0.13	?	?	?	?
						Controls		1.87	0.14	4.01	0.13	5.79	0.14	2.17	0.09	1.99	0.13	4.12	0.11	?	?	?	?
						PwMS	Left	1.80	0.15	4.30	0.15	6.19	0.14	2.29	0.08	2.19	0.14	4.51	0.11	?	?	?	?
						Controls		1.79	0.11	3.96	0.13	5.87	0.15	2.15	0.14	1.96	0.08	4.09	0.14	?	?	?	?
Srinivasan et al. (2021)[21]	Rarefaction – Insert earphones	21.10	80 dB nHL	1500	100-3000	PwMS	Average Across Ears	1.44	0.18	3.61	0.40	5.63	0.73	2.17	0.38	2.03	0.50	4.19	0.70	?	?	?	?
						Controls		1.43	0.16	3.48	0.19	5.30	0.24	2.04	0.17	1.82	0.18	3.86	0.21	?	?	?	?
		80.10				PwMS	Average Across Ears	?	?	?	?	6.12	0.72	?	?	?	?	?	?	?	?	?	?
						Controls		?	?	?	?	5.76	0.27	?	?	?	?	?	?	?	?	?	?

Abbreviations:
PR = Presentation Rate, PL = Presentation Level, Ave. = Number of Averages, BP = Band Pass, , $\bar{x}$ = Mean, SD = Standard Deviation, dB SL = Decibels Sensation Level, dB nHL = Decibels normal Hearing Level, PwMS = Persons with Multiple Sclerosis, IA Diff. = Inter-aural Difference, ? = not reported.
* dB SL reference to hearing thresholds (did not specify hearing thresholds to click or to pure tones)

Section IV: Multiple Sclerosis and Vestibular Function

Table S7. Characteristics of studies examining the effects of MS on vestibular function

Test Battery	Author (Year)	Study Type	Sample Size		Mean Age PwMS (years)	PwMS Age range (Years)	% Female	MS Type (sample size)	MS Duration Mean (range) years	Results of PwMS compared to controls / normative data
			PwMS	Controls						
cVEMP	Sartucci & Logi (2002)[2]	Case-Control	15	15	44.5	26-59	66.6	?	?	Significantly different
	Versino et al. (2002)[23]	Cohort	70	18	35.5	19-61	?	?	?	31.4% Abnormal
	Alpini et al. (2004)[3]	Case-control	40	25	38	17-71	57.5	?	?	70% Abnormal
	Aidar and Suzuki (2005)[4]	Case-control	15	15	39.3	?	?	?	?	Significantly different
	Patkó et al. (2007)[5]	Case-control	30	30	43.4	27-60	66.6	?	?	Significantly different
	Eleftheriadou et al. (2009)[25]	Cohort	46	40	40	20-66	45.6	RRMS	4.6 (?)	Significantly different
	Gabelić et al. (2013)[12]	Case control	30	15	?	?	46.6	RRMS	3.93 (0.2-21)	Significantly different
	Harirchian et al. (2013)[13]	Case-control	20	20	30	20-40	50	RRMS<br>SPMS	?	70% Abnormal
	Parsa et al. (2015)[29]	Cross-sectional	34	15	29.8	?	100	?	?	Significantly different
	Kavasoğlu et al. (2018)[17]	Case-control	30	31	30	18-45	60.0	?	< 1 (?)	23.3% Abnormal<br>No significant difference
	Koura and Hussein (2018)[18]	Case-control	20	10	36.80	?	65.0	?	4.4 (?)	100% Abnormal
	Delphi et al. (2021)[33]	Cross-sectional	25	25	31.43	18-45	72.0	?	?	Significantly different
	Elmoazen et al. (2021)[22]	Case-control	20	10	Brainstem lesions: 40<br>No Brainstem lesion: 34.1	?	?	?	?	Significantly different
	Cochrane et al. (2021)[34,35]	Cross-sectional	40	20	42.4	21-55	88.0	RRMS	9.9	No significant difference
oVEMP	Gabelić et al. (2013)[12]	Case control	30	15	?	?	46.6	RRMS	3.93 (0.2-21)	Significantly different
	Parsa et al. (2015)[29]	Cross-sectional	34	15	29.8	?	100	?	?	Significantly different
	Elmoazen et al. (2021)[22]	Case-control	20	10	Brainstem lesions: 40<br>No Brainstem lesion: 34.1	?	?	?	?	Significantly different
	Cochrane et al. (2021)[34,35]	Cross-sectional	40	20	42.4	21-55	88.0	RRMS	9.9	No significant difference
ENG	Zeigelboim et al. (2008)[6]	Case-control	30	0	42.23	27-64	80	RRMS	?	86.7% Abnormal
	Degirmenci et al. (2010)[26]	Cohort	30	30	37.9	23-56	56.7	RRMS	?	90% Abnormal

For peer review only

Table S7 continued: Characteristics of studies examining the effects of MS on vestibular function

Test Battery	*Author (Year)*	*Study Type*	*Sample Size*		*Mean Age PwMS (years)*	*PwMS Age range (Years)*	*% Female*	*MS Type (sample size)*	*MS Duration Mean (range) years*	*Results of PwMS compared to controls / normative data*
			PwMS	*Controls*						
**Dynamic Posturography**	Doty et al. (2018)[16]	Case-control	58	72	Males:44.61<br>Females: 44.60	?	68.9	?	Male: 7.03 (?)<br>Female: 6.54 (?)	Significantly different
**Static Posturography**	Inojosa et al. (2020)[31,32]	Cross-sectional	99	30	35.01	18-50	68.7	?	5.5 (?)	Significantly different
	Yang and Liu (2020)[30]	Cross-sectional	30	25	50.8	?	76.7	?	14 (?)	Significantly different
**Rotary Chair**	Cochrane et al. (2021)[34,35]	Cross-sectional	40	20	42.4	21-55	88.0	RRMS	9.9	Significantly different

Abbreviations: cVEMP = Cervical Vestibular Evoked Myogenic Potentials, oVEMP = Ocular Vestibular Evoked Myogenic Potentials, MS= Multiple Sclerosis, PwMS = Persons with Multiple Sclerosis, ? = not reported.
Cells shaded in grey represent test results that were abnormal or showed a difference between PwMS and Controls.

For peer review only

Extracted Vestibular Evoked Myogenic Potentials Results

Table S8: Ocular Vestibular Evoked Myogenic Potentials Parameters and Results

Author (Year)	Stimulus (polarity) - Trasducer	PR (Hz)	PL	Ave.	BP Filter (Hz)	Group	Side	Peak Latency (ms)				Amplitude (μV)	
								n1		p1		n1-p1	
								$\bar{x}$	SD	$\bar{x}$	SD	$\bar{x}$	SD
Gabelić et al. (2013)[12]	Click (?) - Headphone	1	130 dB SPL	100	5-1000	PwMS	Average Across sides	10.30	?	14.90	?	13.10	?
						Controls		9.5	?	14	?	13.3	?
Parsa et al. (2015)[29]	500 Hz Tone Burst (?) - Insert earphones	?	95 db nHL	?	?	PwMS with infratentorial plaque	Right	15.67	1.32	20.59	1.65	6.61	0.88
						PwMS without infratentorial plaque		12.21	1.00	16.90	1.32	6.61	0.60
						Controls		10.2	0.48	15.1	0.87	12.77	4.12
						PwMS with infratentorial plaque	Left	15.74	1.66	21.40	2.88	6.48	0.68
						PwMS without infratentorial plaque		11.78	0.89	16.92	0.82	6.82	0.75
						Controls		10.3	0.51	14.9	0.84	12.6	4.04
Elmoazen et al. (2021)[22]	500 Hz Tone Burst (condensation) -Supra aural headphones	5	95 dB nHL	150	1-1000	PwMS with Brainstem lesions	Average Across sides	11.43	1.42	16.86	1.79	3.59	3.82
						PwMS without Brainstem lesions		10.58	2.50	15.81	1.42	3.22	2.00
						Controls		10.09	0.92	14.71	1.20	2.76	1.28
Cochrane et al. (2021)[34]	Unspecified Tone Burst (?) – earphone unspecified	?	?	100	?	PwMS	?	8.50	1.70	12.90	2.20	5.20	3.50
						Controls		8.40	1.50	12.30	2.10	4.70	2.50

Abbreviations:
PR = Presentation Rate, PL = Presentation Level, Ave. = Number of Averages, BP = Band Pass, , $\bar{x}$ = Mean, SD = Standard Deviation, dB SPL = Decibels Sound Pressure Level, dB nHL = Decibels normal Hearing Level, PwMS = Persons with Multiple Sclerosis, ? = not reported.

For peer review only

Table S9: Cervical Vestibular Evoked Myogenic Potentials Parameters and Results

Author (Year)	Stimulus (polarity) - Trasducer	PR (Hz)	PL	Ave.	BP Filter (Hz)	Group	Side	Peak Latency (ms)				Amplitude (µV)	
								p13		n23		p13-n23	
								x̄	SD	x̄	SD	x̄	SD
Sartucci and Logi (2002)[2]	Click (Rarefaction) - Headphone	3	140 dB SPL	256	10-1500	PwMS	Right	15.30	3.10	22.50	2.30	63.00	65.50
						Controls	Right	12.5	0.8	21.3	1.5	186.4	82.8
						PwMS	Left	14.00	2.30	20.80	2.30	73.10	58.80
						Controls	Left	12.3	0.9	20.8	1.3	141.1	85.8
Alpini et al. (2004)[3]	Click (Rarefaction) - Headphone	5	95 dB nHL + 70 dB SPL contralateral Masking	400	?	PwMS	Right	14.40	?	22.40	?	?	?
							Left	14.90	?	22.50	?	?	?
Aidar and Suzuki (2005)[4]	Click (Rarefaction) - Headphone	2	95 dB HL	200	20-1000	PwMS	Right	13.40	2.87	18.64	2 .67	12 .17	6 .50
						Controls	Right	11 .12	1.64	15.15	2.51	7.54	5.95
						PwMS	Left	13.49	1 .77	17.84	1.27	13.30	3 .63
						Controls	Left	10.76	1.59	14.86	2.85	7.23	4.76
Patkó et al. (2007)[5]	Click (Rarefaction) - Headphone	5	133 dB SPL	250	10-1600	PwMS	Right	14.40	1.50	21.30	3.20	11.61	3.63
						Controls	Right	12.7	1.5	21.3	3.7	16.02	5.34
						PwMS	Left	14.8	1.5	21.2	3.2	11.07	2.87
						Controls	Left	12.9	1.9	20.7	2.8	13.85	4.3
Eleftheriadou et al. (2009)[25]	Click (Rarefaction) - Headphone	5	105 dB HL	250	5-1500	PwMS with brainstem leasions	Average acrross sides	15.48	1.40	23.94	1.51	?	?
						PwMS without brainstem leasions	Average acrross sides	14.56	1.20	23.25	1.42	?	?
						Controls	Average acrross sides	12.67	1.1	21.66	1.11	?	?
Gabelić et al. (2013)[12]	Click (?) - Headphone	1	130 dB SPL	100	5-1000	PwMS	Average across sides	15.80	?	24.90	?	1.90	?
						Controls	Average across sides	14.6	?	22.1	?	1.6	?
Harirchian et al. (2013)[13]	Click (Rarefaction) - Headphone	2	95 db nHL + 40 dB nHL contralateral Masking	200	10-1000	PwMS	Right	22.20	9.30	30.02	11.20	?	?
						Controls	Right	12.9	1.4	21.3	1.6	?	?
						PwMS	Left	20.20	7.05	30.71	16.20	?	?
						Controls	Left	13.1	1.9	20.6	1.9	?	?
						PwMS	IA Diff.	2.05	8.80	0.70	10.90	?	?
						Controls	IA Diff.	0.17	2.2	0.4	2.5	?	?

For peer review only - http://bmjopen.bmj.com/site/about/guidelines.xhtml

Table S9 Continued: Cervical Vestibular Evoked Myogenic Potentials Parameters and Results

Author (Year)	Stimulus (polarity) - Trasducer	PR (Hz)	PL	Ave.	BP Filter (Hz)	Group	Side	Peak Latency (ms)				Amplitude (µV)	
								p13		n23		p13-n23	
								x̄	SD	x̄	SD	x̄	SD
Parsa et al. (2015)[29]	500 Hz Tone Burst (?) -Insert earphones	?	95 db nHL	?	?	PwMS with infratentorial plaque	Right	18.75	1.81	28.15	1.43	118.82	29.97
						PwMS without infratentorial plaque		17.44	1.03	26.89	1.87	135.00	20.31
						Controls		15.25	0.9	24.55	1.07	180	44.97
						PwMS with infratentorial plaque	Left	19.11	1.72	28.88	2.27	119.65	26.02
						PwMS without infratentorial plaque		17.21	1.05	26.87	1.36	133.78	22.89
						Controls		15.31	0.93	24.48	1.18	175	57.59
Kavasoğlu et al. (2017)[17]	Click (Rarefaction) - Headphone	5	100 dB nHL	100	10-1000	PwMS	Right	12.90	2.10	21.20	1.78	7.10	2.80
						Controls		12	0.9	20.6	1.95	7.7	3.1
						PwMS	Left	12.90	2.20	21.40	2.10	7.00	2.50
						Controls		12.03	0.96	20.76	1.63	7.6	3
Koura and Hussein (2018)[18]	500 Hz Tone Burst (Rarefaction) -TDH 39 Headphones	5.1	95 db HL	200	?	PwMS	Right	17.14	0.71	26.82	0.84	41.88	1. 71
						Controls		13.83	1.145	20.76	3.43	61.18	34.64
						PwMS	Left	17.20	0.56	27.17	0.75	54.95	28.27
						Controls		14.43	0.702	22.3	1.725	65.74	18.51
Delphi et al. (2021)[33]	500 Hz Tone Burst (?) -?	5.1	95 dB nHL	?	10-1200	PwMS	Right	18.20	1.93	27.37	2.45	121.34	29.05
						Controls		14.96	1.02	24.21	2.10	169.21	48.76
						PwMS	Left	18.95	2.01	27.21	2.80	-	-
						Controls		15.02	1.10	24.12	2.30	-	-
Elmoazen et al. (2021)[22]	500 Hz Tone Burst (condensation) -Supra aural headphones	5	95 dB nHL	150	1-1000	PwMS with Brainstem lesions	Average across sides	16.49	1.49	25.96	1.93	66.25	52.42
						PwMS without Brainstem lesions		15.21	1.57	23.97	2.06	93.12	78.01
						Controls		14.05	0.94	23.36	2.14	144.42	122.73
Cochrane et al. (2021)[34]	Unspecified Tone Burst (?) – earphone unspecified	?	?	?	?	PwMS	?	13.70	1.30	21.10	2.60	12.70	7.60
						Controls		13.30	0.50	20.60	1.80	16.50	7.10

Abbreviations:
PR = Presentation Rate, PL = Presentation Level, Ave. = Number of Averages, BP = Band Pass, , x̄ = Mean, SD = Standard Deviation, dB SPL = Decibels Sound Pressure Level, dB HL = Decibels Heraing Level, dB nHL = Decibels normal Hearing Level, PwMS = Persons with Multiple Sclerosis, IA Diff. = Inter-aural Difference, ? = not reported. Cells Highlighted in Yellow indicate that "normalized amplitudes" were reported as opposed to absolute amplitudes reported by other studies. Cells Highlighted in Green indicate results were reported without specifying side or if they are averaged across sides.

1 Japaridze G, Shakarishvili R, Kevanishvili Z. Auditory brainstem, middle-latency, and slow cortical responses in multiple sclerosis. *Acta Neurol Scand* 2002;**106**:47–53. doi:10.1034/j.1600-0404.2002.01226.x

2 Sartucci F, Logi F. Vestibular-evoked myogenic potentials: A method to assess vestibulo-spinal conduction in multiple sclerosis patients. *Brain Res Bull* 2002;**59**:59–63. doi:10.1016/s0361-9230(02)00842-0

3 Alpini D, Pugnetti L, Caputo D, *et al.* Vestibular evoked myogenic potentials in multiple sclerosis: clinical and imaging correlations. *Mult Scler* 2004;**10**:316–21. doi:10.1191/1352458504ms1041oa

4 Aidar RC, Suzuki FA. Vestibular evoked myogenic potential: new perspectives in multiple sclerosis. *Braz J Otorhinolar* 2005;**71**:48–54. doi:10.1016/s1808-8694(15)31284-2

5 Patkó T, Simó M, Arányi Z. Vestibular click-evoked myogenic potentials: sensitivity and factors determining abnormality in patients with multiple sclerosis. *Mult Scler J* 2007;**13**:193–8. doi:10.1177/1352458506070940

6 Zeigelboim BS, Arruda WO, Mangabeira-Albernaz PL, *et al.* Vestibular findings in relapsing, remitting multiple sclerosis: a study of thirty patients. *Int Tinnitus J* 2008;**14**:139–45.

7 Lima TMA, Crato AN, Mancini PC, *et al.* Alterations in early auditory evoked potentials in multiple sclerosis patients. *Braz J Otorhinolar* 2009;**75**:177–81. doi:10.1016/s1808-8694(15)30775-8

8 Lewis MS, Lilly DJ, Hutter MM, *et al.* Audiometric hearing status of individuals with and without multiple sclerosis. *J Rehabilitation Res Dev* 2010;**47**:669–78. doi:10.1682/jrrd.2010.01.0010

9 Matas CG, Matas SL de A, Oliveira CRS de, *et al.* Auditory evoked potentials and multiple sclerosis. *Arq Neuro-psiquiat* 2010;**68**:528–34. doi:10.1590/s0004-282x2010000400010

10 Doty RL, Tourbier I, Davis S, *et al.* Pure-Tone Auditory Thresholds Are Not Chronically Elevated in Multiple Sclerosis. *Behav Neurosci* 2012;**126**:314–24. doi:10.1037/a0027046

11 Saberi A, Hatamian HR, Nemati S, *et al.* Hearing statement in multiple sclerosis: a case control study using auditory brainstem responses and otoacoustic emissions. *Acta Medica Iranica* 2012;**50**:679–83.

12 Gabeli T, Krbot M, Šefer AB, *et al.* Ocular and Cervical Vestibular Evoked Myogenic Potentials in Patients With Multiple Sclerosis. *J Clin Neurophysiol* 2013;**30**:86–91. doi:10.1097/wnp.0b013e31827eda0c

13 Harirchian MH, Karimi N, Nafisi S, *et al.* Vestibular evoked myogenic potential for diagnoses of multiple sclerosis: is it beneficial? *Medicinski Glasnik Official Publ Medical Assoc Zenica-doboj Canton Bosnia Herzegovina* 2013;**10**:321–6.

For peer review only - http://bmjopen.bmj.com/site/about/guidelines.xhtml

14 Valadbeigi A, Weisi F, Rohbakhsh N, *et al.* Central auditory processing and word discrimination in patients with multiple sclerosis. *Eur Arch Oto-rhino-l* 2014;**271**:2891–6. doi:10.1007/s00405-013-2776-6

15 Pokryszko-Dragan A, Bilinska M, Gruszka E, *et al.* Assessment of visual and auditory evoked potentials in multiple sclerosis patients with and without fatigue. *Neurol Sci* 2015;**36**:235–42. doi:10.1007/s10072-014-1953-8

16 Doty RL, MacGillivray MR, Talab H, *et al.* Balance in multiple sclerosis: relationship to central brain regions. *Exp Brain Res* 2018;**236**:2739–50. doi:10.1007/s00221-018-5332-1

17 Kavasoğlu G, Gökçay F, Yüceyar N, *et al.* Cervical vestibular-evoked myogenic potentials in patients with multiple sclerosis: sensitive in detecting brainstem involvement? *Neurol Sci* 2018;**39**:365–71. doi:10.1007/s10072-017-3215-z

18 Koura R, Hussein M. Vestibular-evoked myogenic potential: an easy neurophysiological tool for evaluating brain stem involvement in multiple sclerosis. *Egypt J Otolaryngology* 2018;**34**:144–8. doi:10.4103/ejo.ejo_73_17

19 Elbeltagy R, Gad N, Ismail M. Central auditory function in multiple sclerosis patients. *Indian J Otology* 2019;**25**:90–6. doi:10.4103/indianjotol.indianjotol_80_18

20 Rishiq D, Harkrider A, Springer C, *et al.* Click-evoked and speech-evoked auditory brainstem responses from individuals with multiple sclerosis. *Neurosci Lett* 2021;**740**:135460. doi:10.1016/j.neulet.2020.135460

21 Srinivasan VS, Krishna R, Munirathinam BR. Effectiveness of Brainstem Auditory Evoked Potentials Scoring in Evaluating Brainstem Dysfunction and Disability Among Individuals With Multiple Sclerosis. *Am J Audiol* 2021;**30**:255–65. doi:10.1044/2020_aja-20-00155

22 Elmoazen D, Kozou H, Gawies M, *et al.* Assessment of Cervical and Ocular Vestibular Evoked Myogenic Potentials in Multiple Sclerosis PatientsS. *Egyptian Journal of Ear, Nose, Throat and Allied Sciences* 2021;**22**:1–9. doi:10.21608/ejentas.2020.44108.1262

23 Versino M, Colnaghi S, Callieco R, *et al.* Vestibular evoked myogenic potentials in multiple sclerosis patients. *Clin Neurophysiol* 2002;**113**:1464–9. doi:10.1016/s1388-2457(02)00155-4

24 Burina A, Sinanović O, Smajlović D, *et al.* Some Aspects of Balance Disorder in Patients with Multiple Sclerosis. *Bosnian J Basic Med* 2008;**8**:80–5. doi:10.17305/bjbms.2008.3003

25 Eleftheriadou A, Deftereos SN, Zarikas V, *et al.* The diagnostic value of earlier and later components of Vestibular Evoked Myogenic Potentials (VEMP) in Multiple Sclerosis. *J Vestib Res* 2009;**19**:59–66. doi:10.3233/ves-2009-0342

26 Degirmenci E, Bir LS, Ardic FN. Clinical and electronystagmographical evaluation of vestibular symptoms in relapsing remitting multiple sclerosis. *Neurol Res* 2010;**32**:986–91. doi:10.1179/016164110x12681290831405

For peer review only - http://bmjopen.bmj.com/site/about/guidelines.xhtml

27 Kaytancı E, Ozdamar OI, Acar GO, *et al.* Evaluation of transiently evoked otoacoustic emissions and auditory brainstem responses in patients with multiple sclerosis. *Ear Nose Throat J* 2016;**95**:E12–7.

28 Di Mauro RD, Di Girolamo SD, Ralli M, *et al.* Subclinical cochlear dysfunction in newly diagnosed relapsing-remitting multiple sclerosis. *Mult Scler Relat Dis* 2019;**33**:55–60. doi:10.1016/j.msard.2019.05.020

29 Parsa MS, Mohammadkhani G, Hajabolhassani F, *et al.* Cervical and ocular vestibular evoked myogenic potentials in multiple sclerosis participants. *Medical J Islamic Repub Iran* 2015;**29**:164.

30 Yang F, Liu X. Relative importance of vision and proprioception in maintaining standing balance in people with multiple sclerosis. *Mult Scler Relat Dis* 2020;**39**:101901. doi:10.1016/j.msard.2019.101901

31 Inojosa H, Schriefer D, Klöditz A, *et al.* Balance Testing in Multiple Sclerosis—Improving Neurological Assessment With Static Posturography? *Front Neurol* 2020;**11**:135. doi:10.3389/fneur.2020.00135

32 Inojosa H, Schriefer D, Trentzsch K, *et al.* Visual Feedback and Postural Control in Multiple Sclerosis. *J Clin Medicine* 2020;**9**:1291. doi:10.3390/jcm9051291

33 Delphi M, Sayaf M, Taheri F, *et al.* Audio and vestibular analyses in patient with multiple sclerosis: a case–control study. *Hear Balance Commun* 2021;**19**:1–6. doi:10.1080/21695717.2021.1933318

34 Cochrane GD, Christy JB, Motl RW. Comprehensive Clinical Assessment of Vestibular Function in Multiple Sclerosis. *J Neurol Phys Ther* 2021;**45**:228–34. doi:10.1097/npt.0000000000000358

35 Cochrane GD, Christy JB, Sandroff BM, *et al.* Cognitive and Central Vestibular Functions Correlate in People With Multiple Sclerosis. *Neurorehab Neural Re* 2021;**35**:1030–8. doi:10.1177/15459683211046268

For peer review only

NIHR | National Institute for Health Research

PROSPERO
International prospective register of systematic reviews

Citation

Evrim Gur, Karolina Kluk, Ghada BinKhamis. Multiple Sclerosis and audio-vestibular function: systematic review. PROSPERO 2020 CRD42020180094 Available from: https://www.crd.york.ac.uk/prospero/display_record.php?ID=CRD42020180094

Review question

Primary Research Question:

How does Multiple Sclerosis affect the audio-vestibular system?

Secondary research questions:

- Does Multiple Sclerosis affect auditory function?

- Does Multiple Sclerosis affect vestibular function?

Searches

We will search the following electronic bibliographic databases: PubMed, NICE, Scopus, Web of Science using the following search terms: ''Multiple Sclerosis and hearing'', ''Multiple Sclerosis and dizziness'', ''Multiple Sclerosis and vertigo'', ''Multiple Sclerosis and vestibular dysfunction'', ''Multiple Sclerosis and audio-vestibular effect''.

Research papers published between 2000 and 2020 will be reviewed and language is restricted with English.

- Searches will be re-run prior to the final analysis to identify any further studies.

- Unpublished studies will not be sought.

Types of study to be included

The following study types will be included:

Randomised Controlled Trial, Case-control, Cohort Study, Cross-Sectional Study, Observational study, Retrospective.

Condition or domain being studied

Multiple Sclerosis, Hearing and Vestibular system

Participants/population

Inclusion: Participants over the age of 18 years diagnosed with one of the types of Multiple Sclerosis including Relapsing- Remitting Multiple Sclerosis, Secondary-Progressive Multiple Sclerosis, Primary-Progressive Multiple Sclerosis, Progressive-Relapsing Multiple Sclerosis

Exclusion: Children under the age of 18 with Multiple Sclerosis

Intervention(s), exposure(s)

For evaluating auditory function:

Behavioural measurement: Pure tone audiometry,

Electrophysiological measurements: Otoacoustic Emissions, Auditory Brainstem Evoked Potentials, Auditory Cortical Evoked Potentials

Imaging: Functional Magnetic Resonance Imaging

For peer review only

For evaluating vestibular function:

Vestibular Evoked Myogenic Potentials, Videonystagmography, Electonystagmography, Posturography, Rotary Chair

Comparator(s)/control

Normal controls over the age of 18 years who do not have Multiple Sclerosis

Context

The geographical location of study participants will not be limited. All studies with available English full texts that investigated the association between Multiple Sclerosis and audio-vestibular function will be included. The age of participants will be limited to 18 years and above.

Main outcome(s)

- Abnormality in Pure tone Audiometry

- Abnormality in absolute peak latencies and waveform morphology of auditory brainstem evoked potentials and auditory cortical evoked potentials

- Abnormal findings in functional MRI that is assessing auditory function as compared to controls

- Abnormality in cervical and ocular vestibular Evoked Myogenic Potentials' latency, morphology, inter-aural differences

- Evaluation of presence and characteristics (type, duration, weak side etc.) of nystagmus in Videonystagmography and Electonystagmography and Rotary Chair.

- High function or Low function on Posturography, vestibular or somatosensory or visual hypofunction or hyperfunction

Measures of effect

-differences between patients with Multiple Sclerosis and healthy controls on Pure Tone audiometry thresholds

-differences between patients with Multiple Sclerosis and healthy controls in absolute peak latencies and waveform morphology of auditory brainstem evoked potentials and auditory cortical evoked potential

-differences in cervical and ocular vestibular Evoked Myogenic Potentials' latency, morphology between patients with Multiple Sclerosis and healthy controls and inter-aural differences

-differences in characteristics of nystagmus (type, duration, weak side etc.) between patients with Multiple Sclerosis and healthy controls on Videonystagmography, Electronystagmography and Rotary Chair

-differences between patients with Multiple Sclerosis and healthy controls on Posturography results (Sensory Organization Test, Motor Control Test)

-determined lesion on regions that might affect the audio-vestibular function of the central nervous system with Functional MRI on patients with multiple sclerosis as compared to healthy controls

Additional outcome(s)

For speech perception and temporal processing:

- Abnormal responses on Gaps-in-Noise test

- Abnormal Word Discrimination Scores

- Abnormal responses on Duration pattern sequence test

For peer review only - http://bmjopen.bmj.com/site/about/guidelines.xhtml

Measures of effect

-differences between patients with Multiple Sclerosis and healthy controls on Word Discrimination scores

-differences between patients with Multiple Sclerosis and healthy controls on responses of Gaps-in-Noise test

-differences between patients with Multiple Sclerosis and healthy controls on responses of Duration pattern sequence test

Data extraction (selection and coding)

- Selection

The Joanna Briggs Institute Critical Appraisal tools will be used for study selection.

Newcastle -Ottowa Quality Assessment Scale will be used for the quality assessment.

Study selection: one author will select and this selection will be reviewed by two authors.

The final decision will be recorded in an excel spreadsheet.

Any disagreements will be resolved by discussion between the three authors.

- Data Extraction

The Joanna Briggs Institute Critical Appraisal tools will be used for data extraction. Excel spreadsheet will be used for listing extracted data.

The following data will be extracted from included studies if available:

Details of the study: Aim of study; Study design.

Population: Methods of recruitment; Diagnosis and inclusion/exclusion criteria for patients; Syndrome differentiation (if available); Course of conditions/symptoms; Other health problems; Number of patients: screened, eligible, enrolled, included in analysis and each measure; Age; Gender.

Details of the pathology: specific lesion site, primary or secondary, stable or progressive, additional handicaps related to Multiple Sclerosis,

Details of the methodology: Test techniques eg. Pure Tone Audiometry, Vestibular Evoked Myogenic Potentials, stimulus and recording parameters, electrode impendence and inter-electrode impedance levels.

One author will extract and a second author will check the correctness of the extracted data.

Any disagreements will be resolved by discussion between the three authors.

Investigators will not be contacted for unreported missing data (missing data will be excluded) or additional information due to time constraints and given current COVID-19 pandemic related global circumstances.

Risk of bias (quality) assessment

All assessment of the risk of bias will be done by two reviewers independently. The randomised controlled trials will be assessed using Cochrane Collaboration’s tool for assessing risk of bias in randomised trials to identify selection bias, reporting bias, performance bias, detection bias, and other sources of bias. The case-control studies will be assessed using the Risk of Bias Assessment Tool for Nonrandomized Studies (RoBANS) to examine selection of participants, confounding variables, intervention measurement, blinding of outcome assessment, intervention measurement, incomplete outcome data and selective outcome reporting. The cohort studies will be assessed according to the Newcastle Ottawa Scale (NOS). A judgement of ‘low

For peer review only

risk' of bias, 'high risk' or bias, or 'unclear risk' of bias will be provided for each domain. Any disagreements will be resolved by discussion or by involving a third author until consensus is reached.

Strategy for data synthesis

- Data will be synthesised from articles that use the same test methods (Auditory Brainstem Evoked Potentials or Vestibular Evoked Myogenic Potentials or Pure Tone Audiometry) there should be at least 5 participants in each study and at least 2 articles should exist for each test method.

- Meta-analyses will be done where possible.

- Only Vestibular Evoked Myogenic Potential thresholds, Auditory Brainstem Evoked Potential (amplitudes and latencies, Normal/abnormal results) and Pure Tone Audiometry thresholds will be synthesised. Mean and Standard Deviation values will be synthesised and effect size will be calculated using RevMan 5.3 software (Cochran Collaboration).

- Cochran Q test will be used for heterogeneity and inconsistency between studies will be defined with the I^2 test. A value of $p< 0.10$ will be evaluated as statistically significant. If there is a possible heterogeneity, articles will be removed one by one to detect the problematic one.

- If there is a possibility of publication bias, the contour-enhanced funnel plot of each outcome using each trial's effect size against the sample size will be evaluated. The Egger test will be used for small study effect and a value of $p<0.10$ will be considered as a statistically significant.

Analysis of subgroups or subsets

- As different types of Multiple Sclerosis have different clinical presentations, they might have different impact on audio-vestibular function.

- Differences in audio-vestibular functions between different types of Multiple Sclerosis: Relapsing-Remitting Multiple Sclerosis, Secondary-Progressive Multiple Sclerosis, Primary-Progressive Multiple Sclerosis, and Progressive-Relapsing Multiple Sclerosis will be investigated.

- As stated above, only Vestibular Evoked Myogenic Potential thresholds, Auditory Brainstem Evoked Potential (amplitudes and latencies) and Pure Tone Audiometry thresholds will be synthesised for each type of Multiple Sclerosis.

Contact details for further information

Karolina Kluk

Evrim Gür

Ghada BinKhamis

Evrim.gur@postgrad.manchester.ac.uk

Organisational affiliation of the review

The University of Manchester

Review team members and their organisational affiliations

Ms Evrim Gur. The University of Manchester
Dr Karolina Kluk. The University of Manchester
Dr Ghada BinKhamis. The University of Manchester, UK and Rehabilitation Hospital King Fahad Medical City

Type and method of review

Diagnostic, Systematic review

For peer review only

NIHR | National Institute for Health Research

PROSPERO
International prospective register of systematic reviews

Anticipated or actual start date
13 July 2020

Anticipated completion date
30 September 2020

Funding sources/sponsors
none

Conflicts of interest

Language
English

Country
England

Stage of review
Review Ongoing

Subject index terms status
Subject indexing assigned by CRD

Subject index terms
Communications Media; Gray Matter; Humans; Multiple Sclerosis

Date of registration in PROSPERO
04 September 2020

Date of first submission
13 July 2020

Stage of review at time of this submission

Stage	Started	Completed
Preliminary searches	No	No
Piloting of the study selection process	Yes	No
Formal screening of search results against eligibility criteria	No	No
Data extraction	No	No
Risk of bias (quality) assessment	No	No
Data analysis	No	No

The record owner confirms that the information they have supplied for this submission is accurate and complete and they understand that deliberate provision of inaccurate information or omission of data may be construed as scientific misconduct.
The record owner confirms that they will update the status of the review when it is completed and will add publication details in due course.

Versions
04 September 2020

For peer review only

NIHR | National Institute for Health Research

PROSPERO
International prospective register of systematic reviews

For peer review only

PRISMA 2020 Checklist

Section and Topic	Item #	Checklist item	Location where item is reported
**TITLE**			
Title	1	Identify the report as a systematic review.	Page 1 & 4
**ABSTRACT**			
Abstract	2	See the PRISMA 2020 for Abstracts checklist.	Page 3 – 4
**INTRODUCTION**			
Rationale	3	Describe the rationale for the review in the context of existing knowledge.	Page 4 – 6
Objectives	4	Provide an explicit statement of the objective(s) or question(s) the review addresses.	Page 6
**METHODS**			
Eligibility criteria	5	Specify the inclusion and exclusion criteria for the review and how studies were grouped for the syntheses.	Page 7 – 9
Information sources	6	Specify all databases, registers, websites, organisations, reference lists and other sources searched or consulted to identify studies. Specify the date when each source was last searched or consulted.	Page 6
Search strategy	7	Present the full search strategies for all databases, registers and websites, including any filters and limits used.	Supplement
Selection process	8	Specify the methods used to decide whether a study met the inclusion criteria of the review, including how many reviewers screened each record and each report retrieved, whether they worked independently, and if applicable, details of automation tools used in the process.	Page 7 – 8
Data collection process	9	Specify the methods used to collect data from reports, including how many reviewers collected data from each report, whether they worked independently, any processes for obtaining or confirming data from study investigators, and if applicable, details of automation tools used in the process.	Page 8 – 9
Data items	10a	List and define all outcomes for which data were sought. Specify whether all results that were compatible with each outcome domain in each study were sought (e.g. for all measures, time points, analyses), and if not, the methods used to decide which results to collect.	Page 7
	10b	List and define all other variables for which data were sought (e.g. participant and intervention characteristics, funding sources). Describe any assumptions made about any missing or unclear information.	Page 8 – 9
Study risk of bias assessment	11	Specify the methods used to assess risk of bias in the included studies, including details of the tool(s) used, how many reviewers assessed each study and whether they worked independently, and if applicable, details of automation tools used in the process.	Page 8
Effect measures	12	Specify for each outcome the effect measure(s) (e.g. risk ratio, mean difference) used in the synthesis or presentation of results.	Page 9
Synthesis methods	13a	Describe the processes used to decide which studies were eligible for each synthesis (e.g. tabulating the study intervention characteristics and comparing against the planned groups for each synthesis (item #5)).	Not Applicable
	13b	Describe any methods required to prepare the data for presentation or synthesis, such as handling of missing summary statistics, or data conversions.	Not Applicable
	13c	Describe any methods used to tabulate or visually display results of individual studies and syntheses.	Not Applicable
	13d	Describe any methods used to synthesize results and provide a rationale for the choice(s). If meta-analysis was performed, describe the model(s), method(s) to identify the presence and extent of statistical heterogeneity, and software package(s) used.	Not Applicable
	13e	Describe any methods used to explore possible causes of heterogeneity among study results (e.g. subgroup analysis, meta-regression).	Not Applicable
	13f	Describe any sensitivity analyses conducted to assess robustness of the synthesized results.	Not Applicable
Reporting bias assessment	14	Describe any methods used to assess risk of bias due to missing results in a synthesis (arising from reporting biases).	Not Applicable
Certainty assessment	15	Describe any methods used to assess certainty (or confidence) in the body of evidence for an outcome.	Not Applicable
**RESULTS**			

For peer review only - http://bmjopen.bmj.com/site/about/guidelines.xhtml

PRISMA 2020 Checklist

Section and Topic	Item #	Checklist item	Location where item is reported
Study selection	16a	Describe the results of the search and selection process, from the number of records identified in the search to the number of studies included in the review, ideally using a flow diagram.	**Page 9 & Figure 1**
	16b	Cite studies that might appear to meet the inclusion criteria, but which were excluded, and explain why they were excluded.	**Not applicable**
Study characteristics	17	Cite each included study and present its characteristics.	**Table 1 and Table 2 & Supplement Tables S4 and S7**
Risk of bias in studies	18	Present assessments of risk of bias for each included study.	**Supplement Tables S1 – S3**
Results of individual studies	19	For all outcomes, present, for each study: (a) summary statistics for each group (where appropriate) and (b) an effect estimate and its precision (e.g. confidence/credible interval), ideally using structured tables or plots.	**(a) Supplement Tables S5, S6, S8, S9**<br>**(b) Not applicable**
Results of syntheses	20a	For each synthesis, briefly summarise the characteristics and risk of bias among contributing studies.	**Not applicable**
	20b	Present results of all statistical syntheses conducted. If meta-analysis was done, present for each the summary estimate and its precision (e.g. confidence/credible interval) and measures of statistical heterogeneity. If comparing groups, describe the direction of the effect.	**Not applicable**
	20c	Present results of all investigations of possible causes of heterogeneity among study results.	**Not applicable**
	20d	Present results of all sensitivity analyses conducted to assess the robustness of the synthesized results.	**Not applicable**
Reporting biases	21	Present assessments of risk of bias due to missing results (arising from reporting biases) for each synthesis assessed.	**Not applicable**
Certainty of evidence	22	Present assessments of certainty (or confidence) in the body of evidence for each outcome assessed.	**Not applicable**
**DISCUSSION**			
Discussion	23a	Provide a general interpretation of the results in the context of other evidence.	**Page 18 – 24**
	23b	Discuss any limitations of the evidence included in the review.	**Page 18 – 24**
	23c	Discuss any limitations of the review processes used.	**Not applicable**
	23d	Discuss implications of the results for practice, policy, and future research.	**Page 18 – 24**
**OTHER INFORMATION**			
Registration and protocol	24a	Provide registration information for the review, including register name and registration number, or state that the review was not registered.	**Abstract & Methods**
	24b	Indicate where the review protocol can be accessed, or state that a protocol was not prepared.	**Abstract & Methods**
	24c	Describe and explain any amendments to information provided at registration or in the protocol.	**Not applicable**
Support	25	Describe sources of financial or non-financial support for the review, and the role of the funders or sponsors in the review.	**Not applicable**
Competing interests	26	Declare any competing interests of review authors.	**Page 26**
Availability of data, code and other materials	27	Report which of the following are publicly available and where they can be found: template data collection forms; data extracted from included studies; data used for all analyses; analytic code; any other materials used in the review.	**Not applicable**

From: Page MJ, McKenzie JE, Bossuyt PM, Boutron I, Hoffmann TC, Mulrow CD, et al. The PRISMA 2020 statement: an updated guideline for reporting systematic reviews. BMJ 2021;372:n71. doi: 10.1136/bmj.n71

For more information, visit: http://www.prisma-statement.org/

For peer review only

BMJ Open

The effects of Multiple Sclerosis on the audio-vestibular system: A systematic review

Journal:	*BMJ Open*
Manuscript ID	bmjopen-2021-060540.R2
Article Type:	Original research
Date Submitted by the Author:	07-Jun-2022
Complete List of Authors:	Gür, Evrim; The University of Manchester, Manchester Centre for Audiology and Deafness (ManCAD), School of Health Sciences, Ellen Wilkinson Building Manchester, UK M13 9PL<br>Binkhamis, Ghada; King Fahad Medical City, Communication and Swallowing Disorders; The University of Manchester, Manchester Centre for Audiology and Deafness (ManCAD)<br>Kluk, Karolina; The University of Manchester, Manchester Centre for Audiology and Deafness (ManCAD), School of Health Sciences, Ellen Wilkinson Building Manchester, UK M13 9PL
**Primary Subject Heading**:	Ear, nose and throat/otolaryngology
Secondary Subject Heading:	Neurology
Keywords:	Audiology < OTOLARYNGOLOGY, Adult otolaryngology < OTOLARYNGOLOGY, Multiple sclerosis < NEUROLOGY

SCHOLARONE™
Manuscripts

I, the Submitting Author has the right to grant and does grant on behalf of all authors of the Work (as defined in the below author licence), an exclusive licence and/or a non-exclusive licence for contributions from authors who are: i) UK Crown employees; ii) where BMJ has agreed a CC-BY licence shall apply, and/or iii) in accordance with the terms applicable for US Federal Government officers or employees acting as part of their official duties; on a worldwide, perpetual, irrevocable, royalty-free basis to BMJ Publishing Group Ltd ("BMJ") its licensees and where the relevant Journal is co-owned by BMJ to the co-owners of the Journal, to publish the Work in this journal and any other BMJ products and to exploit all rights, as set out in our licence.

The Submitting Author accepts and understands that any supply made under these terms is made by BMJ to the Submitting Author unless you are acting as an employee on behalf of your employer or a postgraduate student of an affiliated institution which is paying any applicable article publishing charge ("APC") for Open Access articles. Where the Submitting Author wishes to make the Work available on an Open Access basis (and intends to pay the relevant APC), the terms of reuse of such Open Access shall be governed by a Creative Commons licence – details of these licences and which Creative Commons licence will apply to this Work are set out in our licence referred to above.

Other than as permitted in any relevant BMJ Author's Self Archiving Policies, I confirm this Work has not been accepted for publication elsewhere, is not being considered for publication elsewhere and does not duplicate material already published. I confirm all authors consent to publication of this Work and authorise the granting of this licence.

For peer review only

The effects of Multiple Sclerosis on the audio-vestibular system: A systematic review

Evrim Gür[1,*], Ghada BinKhamis[1,2,*] and Karolina Kluk[1]

[1]*Manchester Centre for Audiology and Deafness, Manchester Academic Health Science Centre, University of Manchester, Manchester, UK;* [2]*Department of Communication and Swallowing Disorders, King Fahad Medical City, Riyadh, Saudi Arabia*

*E.G. and G.B. equally contributed to this work

Address for correspondence:

Ghada BinKhamis: ghada.binkhamis@manchester.ac.uk / gbinkhamis@gmail.com

Karolina Kluk: Karolina.Kluk@manchester.ac.uk

Evrim Gür: evrimguur@gmail.com,

Manchester Centre for Audiology and Deafness (ManCAD), School of Health Sciences, Faculty of Biology Medicine and Health, Room B2.15, Ellen Wilkinson Building, University of Manchester, Oxford Road, Manchester, M13 9PL, United Kingdom.

ORCiD:

1. Ghada BinKhamis: 0000-0002-7696-1073
2. Karolina Kluk: 0000-0003-3638-2787

Word count: 5532

For peer review only

ABSTRACT

Objective: Systematically investigate effects of Multiple Sclerosis (MS) on the audio-vestibular system.

Methods: Systematic review of literature investigating audio-vestibular conditions in persons with MS (PwMS) aged $\geq$ 18 years. PUBMED, SCOPUS, NICE, Web of Science were searched. Randomised control trials, cohort, case-control, observational, retrospective studies in English, published from 2000 to 21st November 2021, evaluated PwMS with at least one outcome (Pure Tone Audiometry, Auditory Brainstem Response, Otoacoustic Emissions, Cortical Auditory Evoked Potentials, Functional MRI assessing auditory function, Vestibular Evoked Myogenic Potentials, Videonystagmography, Electonystagmography, Posturography, Rotary Chair, Gaps-in-Noise, Word Discrimination Scores, Duration Pattern Sequence Test) were included. Study selection and assessments of bias were independently conducted by two reviewers using the Risk of Bias Assessment Tool for Nonrandomized Studies, Newcastle Ottawa Scale (NOS), and the NOS adapted for cross-sectional studies.

Results: 35 studies were included. Auditory function was evaluated in 714 PwMS and 501 controls, vestibular function was evaluated in 682 PwMS and 446 controls. Peripheral auditory function results were contradictory between studies; some found abnormalities in PwMS, and others found no differences. Tests of brainstem and central auditory functions were more consistently found to be abnormal in PwMS. Most vestibular tests were reported as abnormal in PwMS, abnormalities were either peripheral or central or both. However, quantitative analyses could not be performed due to discrepancies between studies in results reporting, test stimulus and recording parameters.

Conclusions: Although abnormal results on auditory and vestibular tests were noted in PwMS, specific effects of MS on the audio-vestibular system could not be determined due to the heterogeneity between studies that restricted the ability to conduct any quantitative analyses.

For peer review only - http://bmjopen.bmj.com/site/about/guidelines.xhtml

Further research with consistent reporting, consistent stimulus and consistent recording parameters are needed in order to quantify the effects of MS on the auditory and vestibular systems.

Prospero Registration: CRD42020180094

Strengths and limitations of this study:

- This study provides a comprehensive systematic review of the literature on MS and the auditory and vestibular systems from 2000 until 21st November 2021.
- This is the only systematic review that investigated the effects of MS on the audio-vestibular system.
- Differences between studies in reporting of results and in test procedures restricted the ability to conduct any form of quantitative analyses.

Keywords: Multiple sclerosis, auditory function, vestibular function, audio-vestibular system

For peer review only

The effects of Multiple Sclerosis on the audio-vestibular system: A systematic review

INTRODUCTION

Multiple Sclerosis (MS) is a chronic, immune-mediated, inflammatory disease that causes a neurodegenerative process in the Central Nervous System (CNS).[1] There are four main types of MS: Relapsing-remitting (RRMS), Primary-Progressive (PPMS), Secondary Progressive (SPMS) and Progressive Relapsing (PRMS). The autoimmune process of MS results in CNS plaques (focal areas of demyelination) in addition to axonal injury or loss.[2,3] These plaques most commonly occur in the white matter of the brain, cerebral cortex including subpial regions, spinal cord and optic nerve,[4] resulting in optic neuritis, double vision, tremor, ataxic gait, weakness and numbness in one or more limbs.[5] It has been suggested that demyelination plaques can also occur in the central auditory and vestibular pathways resulting in hearing loss and balance disorders.[6] For example, plaques may occur in the brainstem, where both efferent and afferent auditory and vestibular pathways are present.[6] Also, the vestibular nuclei and the root entry zone of the eighth cranial nerve have been shown to be one of the most common neuroanatomic locations for inflammatory demyelination;[7] In addition, peripheral neural connections in the internal auditory canal and within the inner ear structures may be affected by demyelination resulting in peripheral auditory and vestibular involvement in persons with MS (PwMS).[8–10] The extent of occurrence of audio-vestibular symptoms in PwMS is unclear, as reports on their prevalence vary in the literature. For example, one study reported a prevalence of audio-vestibular symptoms between 1% and 28%.[11] Whereas others reported that vertigo is seen in 20 – 35 % of PwMS, and hearing loss is seen in 1 – 17 % of PwMS.[12–15] While a recent systematic review on Magnetic Resonance Imaging (MRI) and auditory tests found that 25 % of PwMS had either sudden or progressive hearing loss.[11]

Literature on the involvement of the auditory system in PwMS is either contradictory or limited. First, several studies that assessed the effects of MS on the peripheral auditory system through

For peer review only - http://bmjopen.bmj.com/site/about/guidelines.xhtml

evaluating hearing thresholds via Pure Tone Audiometry (PTA) found worse PTA thresholds in PwMS compared to healthy controls;[16–18] while one study did not find any significant differences in PTA thresholds between PwMS and healthy controls.[19] Second, some studies that assessed the effects of MS on the brainstem auditory pathway via Auditory Brainstem Responses (ABR) found that PwMS had abnormal ABRs;[11,20] while one study found no differences in ABRs between PwMS and healthy controls.[21] Finally, only a few studies investigated the effects of MS on speech understanding as assessed via Word Discrimination Scores (WDS) and on temporal processing as assessed via the Gaps-In-Noise (GIN) test. These found poorer WDS in noise and poorer performance on the GIN in PwMS compared to healthy controls.[22,23]

Literature on the involvement of the vestibular system in PwMS generally found abnormalities in PwMS. Studies that assessed the vestibular system via Electronystagmography (ENG) found abnormal results in PwMS on tests of peripheral vestibular function, central vestibular function or both.[24–27] Other studies that investigated the vestibular pathway via Vestibular Evoked Myogenic Potentials (VEMPs) in PwMS found abnormalities in VEMP results including latency delays, reduced amplitudes and inter-aural side differences.[28–39] Further studies investigated the functional integrity of the vestibular system in PwMS via static and dynamic posturography, and found that PwMS performed in general worse than healthy controls.[40–42] Although literature has shown abnormalities on different tests of vestibular function in PwMS, different studies used different test procedures, different parameters, or reported different components of each test result (e.g. VEMP latency versus amplitude).

In summary, numerous studies performed different audio-vestibular test batteries in PwMS. These studies aimed to uncover possible MS-related changes in auditory or vestibular function. However, results from literature that investigated auditory function varied, as some indicated no differences, while others reported considerable differences between PwMS and healthy

For peer review only - http://bmjopen.bmj.com/site/about/guidelines.xhtml

controls. Whereas results from literature on the effects of MS on vestibular function generally showed abnormalities in PwMS, the effects of MS on central versus peripheral vestibular function are still unclear. Therefore, literature needs to be combined and summarised to better understand the potential effects of MS on the auditory and/or vestibular systems. If auditory and/or vestibular disorders in PwMS are better understood, early identification and early intervention could occur, resulting in improved quality of life. This highlights the need for this systematic review with a focus on the effects of MS on the audio-vestibular system. We hypothesise that PwMS experience auditory and vestibular disorders at a higher rate than the general population. This systematic review aims to investigate this hypothesis by examining and synthesising current literature on the effects of MS on the audio-vestibular system.

METHODS

This systematic review followed the Preferred Reporting Items for Systematic reviews and Meta-Analyses (PRISMA) statement,[43] and was pre-registered in PROSPERO (CRD42020180094)

Patient and public involvement

No patient involved

Research ethics approval

As new participant data were not collected, Ethics Approval was not needed.

Data Sources and Searches

A systematic search was conducted in the following electronic bibliographic databases: PubMed, NICE, Scopus and Web of Science using the following terms: "Multiple Sclerosis", "Auditory Function", "Vestibular Function", "Hearing Loss", "Dizziness", "Vertigo". Operators (AND, OR) were used to narrow the search (see Supplement for detailed search

For peer review only

strategy). A total of 21 searches were performed by E.G. up until November 21st 2021. No language or date restrictions were applied.

Inclusion and exclusion criteria

Studies were eligible for inclusion if they met the following criteria: 1) investigated audio-vestibular conditions; 2) participants were diagnosed with any type of MS (RRMS, SPMS, PPMS, PRMS); 3) participants were aged 18 years and older because of maturational effects on outcomes of some audio-vestibular tests; 4) one or more of the pre-selected tests described below were used to evaluate auditory or vestibular function; 5) any of the following study types: randomised controlled trials, cohort, case-controlled, observational, or retrospective studies; 6) results from PwMS were either compared to a healthy control group or to normative data; 7) any geographical location. Studies were excluded based on the following: 1) did not include the following keywords "Multiple Sclerosis", "dizziness", "auditory function", "vestibular", "vertigo" or "hearing loss" in the title or abstract; 2) no available full text in English; 3) published before the year 2000; 4) participants were diagnosed with comorbid diseases.

Audio-vestibular tests

Evaluation of auditory function was restricted to the following measures: Pure Tone Audiometry (PTA), Auditory Brainstem Response (ABR), Otoacoustic Emissions (OAEs: Transient Evoked (TEOAE) and Distortion Product (DPOAE)), Cortical Auditory Evoked Potentials (CAEP) and Functional MRI assessing auditory function. Evaluation of vestibular function was restricted to the following measures: Vestibular Evoked Myogenic Potentials (VEMPs) including Cervical (cVEMPs) and Ocular (oVEMPs), Videonystagmography (VNG), Electonystagmography (ENG), Posturography and Rotary Chair. Above tests were defined as primary outcomes, the secondary outcomes were defined as measures of speech perception and temporal processing, specifically; Gaps in Noise test (GIN), Word Discrimination Scores

For peer review only - http://bmjopen.bmj.com/site/about/guidelines.xhtml

(WDS) and Duration Pattern Sequence Test (DPST). (Refer to supplement for details on which measures and parameters were extracted for each test).

Study selection

E.G. manually removed duplicates and screened titles and abstracts for inclusion and exclusion criteria. Remaining full texts were independently reviewed by E.G. and G.B., and any disagreements were resolved by discussion or by involving K.K. until a consensus was reached.

Assessment of bias

Risk of bias assessment was done independently by E.G. and G.B.. Case-control studies were assessed using the Risk of Bias Assessment Tool for Nonrandomized Studies (RoBANS [44]) to examine the selection of participants, confounding variables, measurement of exposure, blinding of outcome assessment, incomplete outcome of data and selective outcome reporting. Cohort studies were assessed with the Newcastle Ottawa Scale (NOS [45]). A judgement of 'low risk' of bias, 'high risk' or bias, or 'unclear risk' of bias was provided for each domain. Cross-sectional studies were evaluated with the NOS adapted for cross-sectional studies.[46] Any disagreements were resolved by discussion or by involving the third reviewer (K.K.) until a consensus was reached.

Data extraction and synthesis

The following data were extracted when available: 1) Study details: aims and design. 2) Population: recruitment methods; diagnosis and inclusion/exclusion criteria; syndrome differentiation; course of disease; number of participants: screened, eligible, enrolled, included in the analysis; age; gender. 3) Details of the pathology: specific lesion site, primary or secondary, stable or progressive, additional handicaps related to MS. 4) Methodology: test procedure, stimulus and recording parameters, electrode impendence and inter-electrode impedance levels. 5) Test results, e.g. PTA thresholds, VEMP amplitudes, ABR peak latencies, etc.

For peer review only

E.G. extracted the data and G.B. confirmed the accuracy of all extracted data. Any disagreements were resolved by discussion between the two reviewers. Per pre-registered protocol, investigators were not contacted for unreported data or additional information due to time constraints and given current COVID-19 pandemic related global circumstances.

Criteria for data synthesis: studies that used the same test procedure (either PTA, ABR or VEMP), minimum five participants in each study, minimum two studies for each test method, and the same stimulus and recording parameters were used. Mean, Standard Deviation (SD), and/or individual values were extracted when available, however, the effect size could not be calculated due to inconsistencies between studies in stimulus and recording parameters or in how results were reported.

RESULTS

Description of included studies

3876 studies were identified initially, and 1484 studies were identified after duplicate removal and primary limitations (Figure 1); 1055 were excluded because they did not relate to audio-vestibular conditions, 42 due to no full text availability, 33 were not in English, 177 due to the use of tests other than those in the inclusion criteria, and 87 were excluded because they did not have an abstract. The remaining 90 studies were examined for inclusion, eight were excluded because they were case reports, 22 did not evaluate audio-vestibular conditions, six used tests other than those in the inclusion criteria, 13 included participants under 18 years of age, and six additional duplicates were removed.

A total of 35 studies underwent full-length review. Fourteen evaluated the auditory system, 18 evaluated the vestibular system and three evaluated both auditory and vestibular systems.

Assessment of bias

Case-control studies assessed with RoBANS (Supplement Table S1) were judged as low risk of bias in most categories, except for blinding of outcome assessment that was judged as high

For peer review only

risk of bias in all 22 studies. Cohort studies assessed with NOS (Supplement Table S2) were judged as either good (*n* = 2), poor (*n* = 2), or unknown (*n* = 2, rating could not be assigned due to one or more category that was judged as "not applicable" e.g. two studies were evaluated as not applicable for "selection of the non-exposed group" due to lack of a control group). Cross-sectional studies were assessed using NOS adapted for cross-sectional (Supplement Table S3) and most were judged as good (*n* = 6) with one judged as poor.

MS and auditory function

Seventeen studies investigated the auditory systems involvement in PwMS (Table 1 – see Supplement Table S4 for an alternative table format summarizing findings based on outcome): three assessed OAEs, five assessed PTA, 13 evaluated ABRs, two investigated CAEPs, and three of the 17 studies performed speech perception and/or temporal processing tests. No studies that conducted functional MRI to assess auditory function were located.

Three studies compared OAEs in PwMS and healthy controls with a total number of 218 participants, 120 PwMS and 98 controls. Two studies did not find differences between PwMS and controls in TEOAEs and/or DPOAEs.[14,47] However, the third study found weaker responses in both TEOAEs and DPOAEs in PwMS compared to healthy controls.[21]

Five studies performed PTA on PwMS to assess hearing levels. A total of 510 participants (265 PwMS and 245 healthy controls) were evaluated with PTA, sample size varied from 80 to 146 and age range was between 18 and 65 years. Two out of five studies reported no significant difference in PTA thresholds between PwMS and controls,[19,21]. One reported worse PTA thresholds only in female PwMS compared to female controls,[13] and two reported worse thresholds in PwMS compared to controls,[18,48] with one finding worse thresholds in participants with SPMS when compared to RRMS.[18] Three of five studies provided mean and SD values of PTA thresholds (Supplement Table S5). However, reported values could not be pooled because the way that authors presented the data differed between each other. For

For peer review only - http://bmjopen.bmj.com/site/about/guidelines.xhtml

instance, Doty et al. (2012) reported average PTA thresholds across right and left ears for each frequency in male and female participants separately,[19] while Di Mauro et al. (2019) reported values for each ear separately for all participants.[21]

Thirteen studies assessed ABRs (Table 1) and included 881 participants (548 PwMS and 333 healthy controls), sample size varied from 25 to 126 and age range was between 18 and 68 years. Twelve of the 13 studies reported abnormalities in different ABR components in PwMS as follows:

- Prolonged absolute latencies of wave I[47] III and V.[47–50]
- Prolonged interpeak latencies of I–III,[14,28,30,48–53] III–V,[14,28,30,48,50–53] and I–V.[20,28,47–51,53]
- Interaural interpeak latency differences for I–III and I–V[28] and III–V.[28,51]
- Absent waves III and V.[28,30,48,51,52]
- Absent waves at high stimulus presentation rates.[54]
- Reduced wave V amplitude.[53]
- Increased I/III and I/V amplitude ratios.[51]
- Reduced V/I amplitude ratio.[48,50]
- Poor waveform morphology and/or only wave I within normal limits.[20]

Only Di Mauro et al. (2019) did not find any differences in ABRs between PwMS and controls.[21] Six out of 13 studies shared mean, and SD values for latencies, amplitudes and/or inter-aural latency differences (Supplement Table S6); however, data could not be combined, and the effect size calculation was not performed due to the variability in stimulus and recording parameters. For example, Matas et al. (2010) presented rarefaction clicks at 80 dB nHL with a presentation rate of 19.9 clicks per second,[49] while Kaytancı et al. (2016) presented clicks (polarity unspecified) at 70 dB nHL with a presentation rate of 13.0 clicks per second.[47]

For peer review only

Cortical evoked potentials were evaluated by two studies using two different tests (Table 1). Japaridze et al. (2002) reported 30% abnormality in slow cortical potentials (SCPs); abnormalities were either absent peaks or prolonged peak latencies.[51] Matas et al. (2010) assessed P300 peak latency (defined as a cognitive potential) and did not find any significant differences between PwMS and healthy controls; however, percentage of abnormalities in P300 results was higher in the PwMS (16%) compared to controls (0%).[49]

Three studies assessed speech perception and temporal processing (Table 1) with total of 188 participants, 93 PwMS and 95 healthy controls and an age range of 18-65 years. Two of the three found that PwMS had poorer WDS in noise and an increased approximate threshold plus lower percent correct answers on the GIN test compared to controls;[22,23] and one of the two also found that PwMS had lower percent correct answers on the DPST.[22] However, the third study found no significant difference between PwMS and controls on WDS in quiet.[18]

For peer review only

Table 1. *Characteristics of studies examining the effects of MS on auditory function*

Author (Year)	Study Type	Sample Size		PwMS Mean Age (years)	PwMS Age range (Years)	% Female	MS Type (sample size)	MS Duration Mean (range) years	Test Battery	Results of PwMS compared to controls / normative data
		PwMS	Healthy Controls							
Japaridze et al. (2002)[51]	Case-Control	40	33	30.5	18-57	77.5	?	?	ABR	65% Abnormal
									CAEP (SCP)	30% Abnormal
Versino et al. (2002)[28]	Cohort	65	18	35.5	19-61	?	?	?	ABR	37.5% Abnormal
Burina et al. (2008)[52]	Cohort	60	NA	37.2	?	68.3	RRMS	?	ABR	95% Abnormal
Eleftheriadou et al. (2009)[30]	Cohort	46	40	40	20-66	45.6	RRMS	4.6	ABR	26% Abnormal
Lima et al. (2009)[20]	Case-control	25	NA	42.6 (female)<br>38 (male)	33-53 (female)<br>24-56 (male)	64.0	RRMS (14), PPMS (8), Unspecified Progressive (3)	?	ABR	30% Abnormal
Lewis et al. (2010)[18]	Case-control	47	49	51.4	21-65	57.7 RRMS, 28.6 SPMS	RRMS (26), SPMS (21)	RRMS: 12.6 (2-43)<br>SPMS: 23.3 (8-50)	PTA	Worse thresholds
									WDS	No significant difference
Matas et al. (2010)[49]	Case-control	25	25	34.88	25-55	76.0	RRMS	4.25 (?)	ABR	Significantly different
									CAEP (P300)	16% Abnormal
Doty et al. (2012)[19]	Case-control	73	73	Males: 45.24<br>Females: 45.6	?	71.2	RRMS (57), PPMS (3), SPMS (6), Unspecified (7)	Males: 7.36 (?)<br>Females: 7.84 (?)	PTA	No significant difference
Saberi et al. (2012)[14]	Case-control	60	38	29.9	?	73.3	?	3.2 (?)	PTA	Worse thresholds in female PwMS
									TEOAE & DPOAE	No significant difference
									ABR	20% Abnormal
Valadbeigi et al. (2014)[22]	Case-control	26	26	28.9	18-40	?	RRMS	?	GIN	Significantly different
									WDS	Lower scores PwMS
									DPST	Significantly different
Pokryszko-Dragan et al. (2015)[53]	Case-control	86	40	39.55	19-60	72.0	?	8.57 (1-30)	ABR	Significantly different
Kaytancı et al. (2016)[47]	Cohort	20	20	31.3	?	55.0	?	?	TEOAE	No significant difference
									ABR	Significantly different
Di Mauro et al. (2019)[21]	Cohort	40	40	37	18-50	60.0	RRMS	0.8 (0.8-2)	PTA	No significant difference
									TEOAE & DPOAE	Significantly lower
									ABR	No difference
Elbeltagy et al. (2019)[23]	Case-control	20	20	37.6	30-50	?	RRMS	?	GIN	Significantly different
									WDS	Significantly lower
Delphi et al. (2021)[50]	Cross-sectional	25	25	31.43	18-45	72.0	?	?	ABR	Significantly different
Rishiq et al. (2021)[54]	Case-control	11	9	49.5	34-68	72.7	RRMS (9), PPMS (1), SPMS (1)	11.9 (2-30)	ABR	Significantly different at high click rate
Srinivasan et al. (2021)[48]	Case-control	45	45	31.77	18-50	73.3	RRMS	6.1 (5.37)	PTA	Significantly different
									ABR	Significantly different and 55.56 % abnormal

Abbreviations: PTA= Pure Tone Audiometry, ABR= Auditory Brainstem Response, OAE= Oto-acoustic Emissions, TEOAE= Transient Evoked Oto acoustic emission, DPOAE= Distortion Product Oto acoustic Emissions, CAEP= Cortical Auditory Evoked Potentials, SCP= Slow Cortical Potentials, GIN= Gaps in Noise test, WDS= Word Discrimination Scores, DPST= Duration pattern sequence test, MS= Multiple Sclerosis, PwMS = Persons with Multiple Sclerosis, ? = not reported, NA = Not Applicable.
Cells shaded in grey represent test results that were abnormal or showed a difference between PwMS and healthy controls

For peer review only

For peer review only - http://bmjopen.bmj.com/site/about/guidelines.xhtml

MS and vestibular function

A total of 21 studies that investigated the vestibular system in PwMS were identified (Table 2 – see Supplement Table S7 for an alternative table format summarizing findings based on outcome). Two studies investigated vestibular function using ENG; three used static posturography, one used dynamic posturography, 14 used cVEMPs and four also used oVEMPs, and two used Rotary Chair testing.

A total of 90 participants (60 PwMS and 30 healthy controls) were evaluated using ENG, age ranged between 24 and 64. Both studies found ENG abnormalities in the peripheral and central vestibular systems in PwMS.[27,55] Degirmenci et al. (2010) noted that 90% of PwMS showed ENG abnormalities compared to 6.7% of the controls, and there was a significant difference between groups.[27] Abnormalities suggestive of central vestibular pathology were found in 83.3% of PwMS and in 6.7% of controls, and abnormalities suggestive of peripheral vestibular pathology were found in 36.7% of PwMS and in 0% of controls.[27] Zeigelboim et al. (2008) also reported ENG abnormalities in 86.7% (83.4% Peripheral) of their PwMS.[55]

Four studies assessed posturography with a total of 314 participants (187 PwMS and 127 healthy controls). All four studies found significant differences between PwMS and controls.[40,41,56,57] Two studies reported static posturography results for the same sample,[40,41] they noted increased delineated area, average sway and speed of sway for both open and closed eyes in PwMS compared to controls. A third study also conducted static posturography and found that sway area and path length were significantly greater in PwMS than in controls.[56] The fourth study evaluated dynamic posturography and found longer motor control test latencies, lower equilibrium scores on the sensory organisation tests, and a compromised ability to use three sensory inputs (somatosensory, vestibular, and visual preference) in PwMS compared to controls.[57]

For peer review only

A total of 14 studies assessed VEMPs and included 724 participants (435 PwMS and 289 healthy controls), sample size varied from 30 to 88, and age range was between 18 and 71 years. Four studies evaluated both oVEMPs and cVEMPs[35,58–60] and 10 studies evaluated cVEMPs.[28–30,50,61–66] Thirteen of the 14 studies reported abnormalities in different VEMP components in PwMS as follows:

- Prolonged latencies of p13,[28–30,35,50,58,60–66] n23,[28,30,35,50,58,60–66] and p1 and n1.[35,58,60]
- Reduced p13-n23 amplitude.[28,50,61,63]
- Inter-side asymmetry for p13,[28,29,63] and n23 and n23 latencies.[28]
- Absent waves.[30,62,63,66]

Ten of 14 studies compared VEMPs in PwMS to healthy controls.[30,35,50,58–63,65] Except for two,[59,65] all found a significant difference between PwMS and controls. The remaining VEMP studies used data from controls as normative data.[28,29,64,66]

Thirteen studies shared mean and SD values of p13, n23, p1 and n1 latencies (Supplement Tables S8 and S9). However, data could not be analysed because intensity level, stimulus type, stimulus polarity and other VEMP recording parameters differed between studies. For instance, Sartucci and Logi (2002) presented clicks at 140 dBSPL at a rate of three per second,[61] Aidar and Suzuki (2005) presented clicks at 95 dBHL at a rate of two per second,[62] Kavasoğlu et al. (2017) presented clicks at 100 dBnHL at a rate of five per second,[65] and Koura and Hussein (2018) presented a 500-Hz tone burst at 95 dBHL.[66]

Two studies conducted Rotary Chair testing. However, they tested and reported results from the same sample.[59,67] Forty PwMS and 20 healthy controls were tested, results showed that PwMS had lower vestibular-ocular reflex (VOR) cancellation gain with a visual target and larger variances in their responses on both the subjective visual vertical and horizontal (SVV/H)

For peer review only

tests compared to controls.[59] Also, that PwMS performed worse than controls on central vestibular measures but not on peripheral.[67]

For peer review only

For peer review only - http://bmjopen.bmj.com/site/about/guidelines.xhtml

Table 2. *Characteristics of studies examining the effects of MS on vestibular function*

Author (Year)	*Study Type*	*Sample Size*		*Mean Age PwMS (years)*	*PwMS Age range (Years)*	*% Female*	*MS Type (sample size)*	*MS Duration Mean (range) years*	*Test Battery*	*Results of PwMS compared to controls / normative data*
		PwMS	*Healthy Controls*							
Sartucci & Logi (2002)[61]	Case-Control	15	15	44.5	26-59	66.6	?	?	cVEMP	Significantly different
Versino et al. (2002)[28]	Cohort	70	18	35.5	19-61	?	?	?	cVEMP	31.4% Abnormal
Alpini et al. (2004)[29]	Case-control	40	25	38	17-71	57.5	?	?	cVEMP	70% Abnormal
Aidar and Suzuki (2005)[62]	Case-control	15	15	39.3	?	?	?	?	cVEMP	Significantly different
Patkó et al. (2007)[63]	Case-control	30	30	43.4	27-60	66.6	?	?	cVEMP	Significantly different
Zeigelboim et al. (2008)[55]	Case-control	30	0	42.23	27-64	80	RRMS	?	ENG	86.7% Abnormal
Eleftheriadou et al. (2009)[30]	Cohort	46	40	40	20-66	45.6	RRMS	4.6 (?)	cVEMP	Significantly different
Degirmenci et al. (2010)[27]	Cohort	30	30	37.9	23-56	56.7	RRMS	?	ENG	90% Abnormal
Gabelić et al. (2013)[58]	Case control	30	15	?	?	46.6	RRMS	3.93 (0.2-21)	cVEMP<br>oVEMP	Significantly different<br>Significantly different
Harirchian et al. (2013)[64]	Case-control	20	20	30	20-40	50	RRMS<br>SPMS	?	cVEMP	70% Abnormal
Parsa et al. (2015)[35]	Cross-sectional	34	15	29.8	?	100	?	?	cVEMP<br>oVEMP	Significantly different<br>Significantly different
Doty et al. (2018)[57]	Case-control	58	72	Males:44.61<br>Females: 44.60	?	68.9	?	Male: 7.03 (?)<br>Female: 6.54 (?)	Dynamic Posturography	Significantly different
Kavasoğlu et al. (2018)[65]	Case-control	30	31	30	18-45	60.0	?	< 1 (?)	cVEMP	23.3% Abnormal<br>No significant difference
Koura and Hussein (2018)[66]	Case-control	20	10	36.80	?	65.0	?	4.4 (?)	cVEMP	100% Abnormal
Inojosa et al. (2020)[40,41]	Cross-sectional	99	30	35.01	18-50	68.7	?	5.5 (?)	Static Posturography	Significantly different
Yang and Liu (2020)[56]	Cross-sectional	30	25	50.8	?	76.7	?	14 (?)	Static Posturography	Significantly different
Delphi et al. (2021)[50]	Cross-sectional	25	25	31.43	18-45	72.0	?	?	cVEMP	Significantly different
Elmoazen et al. (2021)[60]	Case-control	20	10	Brainstem lesions: 40<br>No Brainstem lesion: 34.1	?	?	?	?	cVEMP<br>oVEMP	Significantly different<br>Significantly different
Cochrane et al. (2021)[59,67]	Cross-sectional	40	20	42.4	21-55	88.0	RRMS	9.9	Rotary Chair<br>cVEMP<br>oVEMP	Significantly different<br>No significant difference<br>No significant difference

Abbreviations: cVEMP = Cervical Vestibular Evoked Myogenic Potentials, oVEMP = Ocular Vestibular Evoked Myogenic Potentials, MS= Multiple Sclerosis, PwMS = Persons with Multiple Sclerosis, ? = not reported.
Cells shaded in grey represent test results that were abnormal or showed a difference between PwMS and healthy controls.

For peer review only

For peer review only - http://bmjopen.bmj.com/site/about/guidelines.xhtml

DISCUSSION

In this systematic review, 35 studies were reviewed: 22 case-control studies, six cohort studies, and seven cross-sectional studies. The aim was to investigate the effects of MS on the audio-vestibular system by examining and synthesising current literature that used different auditory and vestibular tests. Additionally, this systematic review presents an overview of the last 21 years of literature specific to MS in the context of the auditory and vestibular systems. The selected outcome measures for the auditory system were: OAE, PTA, ABR, CAEP, WDS, GIN, DPST. Vestibular system outcome measures were: VNG, ENG, Rotary Chair, VEMPs, Static and dynamic Posturography. The included studies on auditory function evaluated 1215 individuals in total, 714 PwMS and 501 healthy controls, the included studies on vestibular function covered 1128 individuals, 682 PwMS and 446 healthy controls. Included studies assessed participants who were over the age of 18, and the geographical location of individuals was not limited to a specific continent, country or hemisphere.

Neither meta-analyses nor effect size calculations could be performed due to the inconsistencies across studies in reporting results or in test stimulus and recording parameters that lead to the inability to combine their data (Supplement Tables S5, S6, S8, S9). This is a significant methodological limitation especially that the impact of this systematic review on clinical practice would have been much greater had quantitative analyses been performed.

Auditory system and MS

The specific effects of MS on the auditory system, based on results of auditory tests included in this systematic review, could not be established.

OAEs in PwMS

Effect of MS on the peripheral auditory system, as demonstrated by OAEs, was only found by one of the three included studies.[21] Di Mauro et al. (2019) reported abnormal OAEs with normal ABRs in participants newly diagnosed with RRMS, showing cochlear rather than

For peer review only - http://bmjopen.bmj.com/site/about/guidelines.xhtml

retrocochlear involvement.[21] They suggested that this cochlear dysfunction may be caused by a demyelinating process in the brainstem's medial olivocochlear bunch that innervates outer hair cells[68] resulting in decreased OAE amplitudes.[21] However, reduced OAE amplitudes were not reported by others, and it is unclear why Di Mauro et al. (2019) found differences between PwMS and controls, and both Kaytancı et al. (2016) and Saberi et al. (2012) did not.[14,47] Therefore, there is very limited evidence to date on the effects of MS on OAEs, and there is need for further investigations.

PTA in PwMS

Effects of MS on PTA thresholds could not be defined. Three of five included studies reported worse PTA thresholds in PwMS compared to controls.[14,18,48] However, Srinivasan et al. (2021) included only participants with normal hearing, i.e. test sample was biased towards better PTA,[48] therefore the difference between PwMS and controls, although statistically significant, is not clinically significant. Apart from Di Mauro et al. (2019), who found no difference between PwMS and controls but only included participants with normal hearing that were recently diagnosed with RRMS,[21] reasons behind differences in PTA results between studies are unclear. Type and duration of MS do not appear to be factors, as those who found a difference included participants with a mixture of MS types and MS durations. For example, Lewis et al. (2010) included participants (n = 47) with RRMS (mean MS duration: 12.6 years) and SPMS (mean MS duration: 23.3 years),[18] and Saberi et al. (2012) included PwMS (n = 60, MS type not reported) with a mean MS duration of 3.2 years,[14] and they both found differences in PTA thresholds between PwMS and controls. While Doty et al. (2012) did not find any difference in PTA thresholds with the inclusion of participants (n = 73) with different MS types (RRMS, PPMS, SPMS, Unspecified MS) and a mean MS duration of 7.36 years in males and 7.84 years in females.[19] Therefore, conclusions on the effects of MS on PTA thresholds cannot be reached given the limited literature and the inability to combine results for

For peer review only

For peer review only - http://bmjopen.bmj.com/site/about/guidelines.xhtml

a meta-analysis. Thus, there is a clear need for further studies investigating changes in PTA thresholds in PwMS.

ABRs in PwMS

The effects of MS on the brainstem auditory pathway as demonstrated by ABRs were more evident; 12 of 13 included studies found abnormalities in different ABR components in PwMS.[14,20,28,30,47–54] Demyelination plaques tend to occur in the brainstem, and the impairment of the auditory olivocochlear pathways by demyelination is possible.[6] Therefore, ABR abnormalities such as prolonged latencies are thought to be related to the effects of MS plaques in the brainstem on the integrity of the auditory pathways.[11] Abnormal ABR findings in PwMS reported by studies included in this review were consistent with previous literature.[25,69] Only Di Mauro et al. (2019) found no differences in ABRs between PwMS and controls; however, PwMS with hearing loss or brainstem lesions in their MRI were excluded from the study, plus the authors included only participants newly diagnosed with RRMS.[21] Matas et al. (2010) included normally hearing participants with RRMS (mean MS duration: 4.25 years) and found differences in ABRs between PwMS and controls.[49] Burina et al. (2008) also included participants with RRMS and brainstem or cerebellum lesions and found abnormal ABRs.[52] Therefore, the reasons Di Mauro et al. (2019) did not find differences in ABRs between PwMS and controls are likely related to the short duration of MS and the exclusion of participants with brainstem lesions. Although most studies that investigated ABRs in PwMS found abnormalities, the nature of these abnormalities could not be established. This is due to the differences between studies in reported ABR measures (e.g. absolute peak latencies versus peak amplitudes) and in stimulus and recording parameters (e.g. presentation levels, stimulus polarity, transducer). Therefore, there is a clear need for consistency across studies to better understand the effects of MS on ABRs.

For peer review only - http://bmjopen.bmj.com/site/about/guidelines.xhtml

375 CAEPs in PwMS

376 The effects of MS plaques on the integrity of the central auditory pathway as measured by
377 CAEPs was much less studied, only two studies were included in this systematic review.[49,51]
378 Both found abnormalities suggesting possible cortical involvement in PwMS; however, these
379 studies could not be compared, and data could not be synthesised due to the use of different
380 testing techniques. Therefore, further studies are needed to better understand the effects of MS
381 on CAEPs.

382 Speech perception and temporal processing in PwMS

383 The effects of MS on speech perception and temporal processing were also infrequently studied,
384 three studies were included in this systematic review.[18,22,23] Two of the three found
385 abnormalities on WDS tests in PwMS, both studies that found abnormalities performed WDS
386 testing in noise,[22,23] and the study that did not find abnormal WDS tested in quiet.[18] It is
387 therefore likely that Lewis et al. (2010) did not find abnormal WDS in PwMS because they did
388 not test in background noise.[18] This is supported by Valadbeigi et al.'s (2014) findings of
389 worse WDS in noise than in quiet in PwMS.[22] These abnormalities in WDS are possibly
390 related to MS lesions in the central auditory pathways. Any degeneration caused by MS plaques
391 could lead to a misperception of the acoustic signal leading to poorer performance on WDS.[70]
392 Additionally, the two studies that performed the GIN test found abnormalities in PwMS
393 indicating lower temporal resolution performance than in controls.[22,23] A previous study
394 suggested that persons with lesions in the central auditory system had worse temporal resolution
395 performance and therefore poorer performance on the GIN test compared to healthy
396 controls.[71] Therefore, it is possible that temporal resolution is affected by MS plaques in the
397 CNS. Since the evidence regarding abnormalities in speech perception and temporal processing
398 in PwMS was limited, this systematic review concludes that there is need for further research
399 to evaluate MS's effect on functional auditory performance.

For peer review only - http://bmjopen.bmj.com/site/about/guidelines.xhtml

Vestibular System and MS

All studies included in this systematic review found abnormalities on vestibular tests suggesting that the vestibular system may be more affected by MS than the auditory system.

ENGs in PwMS

The two included studies that performed ENGs in PwMS found abnormalities suggestive of peripheral and/or central vestibular pathology.[27,55] Zeigelboim et al. (2008) primarily found peripheral vestibular pathology (83.4%) in their participants with RRMS,[55] while Degirmenci et al. (2010) mainly found central vestibular pathology (83.3%) in their participants with RRMS.[27] These contradictory results might be due to the inclusion of participants with RRMS in both studies, as persons with RRMS may differ in terms of lesion site in the CNS, severity of impairment, developmental stage of disease and frequency of attacks.[55] Given this limited and contradictory evidence, the effects of MS on ENGs are still unknown. Therefore, more literature is needed on different MS subtypes to better understand this.

Posturography in PwMS

The four included studies that investigated postural control using either static or dynamic posturography found abnormal posturography results in PwMS.[40,41,56,57] To maintain balance, the CNS evaluates the input from the vestibular, visual and somatosensory systems, then weighs the input according to where reliable information is coming from, and MS may affect each of these three systems.[56] Maintaining body balance in PwMS requires increasing the weight on the other two systems if one sensory system input is impaired. Yang and Liu (2020) concluded that PwMS rely mostly on cues from visual and proprioceptive systems to maintain their balance.[56] Lord et al. (1991) and Doty et al. (2018) also stated that PwMS had difficulty maintaining their balance when visual and proprioceptive feedback were inconsistent or conflicting.[57,72] In addition, a previous study reported that PwMS have slower somatosensory conduction than healthy individuals.[73] Thus, PwMS might receive impaired

For peer review only

For peer review only - http://bmjopen.bmj.com/site/about/guidelines.xhtml

or delayed proprioceptive feedback resulting in postural instability.[56,73,74] Another possible cause of instability in PwMS is that motor neurons might be affected by demyelination plaques, leading to an impairment in the transmission from the CNS to the motor end units.[56,75] Although, all four included studies found greater instability and impaired balance in PwMS, the evidence regarding the effects of MS on functional balance outcomes is very limited. Further research is required to make a clear conclusion on this subject.

VEMPs in PwMS

Thirteen of the 14 included studies that assessed VEMPs reported abnormalities in PwMS. Significant differences between PwMS and controls were reported by eight of the ten studies that compared the two groups.[30,35,50,58,60–63] Both Kavasoğlu et al. (2018) and Cochrane et al. (2021) did not find differences between PwMS and controls.[59,65] However, Kavasoğlu et al. (2018) found VEMP latency delays exceeding 2.5 SD from normative data in 23% of their PwMS;[65] Cochrane et al. (2021) did not report whether any VEMP abnormalities were found in their PwMS.[59] Kavasoğlu et al. (2018) included PwMS that were recently diagnosed (within less than one year), this may be the reason why they did not find differences between PwMS and controls.[65] Cochrane et al. (2021) attributed the lack of difference between PwMS and controls to the fact that their participants had low scores on the disability scale indicating that they were less likely to have lesions affecting VEMP results.[59] However, Gabelić et al. (2013) found differences between PwMS and controls and their participants had similar disability scale scores to Cochrane et al. (2021).[58] The remaining studies that reported percent PwMS that had abnormal VEMPs found various results. For example, Alpini et al. (2004) found abnormalities in 70 %[29] Versino et al. (2002) found abnormalities in 31.4 %[28] and Koura and Hussein (2018) found abnormalities in 100 % of PwMS who participated in their study.[66] The majority did not report type or duration of MS, or severity of impairment, therefore, it is difficult to speculate why some studies reported higher percentages of VEMP abnormalities in

For peer review only

For peer review only - http://bmjopen.bmj.com/site/about/guidelines.xhtml

PwMS than others. The most common reported cVEMP abnormalities were prolonged p13 and n23 latencies[28–30,35,58,61–66] and the most common reported oVEMP abnormalities were prolonged p1 and n1 latencies.[35,58] Prolonged latencies may be a result of inflammatory demyelination in the vestibular nuclei and the root entry zone of the eighth cranial nerve[7] leading to reduced neural transmission speed and consequently prolonged latencies. Additionally, Aidar and Suzuki (2005), and Koura and Hussein (2018) noted the absence of waves in 30 % and 40 % of PwMS,[62,66] respectively, which may be caused by a lesion in the vestibulospinal tract.[76] Although all studies showed VEMP abnormalities in PwMS, the nature of these abnormalities and their relationships to MS could not be established. This is due to the differences between studies in VEMP stimulus and recording parameters (e.g. presentation levels, stimulus polarity, transducer), resulting in the inability to combine data for a meta-analysis. In addition, type, duration, and severity of MS were not consistently reported. Therefore, there is a clear need for consistency across studies to better understand the effects of MS on the vestibular system by using VEMPs.

Rotary Chair testing in PwMS

The two studies that reported results on Rotary Chair testing from the same participants found differences that indicate central rather than peripheral vestibular impairments in PwMS compared to controls.[59,67] They suggested that central integration of peripheral input is what is impaired in PwMS.[59,67] However, more studies are needed to confirm these findings.

CLINICAL IMPLICATIONS

This systematic review clearly demonstrates that both peripheral and central audio-vestibular functions may be affected by MS plaques and by the MS inflammatory process. This suggests that a comprehensive audio-vestibular test battery should be incorporated in the assessment protocols of PwMS. For auditory function, this review found that ABRs were the most sensitive measure in PwMS; therefore, ABRs should be included in the test battery. OAEs and PTA

For peer review only - http://bmjopen.bmj.com/site/about/guidelines.xhtml

should also be included given that there is some evidence that the inner ear may be affected by the MS inflammatory process. For vestibular function, this review found that all tests are affected in PwMS; therefore, ENG/VNG, VEMPs, Rotary Chair, and Posturography should be included in the battery.

CONCLUSIONS

This systematic review aimed at understanding the effects of MS on the auditory and vestibular systems. No previous review that investigated this relationship was found. In this systematic review, an effect of MS on the auditory system was consistently found in the results of ABRs, CAEPs, WDS and GIN tests, but not in OAEs or PTAs. Indicating that the brainstem and central auditory systems were more likely to be affected by MS than the peripheral auditory system. Furthermore, an effect of MS on the vestibular system was consistently found in the results of ENG, Dynamic and Static Posturography and VEMPs. This suggests that both the peripheral and central vestibular systems may be involved in the degenerative process of MS. However, quantitative analyses could not be performed in this systematic review due to inconsistencies across studies in reporting of results, in test procedures and in test stimulus and recording parameters. Therefore, although abnormal results in PwMS on both auditory and vestibular tests were identified, further studies are necessary to quantify this effect and obtain more robust evidence on the effects of MS on the auditory and vestibular systems. Future studies should use consistent test parameters and account for confounding factors such as type, duration and severity of MS.

Figure Legend:

Figure 1. Flow diagram of systematic literature review

For peer review only

Contributorship Statement: E.G and G.B equally contributed to this work (co-first authors). EG, G.B and K.K conceptualized, planned and designed this systematic review, E.G conducted the searches and initial screening of titles and abstracts. E.G and G.B independently reviewed full texts and conducted assessment of bias, E.G extracted data and this was verified by G.B, disagreements at any stage were resolved by discussion and by involvement of K.K.

E.G and G.B interpreted the results and this was reviewed by K.K.

E.G and G.B wrote the original manuscript and also reviewed and edited the manuscript. K.K was responsible for supervision, administration, and reviewing and editing the manuscript.

Funding: This research received no specific grant from any funding agency in the public, commercial or not-for-profit sectors. KK was supported by the NIHR Manchester Biomedical Research Centre, UK IS-BRC-1215-20007 and MRC Programme Grant MR/L003589/1.

Competing interest: No competing interests.

Data Sharing: No additional data available.

For peer review only

For peer review only - http://bmjopen.bmj.com/site/about/guidelines.xhtml

REFERENCES

1 Sospedra M, Martin R. Immunology of Multiple Sclerosis. *Annu Rev Immunol* 2005;**23**:683–747. doi:10.1146/annurev.immunol.23.021704.115707

2 Gold R, Linington C, Lassmann H. Understanding pathogenesis and therapy of multiple sclerosis via animal models: 70 years of merits and culprits in experimental autoimmune encephalomyelitis research. *Brain* 2006;**129**:1953–71. doi:10.1093/brain/awl075

3 Trapp BD, Peterson J, Ransohoff RM, *et al.* Axonal Transection in the Lesions of Multiple Sclerosis. *New Engl J Medicine* 1998;**338**:278–85. doi:10.1056/nejm199801293380502

4 Popescu BFGh, Lucchinetti CF. Pathology of Demyelinating Diseases. *Annu Rev Pathology Mech Dis* 2012;**7**:185–217. doi:10.1146/annurev-pathol-011811-132443

5 Rolak LA. Multiple Sclerosis: It's Not The Disease You Thought It Was. *Clin Medicine Res* 2003;**1**:57–60. doi:10.3121/cmr.1.1.57

6 Coelho A, erani B, Prasher D, *et al.* Auditory Efferent Function Is Affected in Multiple Sclerosis. *Ear Hearing* 2007;**28**:593–604. doi:10.1097/aud.0b013e31812f716e

7 Brandt T, Dieterich M. Preliminary Classification of Vestibular Brain-Stem Disorders. In: L.R. C, H.C. H, eds. *Brain-Stem Localization and Function*. Berlin, Heidelberg: : Springer 1993. 79–91. doi:10.1007/978-3-642-78172-8_9

8 Bogie JFJ, Stinissen P, Hendriks JJA. Macrophage subsets and microglia in multiple sclerosis. *Acta Neuropathol* 2014;**128**:191–213. doi:10.1007/s00401-014-1310-2

9 O'Malley JT, Nadol JB, McKenna MJ. Anti CD163+, Iba1+, and CD68+ Cells in the Adult Human Inner Ear. *Otol Neurotol* 2016;**37**:99–108. doi:10.1097/mao.0000000000000879

10 Di Stadio AD, Ralli M. Inner ear involvement in multiple sclerosis: An underestimated condition? *Mult Scler J* 2018;**24**:1264–5. doi:10.1177/1352458517750010

11 Di Stadio AD, Dipietro L, Ralli M, *et al.* Sudden hearing loss as an early detector of multiple sclerosis: a systematic review. *Eur Rev Med Pharmaco* 2018;**22**:4611–24. doi:10.26355/eurrev_201807_15520

12 Franklin DJ, Coker NJ, Jenkins HA. Sudden Sensorineural Hearing Loss as a Presentation of Multiple Sclerosis. *Archives Otolaryngology Head Neck Surg* 1989;**115**:41–5. doi:10.1001/archotol.1989.01860250043023

13 Drulović B, Ribarić-jankes K, Kostić V, *et al.* Multiple Sclerosis as the Cause of Sudden 'Pontine' Deafness. *Int J Audiol* 1994;**33**:195–201. doi:10.3109/00206099409071880

For peer review only - http://bmjopen.bmj.com/site/about/guidelines.xhtml

14 Saberi A, Hatamian HR, Nemati S, *et al.* Hearing statement in multiple sclerosis: a case control study using auditory brainstem responses and otoacoustic emissions. *Acta Medica Iranica* 2012;**50**:679–83.

15 Furst M, Levine RA. Hearing disorders in multiple sclerosis. *Handb Clin Neurology* 2015;**129**:649–65. doi:10.1016/b978-0-444-62630-1.00036-6

16 Dayal VS, Swisher LP. Pure tone thresholds in multiple sclerosis: A further study. *Laryngoscope* 1967;**77**:2169–77. doi:10.1288/00005537-196712000-00007

17 Cohen M, Rudge P. The Effect of Multiple Sclerosis on Pure Tone Thresholds. *Acta Oto-laryngol* 1984;**97**:291–5. doi:10.3109/00016488409130991

18 Lewis MS, Lilly DJ, Hutter MM, *et al.* Audiometric hearing status of individuals with and without multiple sclerosis. *J Rehabilitation Res Dev* 2010;**47**:669–78. doi:10.1682/jrrd.2010.01.0010

19 Doty RL, Tourbier I, Davis S, *et al.* Pure-Tone Auditory Thresholds Are Not Chronically Elevated in Multiple Sclerosis. *Behav Neurosci* 2012;**126**:314–24. doi:10.1037/a0027046

20 Lima TMA, Crato AN, Mancini PC, *et al.* Alterations in early auditory evoked potentials in multiple sclerosis patients. *Braz J Otorhinolar* 2009;**75**:177–81. doi:10.1016/s1808-8694(15)30775-8

21 Di Mauro RD, Di Girolamo SD, Ralli M, *et al.* Subclinical cochlear dysfunction in newly diagnosed relapsing-remitting multiple sclerosis. *Mult Scler Relat Dis* 2019;**33**:55–60. doi:10.1016/j.msard.2019.05.020

22 Valadbeigi A, Weisi F, Rohbakhsh N, *et al.* Central auditory processing and word discrimination in patients with multiple sclerosis. *Eur Arch Oto-rhino-l* 2014;**271**:2891–6. doi:10.1007/s00405-013-2776-6

23 Elbeltagy R, Gad N, Ismail M. Central auditory function in multiple sclerosis patients. *Indian J Otology* 2019;**25**:90–6. doi:10.4103/indianjotol.indianjotol_80_18

24 Noffsinger D, Olsen WO, Carhart R, *et al.* Auditory and vestibular aberrations in multiple sclerosis. *Acta Oto-laryngologica Suppl* 1972;**303**:1–63.

25 Grénman R. Involvement of the audiovestibular system in multiple sclerosis. An otoneurologic and audiologic study. *Acta Oto-laryngologica Suppl* 1985;**420**:1–95.

26 Cipparrone L, Fratiglioni L, Siracusa G, *et al.* Electronystagmography in the diagnosis of multiple sclerosis. *Acta Neurol Scand* 1989;**80**:193–200. doi:10.1111/j.1600-0404.1989.tb03862.x

27 Degirmenci E, Bir LS, Ardic FN. Clinical and electronystagmographical evaluation of vestibular symptoms in relapsing remitting multiple sclerosis. *Neurol Res* 2010;**32**:986–91. doi:10.1179/016164110x12681290831405

For peer review only

For peer review only - http://bmjopen.bmj.com/site/about/guidelines.xhtml

28 Versino M, Colnaghi S, Callieco R, *et al.* Vestibular evoked myogenic potentials in multiple sclerosis patients. *Clin Neurophysiol* 2002;**113**:1464–9. doi:10.1016/s1388-2457(02)00155-4

29 Alpini D, Pugnetti L, Caputo D, *et al.* Vestibular evoked myogenic potentials in multiple sclerosis: clinical and imaging correlations. *Mult Scler* 2004;**10**:316–21. doi:10.1191/1352458504ms1041oa

30 Eleftheriadou A, Deftereos SN, Zarikas V, *et al.* The diagnostic value of earlier and later components of Vestibular Evoked Myogenic Potentials (VEMP) in Multiple Sclerosis. *J Vestib Res* 2009;**19**:59–66. doi:10.3233/ves-2009-0342

31 Gazioglu S, Boz C. Ocular and cervical vestibular evoked myogenic potentials in multiple sclerosis patients. *Clin Neurophysiol* 2012;**123**:1872–9. doi:10.1016/j.clinph.2012.01.022

32 Güven H, Bayır Ö, Aytaç E, *et al.* Vestibular-evoked myogenic potentials, clinical evaluation, and imaging findings in multiple sclerosis. *Neurol Sci* 2014;**35**:221–6. doi:10.1007/s10072-013-1483-9

33 Crnošija L, Skorić MK, Gabelić T, *et al.* Correlation of the VEMP score, ambulation and upper extremity function in clinically isolated syndrome. *J Neurol Sci* 2015;**359**:197–201. doi:10.1016/j.jns.2015.10.049

34 Gabelić T, Skorić MK, Adamec I, *et al.* The vestibular evoked myogenic potentials (VEMP) score: a promising tool for evaluation of brainstem involvement in multiple sclerosis. *Eur J Neurol* 2015;**22**:261-e21. doi:10.1111/ene.12557

35 Parsa MS, Mohammadkhani G, Hajabolhassani F, *et al.* Cervical and ocular vestibular evoked myogenic potentials in multiple sclerosis participants. *Medical J Islamic Repub Iran* 2015;**29**:164.

36 Crnošija L, Skorić MK, Gabelić T, *et al.* Vestibular evoked myogenic potentials and MRI in early multiple sclerosis: Validation of the VEMP score. *J Neurol Sci* 2017;**372**:28–32. doi:10.1016/j.jns.2016.11.028

37 Di Stadio AD, Dipietro L, Ralli M, *et al.* The role of vestibular evoked myogenic potentials in multiple sclerosis-related vertigo. A systematic review of the literature. *Mult Scler Relat Dis* 2019;**28**:159–64. doi:10.1016/j.msard.2018.12.031

38 Shimizu K, Murofushi T, Sakurai M, *et al.* Vestibular evoked myogenic potentials in multiple sclerosis. *J Neurology Neurosurg Psychiatry* 2000;**69**:276–7. doi:10.1136/jnnp.69.2.276

39 Murofushi T, Shimizu K, Takegoshi H, *et al.* Diagnostic Value of Prolonged Latencies in the Vestibular Evoked Myogenic Potential. *Archives Otolaryngology Head Neck Surg* 2001;**127**:1069–72. doi:10.1001/archotol.127.9.1069

40 Inojosa H, Schriefer D, Klöditz A, *et al.* Balance Testing in Multiple Sclerosis—Improving Neurological Assessment With Static Posturography? *Front Neurol* 2020;**11**:135. doi:10.3389/fneur.2020.00135

For peer review only

For peer review only - http://bmjopen.bmj.com/site/about/guidelines.xhtml

41 Inojosa H, Schriefer D, Trentzsch K, *et al.* Visual Feedback and Postural Control in Multiple Sclerosis. *J Clin Medicine* 2020;**9**:1291. doi:10.3390/jcm9051291

42 Prosperini L, Petsas N, Raz E, *et al.* Balance deficit with opened or closed eyes reveals involvement of different structures of the central nervous system in multiple sclerosis. *Mult Scler J* 2014;**20**:81–90. doi:10.1177/1352458513490546

43 Page MJ, McKenzie JE, Bossuyt PM, *et al.* The PRISMA 2020 statement: an updated guideline for reporting systematic reviews. *Bmj* 2021;**372**:n71. doi:10.1136/bmj.n71

44 Kim SY, Park JE, Lee YJ, *et al.* Testing a tool for assessing the risk of bias for nonrandomized studies showed moderate reliability and promising validity. *J Clin Epidemiol* 2013;**66**:408–14. doi:10.1016/j.jclinepi.2012.09.016

45 Wells G, Shea B, O'Connell D, *et al.* The Newcastle-Ottawa Scale (NOS) for assessing the quality of nonrandomised studies in meta-analyses. Ottawa: Ottawa Hospital Research Institute. http://www.ohri.ca/programs/clinical_epidemiology/oxford.asp (accessed 2 Jul 2020).

46 Moskalewicz A, Oremus M. No clear choice between Newcastle–Ottawa Scale and Appraisal Tool for Cross-Sectional Studies to assess methodological quality in cross-sectional studies of health-related quality of life and breast cancer. *J Clin Epidemiol* 2020;**120**:94–103. doi:10.1016/j.jclinepi.2019.12.013

47 Kaytancı E, Ozdamar OI, Acar GO, *et al.* Evaluation of transiently evoked otoacoustic emissions and auditory brainstem responses in patients with multiple sclerosis. *Ear Nose Throat J* 2016;**95**:E12–7.

48 Srinivasan VS, Krishna R, Munirathinam BR. Effectiveness of Brainstem Auditory Evoked Potentials Scoring in Evaluating Brainstem Dysfunction and Disability Among Individuals With Multiple Sclerosis. *Am J Audiol* 2021;**30**:255–65. doi:10.1044/2020_aja-20-00155

49 Matas CG, Matas SL de A, Oliveira CRS de, *et al.* Auditory evoked potentials and multiple sclerosis. *Arq Neuro-psiquiat* 2010;**68**:528–34. doi:10.1590/s0004-282x2010000400010

50 Delphi M, Sayaf M, Taheri F, *et al.* Audio and vestibular analyses in patient with multiple sclerosis: a case–control study. *Hear Balance Commun* 2021;**19**:1–6. doi:10.1080/21695717.2021.1933318

51 Japaridze G, Shakarishvili R, Kevanishvili Z. Auditory brainstem, middle-latency, and slow cortical responses in multiple sclerosis. *Acta Neurol Scand* 2002;**106**:47–53. doi:10.1034/j.1600-0404.2002.01226.x

52 Burina A, Sinanović O, Smajlović D, *et al.* Some Aspects of Balance Disorder in Patients with Multiple Sclerosis. *Bosnian J Basic Med* 2008;**8**:80–5. doi:10.17305/bjbms.2008.3003

For peer review only

For peer review only - http://bmjopen.bmj.com/site/about/guidelines.xhtml

53 Pokryszko-Dragan A, Bilinska M, Gruszka E, *et al.* Assessment of visual and auditory evoked potentials in multiple sclerosis patients with and without fatigue. *Neurol Sci* 2015;**36**:235–42. doi:10.1007/s10072-014-1953-8

54 Rishiq D, Harkrider A, Springer C, *et al.* Click-evoked and speech-evoked auditory brainstem responses from individuals with multiple sclerosis. *Neurosci Lett* 2021;**740**:135460. doi:10.1016/j.neulet.2020.135460

55 Zeigelboim BS, Arruda WO, Mangabeira-Albernaz PL, *et al.* Vestibular findings in relapsing, remitting multiple sclerosis: a study of thirty patients. *Int Tinnitus J* 2008;**14**:139–45.

56 Yang F, Liu X. Relative importance of vision and proprioception in maintaining standing balance in people with multiple sclerosis. *Mult Scler Relat Dis* 2020;**39**:101901. doi:10.1016/j.msard.2019.101901

57 Doty RL, MacGillivray MR, Talab H, *et al.* Balance in multiple sclerosis: relationship to central brain regions. *Exp Brain Res* 2018;**236**:2739–50. doi:10.1007/s00221-018-5332-1

58 Gabeli T, Krbot M, Šefer AB, *et al.* Ocular and Cervical Vestibular Evoked Myogenic Potentials in Patients With Multiple Sclerosis. *J Clin Neurophysiol* 2013;**30**:86–91. doi:10.1097/wnp.0b013e31827eda0c

59 Cochrane GD, Christy JB, Motl RW. Comprehensive Clinical Assessment of Vestibular Function in Multiple Sclerosis. *J Neurol Phys Ther* 2021;**45**:228–34. doi:10.1097/npt.0000000000000358

60 Elmoazen D, Kozou H, Gawies M, *et al.* Assessment of Cervical and Ocular Vestibular Evoked Myogenic Potentials in Multiple Sclerosis PatientsS. *Egyptian Journal of Ear, Nose, Throat and Allied Sciences* 2021;**22**:1–9. doi:10.21608/ejentas.2020.44108.1262

61 Sartucci F, Logi F. Vestibular-evoked myogenic potentials: A method to assess vestibulo-spinal conduction in multiple sclerosis patients. *Brain Res Bull* 2002;**59**:59–63. doi:10.1016/s0361-9230(02)00842-0

62 Aidar RC, Suzuki FA. Vestibular evoked myogenic potential: new perspectives in multiple sclerosis. *Braz J Otorhinolar* 2005;**71**:48–54. doi:10.1016/s1808-8694(15)31284-2

63 Patkó T, Simó M, Arányi Z. Vestibular click-evoked myogenic potentials: sensitivity and factors determining abnormality in patients with multiple sclerosis. *Mult Scler J* 2007;**13**:193–8. doi:10.1177/1352458506070940

64 Harirchian MH, Karimi N, Nafisi S, *et al.* Vestibular evoked myogenic potential for diagnoses of multiple sclerosis: is it beneficial? *Medicinski Glasnik Official Publ Medical Assoc Zenica-doboj Canton Bosnia Herzegovina* 2013;**10**:321–6.

65 Kavasoğlu G, Gökçay F, Yüceyar N, *et al.* Cervical vestibular-evoked myogenic potentials in patients with multiple sclerosis: sensitive in detecting brainstem involvement? *Neurol Sci* 2018;**39**:365–71. doi:10.1007/s10072-017-3215-z

For peer review only

For peer review only - http://bmjopen.bmj.com/site/about/guidelines.xhtml

66 Koura R, Hussein M. Vestibular-evoked myogenic potential: an easy neurophysiological tool for evaluating brain stem involvement in multiple sclerosis. *Egypt J Otolaryngology* 2018;**34**:144–8. doi:10.4103/ejo.ejo_73_17

67 Cochrane GD, Christy JB, Sandroff BM, *et al.* Cognitive and Central Vestibular Functions Correlate in People With Multiple Sclerosis. *Neurorehab Neural Re* 2021;**35**:1030–8. doi:10.1177/15459683211046268

68 Veuillet E, Khalfa S, Collet L. Clinical relevance of medial efferent auditory pathways. *Scand Audiology Suppl* 1999;**51**:53–62.

69 Protti-Patterson E, Young ML. The use of subjective and objective audiologic test procedures in the diagnosis of multiple sclerosis. *Otolaryng Clin N Am* 1985;**18**:241–55.

70 Magnano I, Aiello I, Piras MR. Cognitive impairment and neurophysiological correlates in MS. *J Neurol Sci* 2006;**245**:117–22. doi:10.1016/j.jns.2005.08.027

71 Musiek FE, Shinn JB, Jirsa R, *et al.* GIN (Gaps-In-Noise) Test Performance in Subjects with Confirmed Central Auditory Nervous System Involvement. *Ear Hearing* 2005;**26**:608–18. doi:10.1097/01.aud.0000188069.80699.41

72 Lord SR, Clark RD, Webster IW. Postural Stability and Associated Physiological Factors in a Population of Aged Persons. *J Gerontology* 1991;**46**:M69–76. doi:10.1093/geronj/46.3.m69

73 Cameron MH, Lord S. Postural Control in Multiple Sclerosis: Implications for Fall Prevention. *Curr Neurol Neurosci* 2010;**10**:407–12. doi:10.1007/s11910-010-0128-0

74 Cameron MH, Horak FB, Herndon RR, *et al.* Imbalance in multiple sclerosis: A result of slowed spinal somatosensory conduction. *Somatosens Mot Res* 2008;**25**:113–22. doi:10.1080/08990220802131127

75 Cattaneo D, Jonsdottir J. Sensory impairments in quiet standing in subjects with multiple sclerosis. *Mult Scler* 2009;**15**:59–67. doi:10.1177/1352458508096874

76 Colebatch JG, Halmagyi GM, Skuse NF. Myogenic potentials generated by a click-evoked vestibulocollic reflex. *J Neurology Neurosurg Psychiatry* 1994;**57**:190–7. doi:10.1136/jnnp.57.2.190

For peer review only

For peer review only - http://bmjopen.bmj.com/site/about/guidelines.xhtml

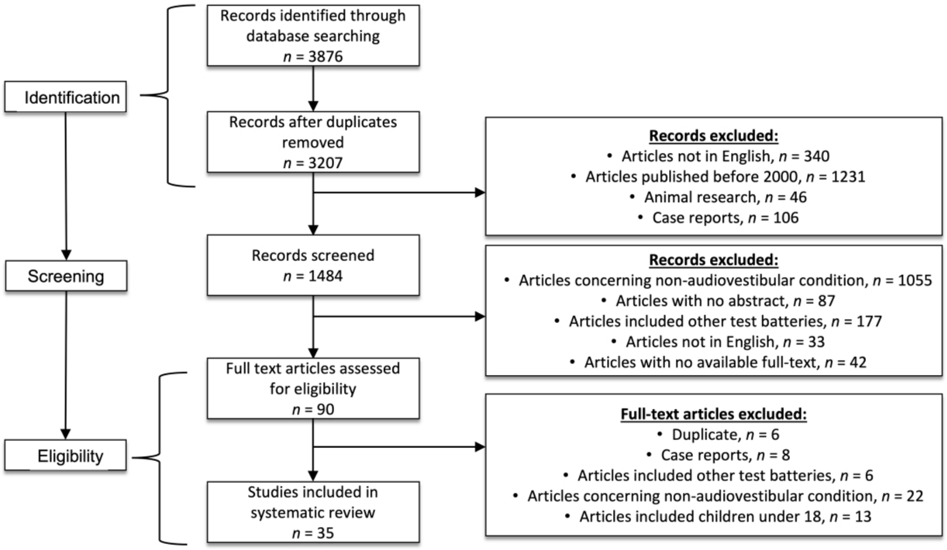

Figure 1. Flow diagram of systematic literature review

Supplement

Section I: Literature Search Strategy

Database searched: **Scopus**

Date of coverage: **2000 to November 2021**

Search undertaken: **August 2020, September 2020, February 2021, November 2021**

((TITLE-ABS-KEY("multiple sclerosis")AND (hearing OR auditory OR "hearing loss" OR "auditory function" OR “hearing dysfunction”)) OR (TITLE-ABS-KEY("multiple sclerosis") AND (dizziness OR vestibular OR vertigo OR equilibrium OR balance OR balanced OR “vestibular function”)

For peer review only

Database searched: Pubmed

Date of coverage: 2000 to November 2021

Search undertaken: August 2020, September 2020, February 2021, November 2021

(("multiple sclerosis"[MeSH Terms] AND ("auditorially"[All Fields] OR "auditory"[All Fields] OR ("hearing"[MeSH Terms] OR "hearing"[All Fields] OR "hearings"[All Fields]) OR "hearing loss"[All Fields] OR "hearing" [All Fields] OR "auditory function"[All Fields]) OR ("multiple sclerosis"[MeSH Terms] AND ("vestibular"[All Fields] OR ("dizziness"[MeSH Terms] OR "dizziness"[All Fields] OR "dizzy"[All Fields] OR "vertigo"[MeSH Terms] OR "vertigo"[All Fields]) OR ("vertigo"[MeSH Terms] OR "vertigo"[All Fields] OR "vertigos"[All Fields] OR "vertigoes"[All Fields]) OR ("equilibrium"[All Fields] OR "equilibriums"[All Fields]) OR ("balance"[All Fields] OR "balanced"[All Fields] OR "balances"[All Fields] OR "balancing"[All Fields]) OR "vestibular function"[All Fields]) AND

Translations

Multiple Sclerosis[MeSH Terms]: "multiple sclerosis"[MeSH Terms]

auditory: "auditorially"[All Fields] OR "auditory"[All Fields]

hearing: "hearing"[MeSH Terms] OR "hearing"[All Fields] OR "hearings"[All Fields]

multiple sclerosis[MeSH Terms]: "multiple sclerosis"[MeSH Terms]

dizziness: "dizziness"[MeSH Terms] OR "dizziness"[All Fields] OR "dizzy"[All Fields] OR "vertigo"[MeSH Terms] OR "vertigo"[All Fields]

vertigo: "vertigo"[MeSH Terms] OR "vertigo"[All Fields] OR "vertigos"[All Fields] OR "vertigoes"[All Fields]

equilibrium: "equilibrium"[All Fields] OR "equilibriums"[All Fields]

balance: "balance"[All Fields] OR "balanced"[All Fields] OR "balances"[All Fields] OR "balancing"[All Fields]

For peer review only - http://bmjopen.bmj.com/site/about/guidelines.xhtml

Database searched: Web of Science

Date of coverage: 2000 to November 2021

Search undertaken: August 2020, September 2020, February 2021, November 2021

Databases= WOS, BCI, BIOSIS, CCC, DRCI, DIIDW, KJD, MEDLINE, RSCI, SCIELO, ZOOREC, Timespan=2000-2021

#1 TS=("multiple sclerosis")

#2 TS=(auditory OR audio OR "auditory function" OR “auditory system”)

#3 TS=(hear* OR hearing OR "hearing loss")

#4 #2 OR #3

#5 #4 AND #1

#6 TS=(vestibular OR vestibul* OR "vestibular dysfunction" OR "vestibular system")

#7 TS=(vertigo)

#8 TS=(dizzy OR dizziness)

#9 TS=(balance OR balanced OR balances OR balanc*)

#10 #6 OR #7 OR #8 OR #9

#11 #1 AND #10

#12 #5 OR #11 AND

For peer review only

Database searched: NICE

Date of coverage: 2000 to November 2021

Search undertaken: August 2020, September 2020, February 2021, November 2021

(("multiple sclerosis") AND (hearing OR auditory OR "hearing loss" OR "auditory function" OR hear* OR “hearing dysfunction)) OR (("multiple sclerosis") AND (dizziness OR vestibular OR vertigo OR equilibrium OR balance OR balanced OR “vestibular function”))

For peer review only

Section II: Assessment of Bias

Table S1: *Assessment of Bias for case-control studies according to RoBANs*

Year	Author	Selection of participants	Confounding Variables	Measurement of Exposure	Blinding of the Outcome of the Assessment	Incomplete Outcome of Data	Selective Outcome Reporting
2002	Japaridze et al.[1]	?	+	+	-	+	+
2002	Sartucci and Logi[2]	+	+	+	-	+	+
2004	Alpini et al.[3]	-	?	+	-	+	+
2005	Aidar and Suzuki[4]	+	+	+	-	+	-
2007	Patkó et al.[5]	?	-	+	-	+	+
2008	Zeigelboim et al.[6]	?	+	+	-	+	+
2009	Lima et al.[7]	?	?	+	-	+	+
2010	Lewis et al.[8]	+	+	+	-	+	+
2010	Matas et al.[9]	+	+	+	-	+	+
2012	Doty et al.[10]	-	+	+	-	+	+
2012	Saberi et al.[11]	+	+	+	-	+	+
2013	Gabelić et al.[12]	+	+	+	-	+	+
2013	Harirchian et al.[13]	+	+	+	-	+	+
2014	Valadbeigi et al.[14]	+	+	+	-	+	+
2015	Pokryszko-Dragan et al.[15]	+	+	+	-	+	+
2018	Doty et al.[16]	-	+	+	-	+	+
2018	Kavasoğlu et al.[17]	+	?	+	-	+	+
2018	Koura et al.[18]	+	+	+	-	+	+
2019	Elbeltagy et al.[19]	-	+	+	-	+	+
2021	Rishiq et al.[20]	?	+	+	-	+	+
2021	Srinivasan et al.[21]	+	+	+	-	+	+
2021	Elmoazen et al.[22]	+	+	+	-	+	+

Green "+" = low risk of bias, red "-" = high risk of bias, yellow "?" = unclear risk of bias.

Table S2: *Risk of bias assessment of cohort studies by the Newcastle-Ottawa Quality Assessment form for cohort studies.*

Year	Author	Representativeness of exposed cohort	Selection of non-exposed cohort	Ascertainment of exposure	Outcome of interest not present at the beginning	Comparability of cohorts/confounders	Assessment of the outcome	Follow-up Length	Adequacy of follow-up of cohorts	Study quality
2002	Versino et al.[23]	*	*n/a*	*	*	*n/a*	-	*n/a*	*	?
2008	Burina et al.[24]	-	*n/a*	*	*n/a*	-	-	-	-	?
2009	Eleftheriadou et al.[25]	*	-	*	*	*	-	*	*	Poor
2010	Degirmenci et al.[26]	*	-	*	*	*	*	*	*	Good
2016	Kaytancı et al.[27]	-	-	*	-	*	-	*	*	Poor
2019	Di Mauro et al.[28]	*	-	*	*	*	-	*	*	Good

'' = low risk of bias, '-' = high risk of bias, "n/a" = the criteria does not apply to the paper, "?" = unknown quality of the paper due to the presence of "n/a" criteria.*

Table S3: *Risk of bias assessment of cross-sectional studies by the Newcastle-Ottawa Quality Assessment form which is adapted for cross-sectional studies.*

Year	Author	Representativeness of the sample	Sample Size	Non-respondents	Ascertainment of exposure (risk factor)	Comparability of groups/confounders	Assessment of the outcome	Statistical test	Study quality
2015	Parsa et al.[29]	-	-	-	*	*	-	*	Poor
2020	Yang and Liu[30]	*	-	*	*	*	-	*	Good
2020	Inojosa et al.[31]	*	-	*	*	*	-	*	Good
2020	Inojosa et al.[32]	*	-	*	*	*	-	*	Good
2021	Delphi et al.[33]	-	-	*	*	*	-	*	Good
2021	Cochrane et al.[34]	*	-	*	*	*	-	*	Good
2021	Cochrane et al.[35]	*	-	*	*	*	-	*	Good

'' = low risk of bias, '-' = high risk of bias.*

Section III: Multiple Sclerosis and Auditory Function

Table S4. Characteristics of studies examining the effects of MS on auditory function

Test Battery	Author (Year)	Study Type	Sample Size: PwMS	Sample Size: Controls	PwMS Mean Age (years)	PwMS Age range (Years)	% Female	MS Type (sample size)	MS Duration Mean (range) years	Results of PwMS compared to controls / normative data
ABR	Japaridze et al. (2002)[1]	Case-Control	40	33	30.5	18-57	77.5	?	?	65% Abnormal
	Versino et al. (2002)[23]	Cohort	65	18	35.5	19-61	?	?	?	37.5% Abnormal
	Burina et al. (2008)[24]	Cohort	60	NA	37.2	?	68.3	RRMS	?	95% Abnormal
	Eleftheriadou et al. (2009)[25]	Cohort	46	40	40	20-66	45.6	RRMS	4.6	26% Abnormal
	Lima et al. (2009)[7]	Case-control	25	NA	42.6 (female)<br>38 (male)	33-53 (female)<br>24-56 (male)	64.0	RRMS (14), PPMS (8), Unspecified Progressive (3)	?	30% Abnormal
	Matas et al. (2010)[9]	Case-control	25	25	34.88	25-55	76.0	RRMS	4.25 (?)	Significantly different
	Saberi et al. (2012)[11]	Case-control	60	38	29.9	?	73.3	?	3.2 (?)	20% Abnormal
	Pokryszko-Dragan et al. (2015)[15]	Case-control	86	40	39.55	19-60	72.0	?	8.57 (1-30)	Significantly different
	Kaytancı et al. (2016)[27]	Cohort	20	20	31.3	?	55.0	?	?	Significantly different
	Di Mauro et al. (2019)[28]	Cohort	40	40	37	18-50	60.0	RRMS	0.8 (0.8-2)	No difference
	Delphi et al. (2021)[33]	Cross-sectional	25	25	31.43	18-45	72.0	?	?	Significantly different
	Rishiq et al. (2021)[20]	Case-control	11	9	49.5	34-68	72.7	RRMS (9), PPMS (1), SPMS (1)	11.9 (2-30)	Significantly different at high click rate
	Srinivasan et al. (2021)[21]	Case-control	45	45	31.77	18-50	73.3	RRMS	6.1 (5.37)	Significantly different and 55.56 % abnormal
PTA	Lewis et al. (2010)[8]	Case-control	47	49	51.4	21-65	57.7 RRMS, 28.6 SPMS	RRMS (26), SPMS (21)	RRMS: 12.6 (2-43)<br>SPMS: 23.3 (8-50)	Worse thresholds
	Doty et al. (2012)[10]	Case-control	73	73	Males: 45.24<br>Females: 45.6	?	71.2	RRMS (57), PPMS (3), SPMS (6), Unspecified (7)	Males: 7.36 (?)<br>Females: 7.84 (?)	No significant difference
	Saberi et al. (2012)[11]	Case-control	60	38	29.9	?	73.3	?	3.2 (?)	Worse thresholds in female PwMS
	Di Mauro et al. (2019)[28]	Cohort	40	40	37	18-50	60.0	RRMS	0.8 (0.8-2)	No significant difference
	Srinivasan et al. (2021)[21]	Case-control	45	45	31.77	18-50	73.3	RRMS	6.1 (5.37)	Significantly different
TEOAEs & DPOAEs	Saberi et al. (2012)[11]	Case-control	60	38	29.9	?	73.3	?	3.2 (?)	No significant difference
	Kaytancı et al. (2016)[27]	Cohort	20	20	31.3	?	55.0	?	?	No significant difference
	Di Mauro et al. (2019)[28]	Cohort	40	40	37	18-50	60.0	RRMS	0.8 (0.8-2)	Significantly lower

Table S4 continued: Characteristics of studies examining the effects of MS on auditory function

Test Battery	Author (Year)	Study Type	Sample Size PwMS	Sample Size Controls	PwMS Mean Age (years)	PwMS Age range (Years)	% Female	MS Type (sample size)	MS Duration Mean (range) years	Results of PwMS compared to controls / normative data
WDS	Lewis et al. (2010)[8]	Case-control	47	49	51.4	21-65	57.7 RRMS, 28.6 SPMS	RRMS (26), SPMS (21)	RRMS: 12.6 (2-43) SPMS: 23.3 (8-50)	No significant difference
	Valadbeigi et al. (2014)[[14]	Case-control	26	26	28.9	18-40	?	RRMS	?	Lower scores PwMS
	Elbeltagy et al. (2019)[19]	Case-control	20	20	37.6	30-50	?	RRMS	?	Significantly lower
DPST	Valadbeigi et al. (2014)[14]	Case-control	26	26	28.9	18-40	?	RRMS	?	Significantly different
GIN	Valadbeigi et al. (2014)[14]	Case-control	26	26	28.9	18-40	?	RRMS	?	Significantly different
	Elbeltagy et al. (2019)[19]	Case-control	20	20	37.6	30-50	?	RRMS	?	Significantly different
CAEP	Japaridze et al. (2002)[1]	Case-Control	40	33	30.5	18-57	77.5	?	?	30% Abnormal
	Matas et al. (2010)[9]	Case-control	25	25	34.88	25-55	76.0	RRMS	4.25 (?)	16% Abnormal

Abbreviations: PTA= Pure Tone Audiometry, ABR= Auditory Brainstem Response, OAE= Oto-acoustic Emissions, TEOAE= Transient Evoked Oto acoustic emission, DPOAE= Distortion Product Oto acoustic Emissions, CAEP= Cortical Auditory Evoked Potentials, SCP= Slow Cortical Potentials, GIN= Gaps in Noise test, WDS= Word Discrimination Scores, DPST= Duration pattern sequence test, MS= Multiple Sclerosis, PwMS = Persons with Multiple Sclerosis, ? = not reported.
Cells shaded in grey represent test results that were abnormal or showed a difference between PwMS and Controls

For peer review only - http://bmjopen.bmj.com/site/about/guidelines.xhtml

Extracted Pure Tone Audiometry Thresholds and Auditory Brainstem Response Results

Table S5: Pure Tone Audiometry Thresholds

Author (Year)	Group	Test Ear	PTA Threshold Per Frequency (Hz)													
			125		250		500		1000		2000		4000		8000	
			$\bar{x}$	SD	$\bar{x}$	SD	$\bar{x}$	SD	$\bar{x}$	SD	$\bar{x}$	SD	$\bar{x}$	SD	$\bar{x}$	SD
Doty et al. (2012)[10]	Male PwMS	Average Across Ears	Not Tested		15.87	5.39	14.05	5.94	12.52	7.43	12.79	8.97	20.03	11.71	17.02	17.26
	Male Controls				14.35	8.24	15.90	8.68	15.53	8.58	17.74	8.93	22.77	16.08	20.23	22.49
	Female PwMS				12.85	6.88	12.49	6.50	12.05	6.59	14.62	7.19	15.65	8.97	13.28	12.38
	Female Controls				11.28	7.77	12.52	7.60	10.45	6.82	11.62	8.17	13.92	8.79	12.65	12.60
Di Mauro et al. (2019)[28]	PwMS	Right	10.30	1.60	10.10	0.08	10.10	0.08	10.40	1.10	11.10	1.70	10.80	1.80	13.00	2.60
	Controls	Right	10.90	1.40	10.80	1.60	11.00	1.90	10.80	2.30	11.00	1.20	11.90	2.40	13.90	2.40
	PwMS	Left	10.30	1.70	10.90	1.20	10.40	1.90	10.40	1.30	10.80	1.10	11.40	1.70	12.50	2.20
	Controls	Left	10.90	1.40	10.80	1.60	11.00	1.30	10.90	2.10	11.10	1.90	11.90	2.80	13.90	2.40
Srinivasan et al. (2021)[21]	PwMS	Average Across Ears	Not Tested		12.22	6.40	13.11	5.92	13.61	5.90	12.55	7.19	13.66	8.06	16.16	9.08
	Controls				8.94	5.05	9.50	4.17	9.88	3.82	9.50	5.45	10.88	5.83	12.05	5.89

Abbreviations:
PwMS = Persons with Multiple Sclerosis, PTA = Pure Tone Audiometry, $\bar{x}$ = Mean, SD = Standard Deviation

For peer review only

Table S6: Auditory Brainstem Response Parameters and Results

Author (Year)	ABR Parameters							Peak Latency (ms)						Interpeak Latencies (ms)						Peak Amplitude (µV)			
	Click Polarity - Transducer	PR	PL	Ave.	BP Filter	Group	Test Ear	I		III		V		I-III		III-V		I-V		I		V	
								x̄	SD	x̄	SD	x̄	SD	x̄	SD	x̄	SD	x̄	SD	x̄	SD	x̄	SD
Lima et al. (2009)[7]	Rarefaction - TDH 39 Headphone	13.10	60 dB SL*	1024	100-3000	PwMS	Right	1.50	0.16	3.63	0.30	5.56	0.40	2.13	0.22	1.93	0.31	4.06	0.33	?	?	?	?
							Left	1.53	0.20	3.63	0.30	5.64	0.43	2.10	0.31	2.01	0.29	4.11	0.39	?	?	?	?
Matas et al. (2010)[9]	Rarefaction - TDH 39 Headphone	19.90	80 dB nHL	2000	100-3000	PwMS	Average Across Ears	1.53	0.13	3.72	0.31	5.78	0.37	2.21	0.26	2.03	0.27	4.26	0.35	?	?	?	?
						Controls		1.53	0.07	3.57	0.09	5.50	0.45	2.04	0.06	1.98	0.08	4.03	0.06	?	?	?	?
Pokryszko-Dragan et al. (2015)[15]	? - Earphone unspecified	20.30	65 dB SL*	200	150-3000	PwMS	Right	1.72	0.19	3.88	0.33	5.81	0.81	2.18	0.19	2.00	0.30	4.19	0.39	0.29	0.18	0.37	0.20
						Controls		1.70	0.14	3.85	0.16	5.73	0.23	2.14	0.12	1.87	0.18	4.02	0.20	0.30	0.10	0.46	0.19
						PwMS	Left	1.68	0.12	3.87	0.17	5.77	0.73	2.19	0.16	1.96	0.30	4.15	0.34	0.32	0.14	0.40	0.24
						Controls		1.69	0.13	3.83	0.12	5.70	0.21	2.13	0.14	1.87	0.17	4.00	0.19	0.30	0.14	0.44	0.16
Kaytancı et al. (2016)[27]	? - TDH 39 Headphone	13.00	70 dB nHL	1500	2000-4000	PwMS	Right	2.03	0.27	4.36	0.49	6.26	0.35	2.33	0.49	1.90	0.28	4.23	0.42	?	?	?	?
						Controls		1.45	0.30	3.52	0.31	5.38	0.53	2.07	0.44	1.85	0.48	3.92	0.53	?	?	?	?
						PwMS	Left	1.86	0.24	4.41	0.39	6.25	0.43	2.54	0.34	1.84	0.20	4.38	0.43	?	?	?	?
						Controls		1.47	0.24	3.40	0.43	5.39	0.46	1.92	0.48	1.99	0.53	3.91	0.47	?	?	?	?
						PwMS	IA Diff.	?	?	?	?	0.40	0.46	0.35	0.24	0.49	0.46	0.40	0.48	?	?	?	?
						Controls		?	?	?	?	0.15	0.12	0.34	0.35	0.27	0.30	0.19	0.16	?	?	?	?
Delphi et al. (2021)[33]	Rarefaction - ?	9.00	70 dB nHL	?	30-3000	PwMS	Right	1.89	0.11	4.25	0.14	6.32	0.13	2.30	0.11	2.21	0.09	4.48	0.13	?	?	?	?
						Controls		1.87	0.14	4.01	0.13	5.79	0.14	2.17	0.09	1.99	0.13	4.12	0.11	?	?	?	?
						PwMS	Left	1.80	0.15	4.30	0.15	6.19	0.14	2.29	0.08	2.19	0.14	4.51	0.11	?	?	?	?
						Controls		1.79	0.11	3.96	0.13	5.87	0.15	2.15	0.14	1.96	0.08	4.09	0.14	?	?	?	?
Srinivasan et al. (2021)[21]	Rarefaction – Insert earphones	21.10	80 dB nHL	1500	100-3000	PwMS	Average Across Ears	1.44	0.18	3.61	0.40	5.63	0.73	2.17	0.38	2.03	0.50	4.19	0.70	?	?	?	?
						Controls		1.43	0.16	3.48	0.19	5.30	0.24	2.04	0.17	1.82	0.18	3.86	0.21	?	?	?	?
		80.10				PwMS	Average Across Ears	?	?	?	?	6.12	0.72	?	?	?	?	?	?	?	?	?	?
						Controls		?	?	?	?	5.76	0.27	?	?	?	?	?	?	?	?	?	?

Abbreviations:
PR = Presentation Rate, PL = Presentation Level, Ave. = Number of Averages, BP = Band Pass, , x̄ = Mean, SD = Standard Deviation, dB SL = Decibels Sensation Level, dB nHL = Decibels normal Hearing Level, PwMS = Persons with Multiple Sclerosis, IA Diff. = Inter-aural Difference, ? = not reported.
* dB SL reference to hearing thresholds (did not specify hearing thresholds to click or to pure tones)

Section IV: Multiple Sclerosis and Vestibular Function

Table S7. Characteristics of studies examining the effects of MS on vestibular function

Test Battery	*Author (Year)*	*Study Type*	*Sample Size*		*Mean Age PwMS (years)*	*PwMS Age range (Years)*	*% Female*	*MS Type (sample size)*	*MS Duration Mean (range) years*	*Results of PwMS compared to controls / normative data*
			PwMS	*Controls*						
cVEMP	Sartucci & Logi (2002)[2]	Case-Control	15	15	44.5	26-59	66.6	?	?	Significantly different
	Versino et al. (2002)[23]	Cohort	70	18	35.5	19-61	?	?	?	31.4% Abnormal
	Alpini et al. (2004)[3]	Case-control	40	25	38	17-71	57.5	?	?	70% Abnormal
	Aidar and Suzuki (2005)[4]	Case-control	15	15	39.3	?	?	?	?	Significantly different
	Patkó et al. (2007)[5]	Case-control	30	30	43.4	27-60	66.6	?	?	Significantly different
	Eleftheriadou et al. (2009)[25]	Cohort	46	40	40	20-66	45.6	RRMS	4.6 (?)	Significantly different
	Gabelić et al. (2013)[12]	Case control	30	15	?	?	46.6	RRMS	3.93 (0.2-21)	Significantly different
	Harirchian et al. (2013)[13]	Case-control	20	20	30	20-40	50	RRMS<br>SPMS	?	70% Abnormal
	Parsa et al. (2015)[29]	Cross-sectional	34	15	29.8	?	100	?	?	Significantly different
	Kavasoğlu et al. (2018)[17]	Case-control	30	31	30	18-45	60.0	?	< 1 (?)	23.3% Abnormal<br>No significant difference
	Koura and Hussein (2018)[18]	Case-control	20	10	36.80	?	65.0	?	4.4 (?)	100% Abnormal
	Delphi et al. (2021)[33]	Cross-sectional	25	25	31.43	18-45	72.0	?	?	Significantly different
	Elmoazen et al. (2021)[22]	Case-control	20	10	Brainstem lesions: 40<br>No Brainstem lesion: 34.1	?	?	?	?	Significantly different
	Cochrane et al. (2021)[34,35]	Cross-sectional	40	20	42.4	21-55	88.0	RRMS	9.9	No significant difference
oVEMP	Gabelić et al. (2013)[12]	Case control	30	15	?	?	46.6	RRMS	3.93 (0.2-21)	Significantly different
	Parsa et al. (2015)[29]	Cross-sectional	34	15	29.8	?	100	?	?	Significantly different
	Elmoazen et al. (2021)[22]	Case-control	20	10	Brainstem lesions: 40<br>No Brainstem lesion: 34.1	?	?	?	?	Significantly different
	Cochrane et al. (2021)[34,35]	Cross-sectional	40	20	42.4	21-55	88.0	RRMS	9.9	No significant difference
ENG	Zeigelboim et al. (2008)[6]	Case-control	30	0	42.23	27-64	80	RRMS	?	86.7% Abnormal
	Degirmenci et al. (2010)[26]	Cohort	30	30	37.9	23-56	56.7	RRMS	?	90% Abnormal

For peer review only

Table S7 continued: Characteristics of studies examining the effects of MS on vestibular function

Test Battery	*Author (Year)*	*Study Type*	*Sample Size*		*Mean Age PwMS (years)*	*PwMS Age range (Years)*	*% Female*	*MS Type (sample size)*	*MS Duration Mean (range) years*	*Results of PwMS compared to controls / normative data*
			PwMS	*Controls*						
**Dynamic Posturography**	Doty et al. (2018)[16]	Case-control	58	72	Males:44.61<br>Females: 44.60	?	68.9	?	Male: 7.03 (?)<br>Female: 6.54 (?)	Significantly different
**Static Posturography**	Inojosa et al. (2020)[31,32]	Cross-sectional	99	30	35.01	18-50	68.7	?	5.5 (?)	Significantly different
	Yang and Liu (2020)[30]	Cross-sectional	30	25	50.8	?	76.7	?	14 (?)	Significantly different
**Rotary Chair**	Cochrane et al. (2021)[34,35]	Cross-sectional	40	20	42.4	21-55	88.0	RRMS	9.9	Significantly different

Abbreviations: cVEMP = Cervical Vestibular Evoked Myogenic Potentials, oVEMP = Ocular Vestibular Evoked Myogenic Potentials, MS= Multiple Sclerosis, PwMS = Persons with Multiple Sclerosis, ? = not reported.
Cells shaded in grey represent test results that were abnormal or showed a difference between PwMS and Controls.

For peer review only

Extracted Vestibular Evoked Myogenic Potentials Results

Table S8: Ocular Vestibular Evoked Myogenic Potentials Parameters and Results

Author (Year)	Stimulus (polarity) - Trasducer	PR (Hz)	PL	Ave.	BP Filter (Hz)	Group	Side	Peak Latency (ms) n1 x̄	n1 SD	p1 x̄	p1 SD	Amplitude (µV) n1-p1 x̄	n1-p1 SD
Gabelić et al. (2013)[12]	Click (?) - Headphone	1	130 dB SPL	100	5-1000	PwMS	Average Across sides	10.30	?	14.90	?	13.10	?
						Controls		9.5	?	14	?	13.3	?
Parsa et al. (2015)[29]	500 Hz Tone Burst (?) - Insert earphones	?	95 db nHL	?	?	PwMS with infratentorial plaque	Right	15.67	1.32	20.59	1.65	6.61	0.88
						PwMS without infratentorial plaque		12.21	1.00	16.90	1.32	6.61	0.60
						Controls		10.2	0.48	15.1	0.87	12.77	4.12
						PwMS with infratentorial plaque	Left	15.74	1.66	21.40	2.88	6.48	0.68
						PwMS without infratentorial plaque		11.78	0.89	16.92	0.82	6.82	0.75
						Controls		10.3	0.51	14.9	0.84	12.6	4.04
Elmoazen et al. (2021)[22]	500 Hz Tone Burst (condensation) -Supra aural headphones	5	95 dB nHL	150	1-1000	PwMS with Brainstem lesions	Average Across sides	11.43	1.42	16.86	1.79	3.59	3.82
						PwMS without Brainstem lesions		10.58	2.50	15.81	1.42	3.22	2.00
						Controls		10.09	0.92	14.71	1.20	2.76	1.28
Cochrane et al. (2021)[34]	Unspecified Tone Burst (?) – earphone unspecified	?	?	100	?	PwMS	?	8.50	1.70	12.90	2.20	5.20	3.50
						Controls		8.40	1.50	12.30	2.10	4.70	2.50

Abbreviations:
PR = Presentation Rate, PL = Presentation Level, Ave. = Number of Averages, BP = Band Pass, , x̄ = Mean, SD = Standard Deviation, dB SPL = Decibels Sound Pressure Level, dB nHL = Decibels normal Hearing Level, PwMS = Persons with Multiple Sclerosis, ? = not reported.

Table S9: Cervical Vestibular Evoked Myogenic Potentials Parameters and Results

Author (Year)	Stimulus (polarity) - Trasducer	PR (Hz)	PL	Ave.	BP Filter (Hz)	Group	Side	Peak Latency (ms) p13 x̄	p13 SD	n23 x̄	n23 SD	Amplitude (µV) p13-n23 x̄	p13-n23 SD
Sartucci and Logi (2002)[2]	Click (Rarefaction) - Headphone	3	140 dB SPL	256	10-1500	PwMS	Right	15.30	3.10	22.50	2.30	63.00	65.50
						Controls		12.5	0.8	21.3	1.5	186.4	82.8
						PwMS	Left	14.00	2.30	20.80	2.30	73.10	58.80
						Controls		12.3	0.9	20.8	1.3	141.1	85.8
Alpini et al. (2004)[3]	Click (Rarefaction) - Headphone	5	95 dB nHL + 70 dB SPL contralateral Masking	400	?	PwMS	Right	14.40	?	22.40	?	?	?
							Left	14.90	?	22.50	?	?	?
Aidar and Suzuki (2005)[4]	Click (Rarefaction) - Headphone	2	95 dB HL	200	20-1000	PwMS	Right	13.40	2.87	18.64	2 .67	12 .17	6 .50
						Controls		11 .12	1.64	15.15	2.51	7.54	5.95
						PwMS	Left	13.49	1 .77	17.84	1.27	13.30	3 .63
						Controls		10.76	1.59	14.86	2.85	7.23	4.76
Patkó et al. (2007)[5]	Click (Rarefaction) - Headphone	5	133 dB SPL	250	10-1600	PwMS	Right	14.40	1.50	21.30	3.20	11.61	3.63
						Controls		12.7	1.5	21.3	3.7	16.02	5.34
						PwMS	Left	14.8	1.5	21.2	3.2	11.07	2.87
						Controls		12.9	1.9	20.7	2.8	13.85	4.3
Eleftheriadou et al. (2009)[25]	Click (Rarefaction) - Headphone	5	105 dB HL	250	5-1500	PwMS with brainstem leasions	Average acrross sides	15.48	1.40	23.94	1.51	?	?
						PwMS without brainstem leasions		14.56	1.20	23.25	1.42	?	?
						Controls		12.67	1.1	21.66	1.11	?	?
Gabelić et al. (2013)[12]	Click (?) - Headphone	1	130 dB SPL	100	5-1000	PwMS	Average across sides	15.80	?	24.90	?	1.90	?
						Controls		14.6	?	22.1	?	1.6	?
Harirchian et al. (2013)[13]	Click (Rarefaction) - Headphone	2	95 db nHL + 40 dB nHL contralateral Masking	200	10-1000	PwMS	Right	22.20	9.30	30.02	11.20	?	?
						Controls		12.9	1.4	21.3	1.6	?	?
						PwMS	Left	20.20	7.05	30.71	16.20	?	?
						Controls		13.1	1.9	20.6	1.9	?	?
						PwMS	IA Diff.	2.05	8.80	0.70	10.90	?	?
						Controls		0.17	2.2	0.4	2.5	?	?

Table S9 Continued: Cervical Vestibular Evoked Myogenic Potentials Parameters and Results

Author (Year)	Stimulus (polarity) - Trasducer	PR (Hz)	PL	Ave.	BP Filter (Hz)	Group	Side	Peak Latency (ms)				Amplitude (µV)	
								p13		n23		p13-n23	
								x̄	SD	x̄	SD	x̄	SD
Parsa et al. (2015)[29]	500 Hz Tone Burst (?) -Insert earphones	?	95 db nHL	?	?	PwMS with infratentorial plaque	Right	18.75	1.81	28.15	1.43	118.82	29.97
						PwMS without infratentorial plaque		17.44	1.03	26.89	1.87	135.00	20.31
						Controls		15.25	0.9	24.55	1.07	180	44.97
						PwMS with infratentorial plaque	Left	19.11	1.72	28.88	2.27	119.65	26.02
						PwMS without infratentorial plaque		17.21	1.05	26.87	1.36	133.78	22.89
						Controls		15.31	0.93	24.48	1.18	175	57.59
Kavasoğlu et al. (2017)[17]	Click (Rarefaction) - Headphone	5	100 dB nHL	100	10-1000	PwMS	Right	12.90	2.10	21.20	1.78	7.10	2.80
						Controls		12	0.9	20.6	1.95	7.7	3.1
						PwMS	Left	12.90	2.20	21.40	2.10	7.00	2.50
						Controls		12.03	0.96	20.76	1.63	7.6	3
Koura and Hussein (2018)[18]	500 Hz Tone Burst (Rarefaction) -TDH 39 Headphones	5.1	95 db HL	200	?	PwMS	Right	17.14	0.71	26.82	0.84	41.88	1. 71
						Controls		13.83	1.14 5	20.76	3.43	61.18	34.64
						PwMS	Left	17.20	0.56	27.17	0.75	54.95	28.27
						Controls		14.43	0.70 2	22.3	1.725	65.74	18.51
Delphi et al. (2021)[33]	500 Hz Tone Burst (?) -?	5.1	95 dB nHL	?	10-1200	PwMS	Right	18.20	1.93	27.37	2.45	121.34	29.05
						Controls		14.96	1.02	24.21	2.10	169.21	48.76
						PwMS	Left	18.95	2.01	27.21	2.80	-	-
						Controls		15.02	1.10	24.12	2.30	-	-
Elmoazen et al. (2021)[22]	500 Hz Tone Burst (condensation) -Supra aural headphones	5	95 dB nHL	150	1-1000	PwMS with Brainstem lesions	Average across sides	16.49	1.49	25.96	1.93	66.25	52.42
						PwMS without Brainstem lesions		15.21	1.57	23.97	2.06	93.12	78.01
						Controls		14.05	0.94	23.36	2.14	144.42	122.73
Cochrane et al. (2021)[34]	Unspecified Tone Burst (?) – earphone unspecified	?	?	?	?	PwMS	?	13.70	1.30	21.10	2.60	12.70	7.60
						Controls		13.30	0.50	20.60	1.80	16.50	7.10

Abbreviations:
PR = Presentation Rate, PL = Presentation Level, Ave. = Number of Averages, BP = Band Pass, , x̄ = Mean, SD = Standard Deviation, dB SPL = Decibels Sound Pressure Level, dB HL = Decibels Heraing Level, dB nHL = Decibels normal Hearing Level, PwMS = Persons with Multiple Sclerosis, IA Diff. = Inter-aural Difference, ? = not reported. Cells Highlighted in Yellow indicate that "normalized amplitudes" were reported as opposed to absolute amplitudes reported by other studies. Cells Highlighted in Green indicate results were reported without specifying side or if they are averaged across sides.

For peer review only - http://bmjopen.bmj.com/site/about/guidelines.xhtml

1 Japaridze G, Shakarishvili R, Kevanishvili Z. Auditory brainstem, middle-latency, and slow cortical responses in multiple sclerosis. *Acta Neurol Scand* 2002;**106**:47–53. doi:10.1034/j.1600-0404.2002.01226.x

2 Sartucci F, Logi F. Vestibular-evoked myogenic potentials: A method to assess vestibulo-spinal conduction in multiple sclerosis patients. *Brain Res Bull* 2002;**59**:59–63. doi:10.1016/s0361-9230(02)00842-0

3 Alpini D, Pugnetti L, Caputo D, *et al.* Vestibular evoked myogenic potentials in multiple sclerosis: clinical and imaging correlations. *Mult Scler* 2004;**10**:316–21. doi:10.1191/1352458504ms1041oa

4 Aidar RC, Suzuki FA. Vestibular evoked myogenic potential: new perspectives in multiple sclerosis. *Braz J Otorhinolar* 2005;**71**:48–54. doi:10.1016/s1808-8694(15)31284-2

5 Patkó T, Simó M, Arányi Z. Vestibular click-evoked myogenic potentials: sensitivity and factors determining abnormality in patients with multiple sclerosis. *Mult Scler J* 2007;**13**:193–8. doi:10.1177/1352458506070940

6 Zeigelboim BS, Arruda WO, Mangabeira-Albernaz PL, *et al.* Vestibular findings in relapsing, remitting multiple sclerosis: a study of thirty patients. *Int Tinnitus J* 2008;**14**:139–45.

7 Lima TMA, Crato AN, Mancini PC, *et al.* Alterations in early auditory evoked potentials in multiple sclerosis patients. *Braz J Otorhinolar* 2009;**75**:177–81. doi:10.1016/s1808-8694(15)30775-8

8 Lewis MS, Lilly DJ, Hutter MM, *et al.* Audiometric hearing status of individuals with and without multiple sclerosis. *J Rehabilitation Res Dev* 2010;**47**:669–78. doi:10.1682/jrrd.2010.01.0010

9 Matas CG, Matas SL de A, Oliveira CRS de, *et al.* Auditory evoked potentials and multiple sclerosis. *Arq Neuro-psiquiat* 2010;**68**:528–34. doi:10.1590/s0004-282x2010000400010

10 Doty RL, Tourbier I, Davis S, *et al.* Pure-Tone Auditory Thresholds Are Not Chronically Elevated in Multiple Sclerosis. *Behav Neurosci* 2012;**126**:314–24. doi:10.1037/a0027046

11 Saberi A, Hatamian HR, Nemati S, *et al.* Hearing statement in multiple sclerosis: a case control study using auditory brainstem responses and otoacoustic emissions. *Acta Medica Iranica* 2012;**50**:679–83.

12 Gabeli T, Krbot M, Šefer AB, *et al.* Ocular and Cervical Vestibular Evoked Myogenic Potentials in Patients With Multiple Sclerosis. *J Clin Neurophysiol* 2013;**30**:86–91. doi:10.1097/wnp.0b013e31827eda0c

13 Harirchian MH, Karimi N, Nafisi S, *et al.* Vestibular evoked myogenic potential for diagnoses of multiple sclerosis: is it beneficial? *Medicinski Glasnik Official Publ Medical Assoc Zenica-doboj Canton Bosnia Herzegovina* 2013;**10**:321–6.

For peer review only - http://bmjopen.bmj.com/site/about/guidelines.xhtml

14 Valadbeigi A, Weisi F, Rohbakhsh N, *et al.* Central auditory processing and word discrimination in patients with multiple sclerosis. *Eur Arch Oto-rhino-l* 2014;**271**:2891–6. doi:10.1007/s00405-013-2776-6

15 Pokryszko-Dragan A, Bilinska M, Gruszka E, *et al.* Assessment of visual and auditory evoked potentials in multiple sclerosis patients with and without fatigue. *Neurol Sci* 2015;**36**:235–42. doi:10.1007/s10072-014-1953-8

16 Doty RL, MacGillivray MR, Talab H, *et al.* Balance in multiple sclerosis: relationship to central brain regions. *Exp Brain Res* 2018;**236**:2739–50. doi:10.1007/s00221-018-5332-1

17 Kavasoğlu G, Gökçay F, Yüceyar N, *et al.* Cervical vestibular-evoked myogenic potentials in patients with multiple sclerosis: sensitive in detecting brainstem involvement? *Neurol Sci* 2018;**39**:365–71. doi:10.1007/s10072-017-3215-z

18 Koura R, Hussein M. Vestibular-evoked myogenic potential: an easy neurophysiological tool for evaluating brain stem involvement in multiple sclerosis. *Egypt J Otolaryngology* 2018;**34**:144–8. doi:10.4103/ejo.ejo_73_17

19 Elbeltagy R, Gad N, Ismail M. Central auditory function in multiple sclerosis patients. *Indian J Otology* 2019;**25**:90–6. doi:10.4103/indianjotol.indianjotol_80_18

20 Rishiq D, Harkrider A, Springer C, *et al.* Click-evoked and speech-evoked auditory brainstem responses from individuals with multiple sclerosis. *Neurosci Lett* 2021;**740**:135460. doi:10.1016/j.neulet.2020.135460

21 Srinivasan VS, Krishna R, Munirathinam BR. Effectiveness of Brainstem Auditory Evoked Potentials Scoring in Evaluating Brainstem Dysfunction and Disability Among Individuals With Multiple Sclerosis. *Am J Audiol* 2021;**30**:255–65. doi:10.1044/2020_aja-20-00155

22 Elmoazen D, Kozou H, Gawies M, *et al.* Assessment of Cervical and Ocular Vestibular Evoked Myogenic Potentials in Multiple Sclerosis PatientsS. *Egyptian Journal of Ear, Nose, Throat and Allied Sciences* 2021;**22**:1–9. doi:10.21608/ejentas.2020.44108.1262

23 Versino M, Colnaghi S, Callieco R, *et al.* Vestibular evoked myogenic potentials in multiple sclerosis patients. *Clin Neurophysiol* 2002;**113**:1464–9. doi:10.1016/s1388-2457(02)00155-4

24 Burina A, Sinanović O, Smajlović D, *et al.* Some Aspects of Balance Disorder in Patients with Multiple Sclerosis. *Bosnian J Basic Med* 2008;**8**:80–5. doi:10.17305/bjbms.2008.3003

25 Eleftheriadou A, Deftereos SN, Zarikas V, *et al.* The diagnostic value of earlier and later components of Vestibular Evoked Myogenic Potentials (VEMP) in Multiple Sclerosis. *J Vestib Res* 2009;**19**:59–66. doi:10.3233/ves-2009-0342

26 Degirmenci E, Bir LS, Ardic FN. Clinical and electronystagmographical evaluation of vestibular symptoms in relapsing remitting multiple sclerosis. *Neurol Res* 2010;**32**:986–91. doi:10.1179/016164110x12681290831405

For peer review only - http://bmjopen.bmj.com/site/about/guidelines.xhtml

27 Kaytancı E, Ozdamar OI, Acar GO, *et al.* Evaluation of transiently evoked otoacoustic emissions and auditory brainstem responses in patients with multiple sclerosis. *Ear Nose Throat J* 2016;**95**:E12–7.

28 Di Mauro RD, Di Girolamo SD, Ralli M, *et al.* Subclinical cochlear dysfunction in newly diagnosed relapsing-remitting multiple sclerosis. *Mult Scler Relat Dis* 2019;**33**:55–60. doi:10.1016/j.msard.2019.05.020

29 Parsa MS, Mohammadkhani G, Hajabolhassani F, *et al.* Cervical and ocular vestibular evoked myogenic potentials in multiple sclerosis participants. *Medical J Islamic Repub Iran* 2015;**29**:164.

30 Yang F, Liu X. Relative importance of vision and proprioception in maintaining standing balance in people with multiple sclerosis. *Mult Scler Relat Dis* 2020;**39**:101901. doi:10.1016/j.msard.2019.101901

31 Inojosa H, Schriefer D, Klöditz A, *et al.* Balance Testing in Multiple Sclerosis—Improving Neurological Assessment With Static Posturography? *Front Neurol* 2020;**11**:135. doi:10.3389/fneur.2020.00135

32 Inojosa H, Schriefer D, Trentzsch K, *et al.* Visual Feedback and Postural Control in Multiple Sclerosis. *J Clin Medicine* 2020;**9**:1291. doi:10.3390/jcm9051291

33 Delphi M, Sayaf M, Taheri F, *et al.* Audio and vestibular analyses in patient with multiple sclerosis: a case–control study. *Hear Balance Commun* 2021;**19**:1–6. doi:10.1080/21695717.2021.1933318

34 Cochrane GD, Christy JB, Motl RW. Comprehensive Clinical Assessment of Vestibular Function in Multiple Sclerosis. *J Neurol Phys Ther* 2021;**45**:228–34. doi:10.1097/npt.0000000000000358

35 Cochrane GD, Christy JB, Sandroff BM, *et al.* Cognitive and Central Vestibular Functions Correlate in People With Multiple Sclerosis. *Neurorehab Neural Re* 2021;**35**:1030–8. doi:10.1177/15459683211046268

For peer review only

PRISMA 2020 Checklist

Section and Topic	Item #	Checklist item	Location where item is reported
**TITLE**			
Title	1	Identify the report as a systematic review.	**Page 1 & 4**
**ABSTRACT**			
Abstract	2	See the PRISMA 2020 for Abstracts checklist.	**Page 3 – 4**
**INTRODUCTION**			
Rationale	3	Describe the rationale for the review in the context of existing knowledge.	**Page 4 – 6**
Objectives	4	Provide an explicit statement of the objective(s) or question(s) the review addresses.	**Page 6**
**METHODS**			
Eligibility criteria	5	Specify the inclusion and exclusion criteria for the review and how studies were grouped for the syntheses.	**Page 7 – 9**
Information sources	6	Specify all databases, registers, websites, organisations, reference lists and other sources searched or consulted to identify studies. Specify the date when each source was last searched or consulted.	**Page 6**
Search strategy	7	Present the full search strategies for all databases, registers and websites, including any filters and limits used.	**Supplement**
Selection process	8	Specify the methods used to decide whether a study met the inclusion criteria of the review, including how many reviewers screened each record and each report retrieved, whether they worked independently, and if applicable, details of automation tools used in the process.	**Page 7 – 8**
Data collection process	9	Specify the methods used to collect data from reports, including how many reviewers collected data from each report, whether they worked independently, any processes for obtaining or confirming data from study investigators, and if applicable, details of automation tools used in the process.	**Page 8 – 9**
Data items	10a	List and define all outcomes for which data were sought. Specify whether all results that were compatible with each outcome domain in each study were sought (e.g. for all measures, time points, analyses), and if not, the methods used to decide which results to collect.	**Page 7**
	10b	List and define all other variables for which data were sought (e.g. participant and intervention characteristics, funding sources). Describe any assumptions made about any missing or unclear information.	**Page 8 – 9**
Study risk of bias assessment	11	Specify the methods used to assess risk of bias in the included studies, including details of the tool(s) used, how many reviewers assessed each study and whether they worked independently, and if applicable, details of automation tools used in the process.	**Page 8**
Effect measures	12	Specify for each outcome the effect measure(s) (e.g. risk ratio, mean difference) used in the synthesis or presentation of results.	**Page 9**
Synthesis methods	13a	Describe the processes used to decide which studies were eligible for each synthesis (e.g. tabulating the study intervention characteristics and comparing against the planned groups for each synthesis (item #5)).	**Not Applicable**
	13b	Describe any methods required to prepare the data for presentation or synthesis, such as handling of missing summary statistics, or data conversions.	**Not Applicable**
	13c	Describe any methods used to tabulate or visually display results of individual studies and syntheses.	**Not Applicable**
	13d	Describe any methods used to synthesize results and provide a rationale for the choice(s). If meta-analysis was performed, describe the model(s), method(s) to identify the presence and extent of statistical heterogeneity, and software package(s) used.	**Not Applicable**
	13e	Describe any methods used to explore possible causes of heterogeneity among study results (e.g. subgroup analysis, meta-regression).	**Not Applicable**
	13f	Describe any sensitivity analyses conducted to assess robustness of the synthesized results.	**Not Applicable**
Reporting bias assessment	14	Describe any methods used to assess risk of bias due to missing results in a synthesis (arising from reporting biases).	**Not Applicable**
Certainty assessment	15	Describe any methods used to assess certainty (or confidence) in the body of evidence for an outcome.	**Not Applicable**
**RESULTS**			

For peer review only

PRISMA 2020 Checklist

Section and Topic	Item #	Checklist item	Location where item is reported
Study selection	16a	Describe the results of the search and selection process, from the number of records identified in the search to the number of studies included in the review, ideally using a flow diagram.	**Page 9 & Figure 1**
	16b	Cite studies that might appear to meet the inclusion criteria, but which were excluded, and explain why they were excluded.	**Not applicable**
Study characteristics	17	Cite each included study and present its characteristics.	**Table 1 and Table 2 & Supplement Tables S4 and S7**
Risk of bias in studies	18	Present assessments of risk of bias for each included study.	**Supplement Tables S1 – S3**
Results of individual studies	19	For all outcomes, present, for each study: (a) summary statistics for each group (where appropriate) and (b) an effect estimate and its precision (e.g. confidence/credible interval), ideally using structured tables or plots.	**(a) Supplement Tables S5, S6, S8, S9**<br>**(b) Not applicable**
Results of syntheses	20a	For each synthesis, briefly summarise the characteristics and risk of bias among contributing studies.	**Not applicable**
	20b	Present results of all statistical syntheses conducted. If meta-analysis was done, present for each the summary estimate and its precision (e.g. confidence/credible interval) and measures of statistical heterogeneity. If comparing groups, describe the direction of the effect.	**Not applicable**
	20c	Present results of all investigations of possible causes of heterogeneity among study results.	**Not applicable**
	20d	Present results of all sensitivity analyses conducted to assess the robustness of the synthesized results.	**Not applicable**
Reporting biases	21	Present assessments of risk of bias due to missing results (arising from reporting biases) for each synthesis assessed.	**Not applicable**
Certainty of evidence	22	Present assessments of certainty (or confidence) in the body of evidence for each outcome assessed.	**Not applicable**
**DISCUSSION**			
Discussion	23a	Provide a general interpretation of the results in the context of other evidence.	**Page 18 – 24**
	23b	Discuss any limitations of the evidence included in the review.	**Page 18 – 24**
	23c	Discuss any limitations of the review processes used.	**Not applicable**
	23d	Discuss implications of the results for practice, policy, and future research.	**Page 18 – 24**
**OTHER INFORMATION**			
Registration and protocol	24a	Provide registration information for the review, including register name and registration number, or state that the review was not registered.	**Abstract & Methods**
	24b	Indicate where the review protocol can be accessed, or state that a protocol was not prepared.	**Abstract & Methods**
	24c	Describe and explain any amendments to information provided at registration or in the protocol.	**Not applicable**
Support	25	Describe sources of financial or non-financial support for the review, and the role of the funders or sponsors in the review.	**Not applicable**
Competing interests	26	Declare any competing interests of review authors.	**Page 26**
Availability of data, code and other materials	27	Report which of the following are publicly available and where they can be found: template data collection forms; data extracted from included studies; data used for all analyses; analytic code; any other materials used in the review.	**Not applicable**

From: Page MJ, McKenzie JE, Bossuyt PM, Boutron I, Hoffmann TC, Mulrow CD, et al. The PRISMA 2020 statement: an updated guideline for reporting systematic reviews. BMJ 2021;372:n71. doi: 10.1136/bmj.n71

For more information, visit: http://www.prisma-statement.org/

For peer review only - http://bmjopen.bmj.com/site/about/guidelines.xhtml
